# Supplementary material for: IFN-γ-Induced intestinal epithelial cell-type-specific programmed cell death: PANoptosis and its modulation in Crohn’s disease
Source: Front Immunol. 2025 Mar 31;16:1523984. doi: 10.3389/fimmu.2025.1523984 (PMC11994596; doi:10.3389/fimmu.2025.1523984)

Supplementary Materials

Supplementary Material S1. (A) Confocal microscopy image of the EdU assay on the Ctrl enteroid treated with different concentrations of IFN-γ. (B) Representative confocal microscopy image of patient-derived enteroid of similar shape and size treated with and without IFN-γ. Scale bar = 50 μm.

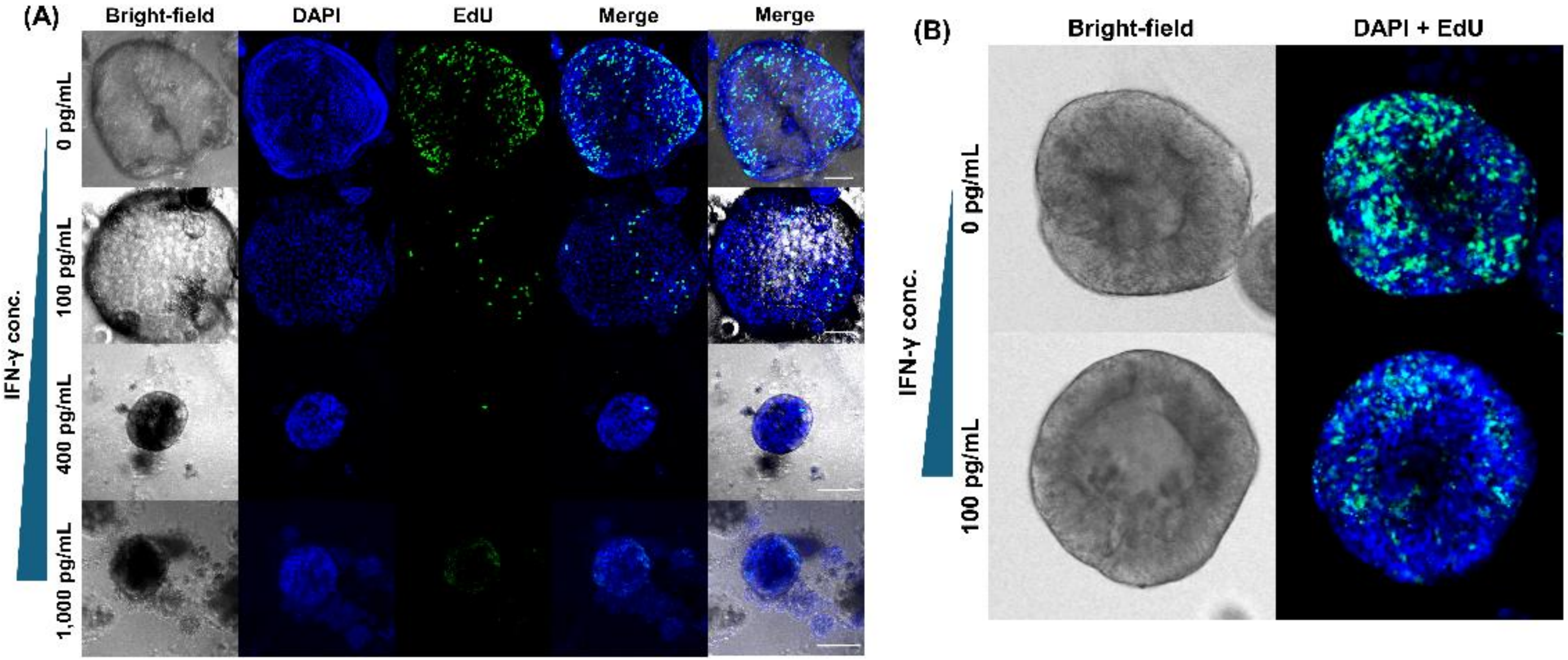

Supplementary Material S2. Gene expression associated with programmed cell death that changed with IFN-γ treatment in human enteroids

		Mean FPKM of IFN-γ-free enteroids	S.D. of FPKM for IFN-γ-free enteroids	Mean FPKM of IFN-γ-treated enteroids	S.D. of FPKM for IFN-γ-treated enteroids	log2FC	*p*-value	Adj. *p*-value	FDR *q*-value
	Signifcant gene in *t*-test ($p < 0.01$, adj. $p < 0.01$, and $q < 0.05$)								
TP53		71.009	5.777	62.754	4.723	-0.18	1.33E-04	4.00E-03	2.35E-04
BCL2L1		536.495	31.299	480.732	27.277	-0.16	9.01E-06	2.70E-04	1.69E-05
MCL1		342.195	18.222	454.584	24.192	0.41	1.67E-14	5.00E-13	1.25E-13
BAK1		135.437	8.224	248.330	16.741	0.87	0.00E+00	0.00E+00	0.00E+00
CASP8		44.984	3.411	69.869	6.637	0.64	5.08E-12	1.52E-10	1.90E-11
CASP10		36.503	4.016	80.381	8.020	1.14	2.00E-15	6.00E-14	2.00E-14
CASP3		74.142	4.586	93.636	6.338	0.34	1.52E-10	4.57E-09	4.57E-10
CASP7		110.490	10.833	169.832	12.911	0.62	1.69E-14	5.06E-13	1.01E-13
TNFSF10		109.138	17.094	253.148	39.849	1.21	2.21E-11	6.62E-10	7.36E-11
TNFRSF1B		46.301	6.722	83.895	7.954	0.86	8.88E-15	2.58E-13	6.44E-14
FAS		56.749	4.955	75.145	6.632	0.41	1.67E-09	5.00E-08	4.17E-09
CYLD		30.903	2.973	48.965	4.100	0.66	4.31E-14	1.29E-12	2.15E-13
RIPK1		66.665	4.017	77.999	4.519	0.23	2.90E-08	8.69E-07	6.21E-08
MLKL		19.901	2.826	40.499	7.110	1.03	1.57E-09	4.72E-08	4.29E-09
CASP1		22.754	4.491	138.742	22.000	2.61	5.79E-13	1.74E-11	2.48E-12
CASP5		11.110	2.856	20.948	5.676	0.91	3.12E-06	9.35E-05	6.23E-06
GSDMD		71.910	6.441	129.459	9.471	0.85	0.00E+00	0.00E+00	0.00E+00
ZBP1		0.908	0.350	15.312	5.015	4.08	8.07E-09	2.42E-07	1.86E-08
	Non-signifcant gene in *t*-test ($p > 0.01$, adj. $p > 0.01$, or $q > 0.05$)								

BAX		130.591	8.723	120.477	8.780	-0.12	0.00278	0.08343	0.00363
CASP9		20.370	0.885	21.080	0.837	0.05	0.02680	0.80389	0.03216
TNFRSF1A		46.301	6.722	83.895	7.954	0.86	0.00257	0.07712	0.00351
FADD		43.530	2.022	43.687	1.933	0.01	0.82357	1.00000	0.82357
RIPK3		42.515	4.997	44.992	4.283	0.08	0.14300	1.00000	0.15322
NLRC4		0.538	0.130	0.639	0.122	0.25	0.03167	0.95010	0.03654
CASP4		86.885	9.398	102.656	15.936	0.24	0.00230	0.06907	0.00329
MAP3K7		40.851	1.987	39.451	1.580	-0.05	0.03566	1.00000	0.03963
PSTPIP2		41.998	2.939	42.775	3.145	0.03	0.47598	1.00000	0.49239

Supplementary Material S3. Pathway analysis and Gene set enrichment analysis

(A) REACTOME pathway analysis

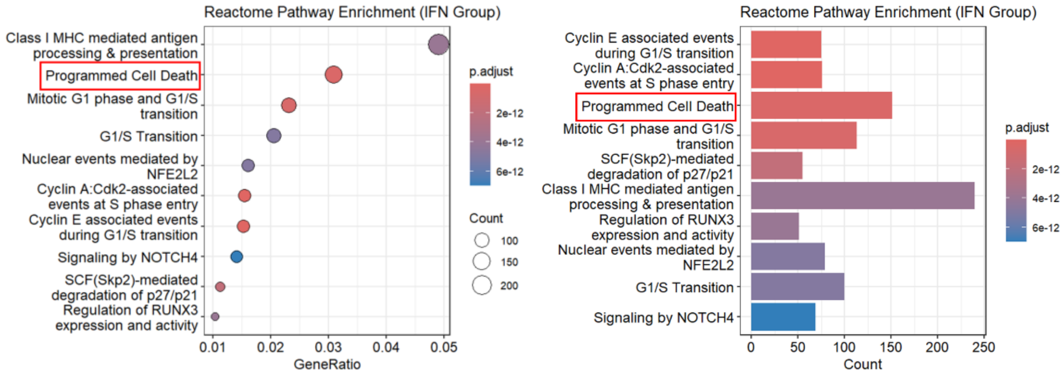

Top 20 REACTOME pathway

ID	Description	Gene Ratio	Bg Ratio	Rich Factor	Fold Enrichment	z-Score	p-value	p. adjust	Gene ID
R-HSA-69202	Cyclin E associated events during G1/S transition	75/4886	83/11146	0.90361	2.06134	8.57403	4.45E-19	7.21E-16	geneID
R-HSA-69656	Cyclin A:Cdk2-associated events at S phase entry	76/4886	85/11146	0.89412	2.03967	8.50037	1.15E-18	9.34E-16	PSMB8/PSMB9/PSME1/PSME2/PSMB10/PSMA4/PSMA1/PSMA6/MAX/UBB/PSMD11/PSMB7/UBC/PSMA3/PSMA2/LIN52/PSMC4/PSMA5/E2F5/PSMB2/PSMA7/PSMD3/PSMC1/PSMF1/PSMB4/PSMB1/CDKN1A/CDK2/CABLES1/PSMB3/MYC/PSMD13/PSMC6/WEE1/PSMB6/PSMD7/RBBP4/PSMD12/PSMD14/PSMB5/PSMD1/RBL2/TFDP2/CCNH/PSME3/PSMC2/PSMD2/CDC25A/PSMD6/PSMC3/RB1/E2F4/E2F1/CDK7/PSMD5/CCND1/PSMD9/CDK4/PSMD4/LIN37/LIN9/PTK6/CDKN1B/CCNE2/TFDP1/LIN54/SKP1/PSMC5/PSMD8/UBA52/SKP2/RPS27A/CKS1B/CCNE1/MNAT1
R-HSA-5357801	Programmed Cell Death	151/4886	213/11146	0.70892	1.61720	8.03477	7.08E-16	3.82E-13	PSMB8/PSMB9/PSME1/CDC25B/PSME2/PSMB10/PSMA4/PSMA1/PSMA6/MAX/UBB/PSMD11/PSMB7/UBC/PSMA3/PSMA2/LIN52/PSMC4/PSMA5/E2F5/PSMB2/PSMA7/PSMD3/PSMC1/PSMF1/PSMB4/PSMB1/CDKN1A/CDK2/CABLES1/PSMB3/MYC/PSMD13/PSMC6/WEE1/PSMB6/PSMD7/RBBP4/PSMD12/PSMD14/PSMB5/PSMD1/RBL2/TFDP2/CCNH/PSME3/PSMC2/PSMD2/CDC25A/PSMD6/PSMC3/RB1/E2F4/E2F1/CDK7/PSMD5/CCND1/PSMD9/CDK4/PSMD4/LIN37/LIN9/PTK6/CDKN1B/CCNE2/TFDP1/LIN54/SKP1/PSMC5/PSMD8/UBA52/SKP2/RPS27A/CKS1B/CCNE1/MNAT1
R-HSA-453279	Mitotic G1 phase and G1/S transition	113/4886	149/11146	0.75839	1.73005	7.92567	1.29E-15	5.21E-13	PSMB8/PSMB9/PSME1/IRF1/PSME2/PSMB10/CASP1/BAK1/PSMA4/PSMA1/PSMA6/GSDMD/UBB/PSMD11/PSMB7/PMAIP1/CASP8/UBC/PSMA3/TNFSF10/DYNLL2/BIRC3/PSMA2/CASP7/PSMC4/DAPK1/MLKL/STAT3/PSMA5/PSMB2/TICAM1/PSMA7/IRF2/CASP3/PSMD3/FAS/PSMC1/PSMF1/PSMB4/GSN/PPP3CC/PSMB1/TP63/CDKN2A/NMT1/PSMB3/AVEN/CYCS/PSMD13/PSMC6/PSMB6/PSMD7/STK24/RIPK1/PSMD12/UACA/PSMD14/PSMB5/CFLAR/HMGB1/PSMD1/PTK2/MAGED1/PLEC/YWHAB/CASP5/CASP6/ARHGAP10/BMF/TFDP2/BAD/APPL1/DAPK2/TJP2/PSME3/C1QBP/YWHAQ/PSMC2/PPP3R1/DNM1L/KPNA1/CASP9/PSMD2/TLR4/UBE2L3/SATB1/BID/PSMD6/CARD8/CLSPN/PSMC3/CDC37/STUB1/BIRC2/CASP4/OCLN/PAK2/E2F1/CHMP4B/IL18/KPNB1/DCC/TP73/PSMD5/HMGB2/DAPK3/FADD/PSMD9/PSMD4/UNC5B/DIABLO/TRAF2/LMNB1/YWHAZ/XIAP/FNTA/SFN/CHMP4A/IL1A/CHMP3/TFDP1/TNFRSF10B/CHMP4C/SDCBP/BBC3/DBNL/IL1B/ROCK1/BCL2L11/RIPK3/ADD1/YWHAG/OMA1/PSMC5/HSP90AA1/PSMD8/UNC5A/MAPK8/UBA52/PPP1R13B/OPA1/PDCD6IP/CHMP6/CHMP7/PRKCQ/BCAP31/RPS27A/APIP/OGT/CHMP2A/DFFB
R-HSA-187577	SCF(Skp2)-mediated degradation of p27/p21	55/4886	60/11146	0.91667	2.09111	7.48663	5.65E-15	1.83E-12	PSMB8/PSMB9/PSME1/PSME2/PSMB10/PSMA4/PSMA1/PSMA6/JAK2/MAX/UBB/LYN/PSMD11/PSMB7/UBC/PSMA3/PSMA2/LIN52/PSMC4/PSMA5/E2F5/PSMB2/PSMA7/PSMD3/PSMC1/PSMF1/PSMB4/PSMB1/CDKN1A/CDK2/CDKN2A/CABLES1/PSMB3/MYC/PPP2CA/PSMD13/CCND3/PSMC6/WEE1/PSMB6/PSMD7/RPA1/RBBP4/PSMD12/PSMD14/PSMB5/GMNN/MCM7/CCND2/E2F3/PSMD1/CDKN2D/RBL1/TYMS/RBL2/TFDP2/PPP2CB/CCNH/PSME3/PSMC2/MCM5/PSMD2/CDC25A/DYRK1A/TK1/PSMD6/POLE2/MCM8/FBXO5/PSMC3/RB1/POLA1/POLE4/E2F4/E2F1/CDK7/PSMD5/CCND1/POLE/PSMD9/MYBL2/CDK4/PSMD4/ORC2/MCM10/LIN37/LIN9/MCM2/PTK6/CDKN1B/CDC45/CCNE2/TFDP1/ORC5/POLE3/MCM4/LIN54/E2F2/SKP1/SRC/CDKN2C/PSMC5/PSMD8/CDC6/UBA52/ORC4/SKP2/RRM2/RPS27A/CDKN2B/CKS1B/CCNE1/MNAT1

R-HSA-983169	Class I MHC mediated antigen processing & presentation	240/4886	381/11146	0.62992	1.43698	7.66744	1.60E-14	3.97E-12	PSMB8/PSMB9/PSME1/PSME2/PSMB10/PSMA4/PSMA1/PSMA6/UBB/PSMD11/PSMB7/UBC/PSMA3/PSMA2/PSMC4/PSMA5/PSMB2/PSMA7/PSMD3/PSMC1/PSMF1/PSMB4/PSMB1/CDKN1A/CDK2/PSMB3/PSMD13/PSMC6/PSMB6/PSMD7/PSMD12/PSMD14/PSMB5/PSMD1/PSME3/PSMC2/PSMD2/PSMD6/PSMC3/PSMD5/CCND1/PSMD9/CDK4/PSMD4/PTK6/CDKN1B/CCNE2/SKP1/PSMC5/PSMD8/UBA52/SKP2/RPS27A/CKS1B/CCNE1
R-HSA-8941858	Regulation of RUNX3 expression and activity	51/4886	55/11146	0.92727	2.11531	7.32520	1.71E-14	3.97E-12	TAP1/PSMB8/PSMB9/HLA-E/TAP2/PSME1/B2M/PSME2/PSMB10/HLA-A/UBE2L6/HLA-C/HLA-B/PSMA4/PSMA1/HLA-F/DTX3L/PSMA6/RNF213/CTSS/UBB/PSMD11/PSMB7/FBXO6/TRIM21/ERAP2/FBXO7/UBR2/TRIM69/UBC/PSMA3/RNF114/CUL7/PSMA2/KCTD7/UBA6/PSMC4/CUL2/ITGB5/RBCK1/PSMA5/UBOX5/PSMB2/IKBKG/RNF19B/HERC6/TAPBP/PSMA7/UBA7/UBE2Z/PSMD3/KLHL22/SEC24C/PSMC1/PSMF1/PSMB4/ASB1/UBE2D3/ERAP1/PDIA3/PSMB1/VAMP3/MKRN1/KLHL5/FBXO2/ZNRF1/PSMB3/STX4/MYD88/FBXL5/PIK3C3/PSMD13/SEC31A/ITGAV/FBXO31/CDC27/PSMC6/RNF130/PSMB6/SOCS1/PSMD7/RNF34/CUL3/KLHL3/PSMD12/VHL/PSMD14/LMO7/PSMB5/RNF115/UBE2E3/FBXO21/TRIM11/HMGB1/FBXO10/PSMD1/UBE2M/SEC24A/KLHL21/ASB7/UBE3B/VAMP8/HERC1/FBXO4/UBE2Q1/PSME3/UBE2V2/ASB14/ATG14/PSMC2/HLA-G/RNF123/RNF217/RNF126/TRIP12/UBE2J2/SEC61B/PSMD2/TLR4/UBE2L3/HERC4/UBE2Q2/UBE2F/RNF6/LONRF1/PSMD6/DZIP3/HERC5/KEAP1/UBE2G1/PSMC3/CBLB/TRAF7/STUB1/CDC16/UBE2E1/RNF7/UBE2O/SOCS3/ASB8/ANAPC2/HECTD3/KLHL2/RBX1/FBXO41/RNF4/CALR/UBE2D2/RLIM/LRSAM1/SEC22B/UBE4A/FBXW7/RNF144B/IKBKB/SEC24B/LRRC41/PSMD5/HACE1/ASB6/UBE2N/PSMD9/RNF19A/LNPEP/FBXO44/KLHL13/UNKL/KCTD6/FBXL18/PSMD4/TRIM37/UBE2E2/SEC23A/UBE3D/CHUK/FBXO9/TRAIP/BLMH/NCF1/CYBB/DET1/FBXW2/S100A1/ANAPC10/ANAPC13/CDC34/RNF14/RNF138/TRIM32/FBXO22/SEC13/RCHY1/FBXW10/SAR1B/THOP1/FBXL20/SKP1/UBE2K/KBTBD8/FBXW11/HERC3/UBA3/ATG7/SPSB1/FBXL3/FBXO32/PSMC5/FBXL12/PSMD8/FBXW5/MYLIP/WSB1/UBR4/RNF41/UBA52/ZNRF2/TIRAP/UBA1/SKP2/KLHL25/ANAPC5/HSPA5/MIB2/MGRN1/BCAP31/CYBA/CCNF/FBXW9/RPS27A/ARIH2/UBA5/FBXL22/FBXL19/FBXL15/FBXL13/FBXL14/CDC26/SIAH1/FBXL8/RNF25
R-HSA-9759194	Nuclear events mediated by NFE2L2	79/4886	97/11146	0.81443	1.85789	7.49700	2.50E-14	4.87E-12	PSMB8/PSMB9/PSME1/PSME2/PSMB10/PSMA4/PSMA1/PSMA6/UBB/PSMD11/PSMB7/UBC/PSMA3/PSMA2/PSMC4/PSMA5/PSMB2/PSMA7/PSMD3/PSMC1/PSMF1/PSMB4/PSMB1/CDKN2A/TGFB1/PSMB3/MDM2/PSMD13/PSMC6/PSMB6/PSMD7/PSMD12/PSMD14/PSMB5/PSMD1/PSME3/PSMC2/PSMD2/PSMD6/PSMC3/PSMD5/PSMD9/PSMD4/SRC/PSMC5/CBFB/PSMD8/UBA52/RUNX3/RPS27A/EP300
R-HSA-69206	G1/S Transition	100/4886	131/11146	0.76336	1.74138	7.54077	2.71E-14	4.87E-12	PSMB8/PSMB9/PSME1/PSME2/PSMB10/PSMA4/PSMA1/PSMA6/UBB/PSMD11/PSMB7/UBC/PSMA3/PSMA2/PSMC4/PSMA5/PSMB2/PSMA7/GSTA1/PSMD3/PSMC1/PSMF1/PSMB4/CCL2/PSMB1/CDKN2A/AREG/PSMB3/RELA/MYC/PSMD13/PSMC6/BACH1/PSMB6/ABCC3/PSMD7/SRXN1/PSMD12/PSMD14/PSMB5/PSMD1/GCLM/SQSTM1/ME1/GCLC/PSME3/PSMC2/TXN/TXNRD1/PSMD2/SP1/PRKAA2/PSMD6/KEAP1/MAFG/PSMC3/TALDO1/RBX1/SLC7A11/ATF4/NQO1/PSMD5/PSMD9/PSMD4/PGD/NFKB1/ABCC1/ABCF2/GSR/SKP1/G6PD/PSMC5/PSMD8/UBA52/TKT/RPS27A/MAFK/NOTCH1/EP300
R-HSA-9013694	Signaling by NOTCH4	69/4886	82/11146	0.84146	1.91956	7.38344	4.57E-14	6.99E-12	PSMB8/PSMB9/PSME1/PSME2/PSMB10/PSMA4/PSMA1/PSMA6/MAX/UBB/PSMD11/PSMB7/UBC/PSMA3/PSMA2/LIN52/PSMC4/PSMA5/E2F5/PSMB2/PSMA7/PSMD3/PSMC1/PSMF1/PSMB4/PSMB1/CDKN1A/CDK2/CABLES1/PSMB3/MYC/PPP2CA/PSMD13/PSMC6/WEE1/PSMB6/PSMD7/RPA1/RBBP4/PSMD12/PSMD14/PSMB5/GMNN/MCM7/PSMD1/RBL1/TYMS/RBL2/TFDP2/PPP2CB/CCNH/PSME3/PSMC2/MCM5/PSMD2/CDC25A/TK1/PSMD6/POLE2/MCM8/FBXO5/PSMC3/RB1/POLA1/POLE4/E2F4/E2F1/CDK7/PSMD5/CCND1/POLE/PSMD9/CDK4/PSMD4/ORC2/MCM10/LIN37/LIN9/MCM2/PTK6/CDKN1B/CDC45/CCNE2/TFDP1/ORC5/POLE3/MCM4/LIN54/SKP1/PSMC5/PSMD8/CDC6/UBA52/ORC4/SKP2/RRM2/RPS27A/CKS1B/CCNE1/MNAT1
R-HSA-69242	S Phase	118/4886	162/11146	0.72840	1.66162	7.49407	4.74E-14	6.99E-12	PSMB8/PSMB9/PSME1/PSME2/PSMB10/PSMA4/PSMA1/PSMA6/UBB/PSMD11/PSMB7/UBC/PSMA3/PSMA2/APH1B/SMAD3/PSMC4/PSMA5/PSMB2/PSMA7/PSMD3/PSMC1/PSMF1/PSMB4/PSMB1/PSENEN/PSMB3/PSMD13/PSMC6/PSMB6/PSMD7/SNW1/PSMD12/PSMD14/PSMB5/PSMD1/PSEN2/RBPJ/APH1A/PSME3/PSMC2/PSMD2/PSMD6/PSMC3/JAG1/RBX1/NOTCH4/ADAM10/MAML3/FBXW7/NCSTN/PSMD5/PSMD9/PSEN1/PSMD4/NOTCH2/KAT2B/YWHAZ/SKP1/DLL4/PSMC5/PSMD8/UBA52/MAML1/MAML2/HES1/RPS27A/NOTCH1/EP300
R-HSA-450408	AUF1 (hnRNP D0) binds and destabilizes mRNA	51/4886	56/11146	0.91071	2.07753	7.14148	1.10E-13	1.49E-11	PSMB8/PSMB9/PSME1/CDC25B/PSME2/PSMB10/PSMA4/PSMA1/PSMA6/MAX/UBB/PSMD11/PSMB7/UBC/PSMA3/PSMA2/LIN52/PSMC4/PSMA5/E2F5/PSMB2/PSMA7/PSMD3/PSMC1/PSMF1/PSMB4/PSMB1/CDKN1A/CDK2/CABLES1/PSMB3/MYC/PSMD13/CDC27/PSMC6/WEE1/PSMB6/PSMD7/RPA1/RBBP4/PSMD12/PSMD14/PSMB5/GMNN/MCM7/PSMD1/RBL2/TFDP2/CCNH/PSME3/PSMC2/MCM5/RFC2/ANAPC16/POLD1/PSMD2/CDC25A/PDS5B/PSMD6/POLE2/LIG1/MCM8/PSMC3/RB1/CDC16/UBE2E1/POLA1/ANAPC2/GINS2/RBX1/FEN1/POLE4/E2F4/E2F1/CDK7/PSMD5/CCND1/POLE/PSMD9/CDK4/PSMD4/ORC2/RFC5/LIN37/LIN9/GINS4/POLD3/MCM2/PTK6/CDKN1B/CDC45/ANAPC10/CCNE2/TFDP1/ORC5/POLE3/ESCO2/MCM4/CDCA5/LIN54/RFC4/SKP1/RFC3/PSMC5/PSMD8/CDC6/UBA52/ORC4/GINS3/SKP2/ANAPC5/POLD4/RPS27A/CKS1B/CDC26/SMC1A/CCNE1/MNAT1
R-HSA-9020702	Interleukin-1 signaling	89/4886	115/11146	0.77391	1.76546	7.28942	1.75E-13	2.18E-11	PSMB8/PSMB9/PSME1/PSME2/PSMB10/PSMA4/PSMA1/PSMA6/UBB/PSMD11/PSMB7/HSPB1/UBC/PSMA3/PSMA2/PSMC4/PSMA5/PSMB2/PSMA7/PSMD3/PSMC1/PSMF1/PSMB4/PSMB1/HSPA1A/PSMB3/PSMD13/PSMC6/PSMB6/PSMD7/PSMD12/PSMD14/PSMB5/PSMD1/EIF4G1/PSME3/PSMC2/HSPA1B/PSMD2/PSMD6/PSMC3/PSMD5/PSMD9/PSMD4/PABPC1/PSMC5/PSMD8/UBA52/HSPA8/RPS27A/HNRNPD
R-HSA-8878159	Transcriptional regulation by RUNX3	77/4886	96/11146	0.80208	1.82972	7.21301	2.53E-13	2.93E-11	PSMB8/NLRC5/PSMB9/PSME1/PSME2/PSMB10/PSMA4/PSMA1/PSMA6/UBB/PSMD11/PSMB7/CASP8/UBC/PSMA3/PSMA2/NOD1/ALPK1/SAA1/PSMC4/USP18/IL1R2/PSMA5/PSMB2/IKBKG/PSMA7/TRAF6/PSMD3/PSMC1/PSMF1/PSMB4/APP/PSMB1/RIPK2/PSMB3/MYD88/RELA/IL1RAP/PSMD13/PSMC6/PSMB6/PSMD7/IRAK1/PSMD12/PSMD14/MAP3K8/PSMB5/PELI3/HMGB1/PSMD1/NKIRAS1/SQSTM1/NLRX1/PSME3/PSMC2/MAP3K3/PSMD2/PSMD6/IL1RN/PSMC3/RBX1/IKBKB/PSMD5/UBE2N/PSMD9/NKIRAS2/PSMD4/NFKB1/CHUK/TRAF2/LRRC14/MAP2K4/IL1A/MAP2K6/USP14/SKP1/IL1B/FBXW11/PSMC5/PSMD8/TAB3/UBA52/TAB2/MAP3K7/RPS27A/TOLLIP/IKBIP/TAB1/PELI2
R-HSA-9010553	Regulation of expression of SLITs and ROBOs	122/4886	172/11146	0.70930	1.61807	7.21686	4.14E-13	4.47E-11	PSMB8/PSMB9/PSME1/PSME2/PSMB10/PSMA4/PSMA1/PSMA6/UBB/PSMD11/PSMB7/UBC/PSMA3/PSMA2/SMAD3/PSMC4/TEAD2/PSMA5/PSMB2/PSMA7/PSMD3/PSMC1/PSMF1/PSMB4/PSMB1/CDKN1A/CDKN2A/TGFB1/PSMB3/BRD2/MDM2/MYC/PSMD13/PSMC6/PSMB6/PSMD7/SNW1/PSMD12/PSMD14/PSMB5/TCF7L1/PSMD1/RUNX1/RBPJ/YAP1/PSME3/FOXO3/PSMC2/PSMD2/PSMD6/PSMC3/ZFHX3/SMAD4/JAG1/MAML3/PSMD5/CCND1/TCF7L2/PSMD9/PSMD4/KAT2B/TEAD1/KRAS/SRC/BCL2L11/PSMC5/CBFB/PSMD8/UBA52/MAML1/RUNX3/TEAD4/MAML2/HES1/RPS27A/NOTCH1/EP300
R-HSA-9604323	Negative regulation of NOTCH4 signaling	49/4886	54/11146	0.90741	2.06999	6.96307	4.81E-13	4.87E-11	PSMB8/PSMB9/PSME1/PSME2/PSMB10/PSMA4/PSMA1/PSMA6/UBB/PSMD11/PSMB7/UBC/PSMA3/PSMA2/PSMC4/CUL2/PSMA5/PSMB2/PSMA7/PSMD3/PSMC1/PSMF1/PSMB4/PSMB1/MSI1/PSMB3/RPL3/PSMD13/PSMC6/RPS6/PSMB6/PSMD7/PSMD12/ETF1/PSMD14/RPL10A/PSMB5/RPL13A/RPL5/RPS3A/USP33/PSMD1/EIF4G1/RPL15/RPL7/RPL23/RPL31/RPL12/DAG1/UPF2/RPS3/PSME3/PSMC2/NCBP2/PSMD2/RPS4X/RPL26/RPL34/RPL7A/PSMD6/RPL6/PSMC3/RPS27/LDB1/RPL22/RPL30/RPL4/EIF4A3/RPL11/RPS9/RBX1/RPS15A/RPS7/RPS14/RPS8/RPL14/RPL10/RPLP0/PSMD5/GSPT2/RPL37A/PSMD9/RPL41/UPF3A/PSMD4/RPL39/ROBO3/RPL9/PABPC1/RPL35A/RPL8/RPL32/RPL36AL/RPL26L1/RPS23/RPS28/RPS15/RPL37/RPLP1/RPL24/RPS25/MAGOH/RPL13/RPS24/RPL18/RPL36/RPS10/RBM8A/LHX4/PSMC5/PSMD8/UBA52/RNPS1/HOXA2/RPS16/RPL27A/RPS5/FAU/RPS2/RPS27A/RPL29/RPL38

R-HSA-376176	Signaling by ROBO receptors	148/4886	219/11146	0.67580	1.54164	7.15175	7.28E-13	6.94E-11	PSMB8/PSMB9/PSME1/PSME2/PSMB10/PSMA4/PSMA1/PSMA6/UBB/PSMD11/PSMB7/UBC/PSMA3/PSMA2/PSMC4/PSMA5/PSMB2/PSMA7/PSMD3/PSMC1/PSMF1/PSMB4/PSMB1/PSMB3/PSMD13/PSMC6/PSMB6/PSMD7/PSMD12/PSMD14/PSMB5/PSMD1/PSME3/PSMC2/PSMD2/PSMD6/PSMC3/RBX1/NOTCH4/FBXW7/PSMD5/PSMD9/PSMD4/YWHAZ/SKP1/PSMC5/PSMD8/UBA52/RPS27A
R-HSA-109581	Apoptosis	126/4886	180/11146	0.70000	1.59685	7.13191	7.95E-13	7.16E-11	PSMB8/PSMB9/PSME1/PSME2/PSMB10/PSMA4/PSMA1/PSMA6/UBB/PSMD11/PSMB7/SRGAP2/UBC/PSMA3/PSMA2/PSMC4/CUL2/PSMA5/PSMB2/PSMA7/PSMD3/PSMC1/PSMF1/PSMB4/GPC1/PSMB1/MSI1/PSMB3/RPL3/FLRT3/PSMD13/PSMC6/RPS6/PSMB6/PSMD7/PSMD12/PRKACB/ETF1/PSMD14/RPL10A/PSMB5/RPL13A/RPL5/RPS3A/USP33/PSMD1/EIF4G1/NELL2/RPL15/RPL7/RPL23/RPL31/CXCR4/ENAH/RPL12/DAG1/UPF2/SOS1/RPS3/PAK1/PSME3/AKAP5/PSMC2/NCBP2/PAK6/PSMD2/RPS4X/PFN2/RPL26/SRGAP1/RPL34/RPL7A/PSMD6/RPL6/PFN1/PSMC3/RPS27/LDB1/RPL22/RPL30/RPL4/EIF4A3/RPL11/RPS9/RBX1/RPS15A/RPS7/RPS14/PAK2/RPS8/RPL14/RPL10/DCC/RPLP0/PSMD5/GSPT2/RPL37A/PSMD9/RPL41/EVL/UPF3A/PSMD4/RPL39/ROBO3/CAP2/RPL9/PABPC1/RPL35A/RPL8/RPL32/RPL36AL/RPL26L1/RPS23/RPS28/RPS15/RPL37/RPLP1/RPL24/RPS25/MAGOH/RPL13/RPS24/RPL18/SRC/PRKCA/RPL36/CAP1/RPS10/RBM8A/LHX4/PSMC5/PSMD8/UBA52/ABL2/RNPS1/HOXA2/RPS16/RPL27A/RPS5/VASP/NRP1/FAU/PPP3CB/RPS2/RPS27A/RPL29/PRKACA/RPL38
R-HSA-1168372	Downstream signaling events of B Cell Receptor (BCR)	68/4886	83/11146	0.81928	1.86894	7.01979	9.38E-13	8.00E-11	PSMB8/PSMB9/PSME1/PSME2/PSMB10/BAK1/PSMA4/PSMA1/PSMA6/GSDMD/UBB/PSMD11/PSMB7/PMAIP1/CASP8/UBC/PSMA3/TNFSF10/DYNLL2/PSMA2/CASP7/PSMC4/DAPK1/STAT3/PSMA5/PSMB2/TICAM1/PSMA7/CASP3/PSMD3/FAS/PSMC1/PSMF1/PSMB4/GSN/PPP3CC/PSMB1/TP63/CDKN2A/NMT1/PSMB3/AVEN/CYCS/PSMD13/PSMC6/PSMB6/PSMD7/STK24/RIPK1/PSMD12/UACA/PSMD14/PSMB5/CFLAR/HMGB1/PSMD1/PTK2/MAGED1/PLEC/YWHAB/CASP6/ARHGAP10/BMF/TFDP2/BAD/APPL1/DAPK2/TJP2/PSME3/C1QBP/YWHAQ/PSMC2/PPP3R1/DNM1L/KPNA1/CASP9/PSMD2/TLR4/SATB1/BID/PSMD6/CARD8/CLSPN/PSMC3/BIRC2/OCLN/PAK2/E2F1/KPNB1/DCC/TP73/PSMD5/HMGB2/DAPK3/FADD/PSMD9/PSMD4/UNC5B/DIABLO/TRAF2/LMNB1/YWHAZ/XIAP/FNTA/SFN/TFDP1/TNFRSF10B/BBC3/DBNL/ROCK1/BCL2L11/ADD1/YWHAG/OMA1/PSMC5/PSMD8/UNC5A/MAPK8/UBA52/PPP1R13B/OPA1/PRKCQ/BCAP31/RPS27A/APIP/DFFB
R-HSA-169911	Regulation of Apoptosis	48/4886	53/11146	0.90566	2.06600	6.87229	1.00E-12	8.11E-11	PSMB8/PSMB9/PSME1/PSME2/PSMB10/PSMA4/PSMA1/PSMA6/UBB/RASGRP3/PSMD11/PSMB7/CALM3/UBC/PSMA3/PSMA2/PSMC4/PSMA5/PSMB2/IKBKG/PSMA7/PSMD3/PSMC1/PSMF1/PSMB4/PSMB1/NFKBIE/PSMB3/RELA/PSMD13/PSMC6/PSMB6/PSMD7/PSMD12/PSMD14/PSMB5/NFATC2/PSMD1/BCL10/PSME3/PSMC2/PPP3R1/PSMD2/RASGRP1/PSMD6/PSMC3/NFATC1/IKBKB/PSMD5/MALT1/PSMD9/PSMD4/NFKB1/CHUK/PPP3CA/KRAS/CALM1/SKP1/FBXW11/REL/PSMC5/FKBP1A/PSMD8/HRAS/UBA52/PPP3CB/MAP3K7/RPS27A

(B) WikiPathways pathway analysis

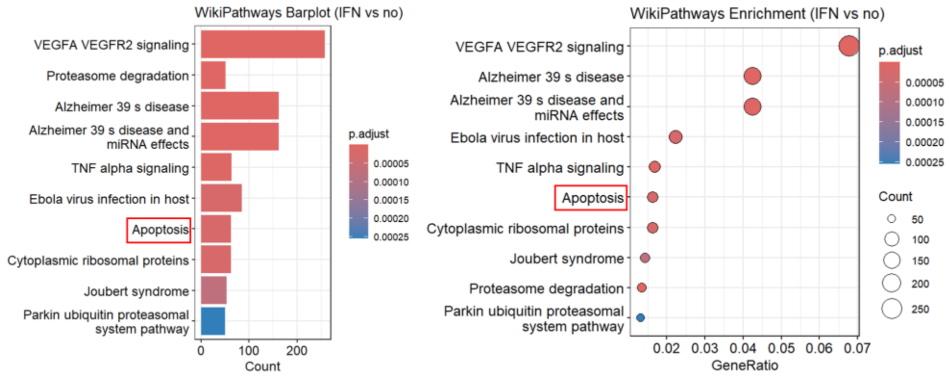

Top 20 WikiPathways signaling

ID	Description	Gene Ratio	Bg Ratio	Rich Factor	Fold Enrichment	z-Score	p-value	p. adjust	Gene ID
WP3888	VEGFA VEGFR2 signaling	258/3807	432/8776	0.59722	1.37673	7.02860	1.92E-12	1.42E-09	6772/3383/4343/5717/3315/3301/57326/25865/3693/160/6774/6648/7170/8775/355/1666/6347/2817/5606/1267/3303/9325/2035/79094/114789/5335/170506/5954/9034/54205/4193/5970/154796/5515/3685/22820/55854/9564/6194/8650/84790/4646/9114/4673/9734/55379/4736/23521/11080/6125/8718/5329/6778/3146/2534/81873/4773/326624/1981/5747/51574/1796/6748/1827/10628/80031/6129/9513/1200/5034/8569/23215/6624/5605/781/2926/1397/4189/3725/1445/2746/5058/4929/2309/5601/3748/9146/7295/4641/8665/8754/5908/9252/84335/5562/2321/10758/8828/9588/32/1847/6154/857/2185/2113/2889/5228/5563/8027/2060/9365/79109/2130/219931/124491/10645/529/7430/9578/56905/7114/1385/1213/682/6729/5216/1398/6464/9451/5743/8575/4772/6902/4217/6747/182/4855/102/811/9261/100506658/7414/8867/5328/27289/10397/5062/468/575

									7/9645/813/4860/5295/5331/23476/595/8772/10525/1465/31/5710/6541/5226/7001/9209/11124/3688/4790/29109/1969/2308/253260/10963/23196/389/51278/5530/653361/1536/9344/26986/10818/5868/5829/8813/23597/2475/23753/23339/1982/9659/6416/274/5608/2081/382/409/10574/23189/6386/2152/56907/10061/3164/5780/867/8936/1977/5590/10056/8550/441478/8974/6093/2317/2876/23291/6714/5578/650/54505/54567/3320/4323/81567/5599/3265/4313/22926/2048/3490/7410/9100/154/7004/3418/4691/6418/8821/10014/4303/7086/29924/3939/83442/824/3486/7024/4633/56999/4627/51763/83473/4952/332/5170/5609/26058
WP183	Proteasome degradation	51/ 3807	62/ 8776	0.82258	1.89624	6.19854	3.22E-10	1.19E-07	5696/5698/3133/5720/5721/5699/3105/3107/3106/5685/5682/3134/5687/7314/5717/5695/7316/5684/5683/5704/5686/5690/5688/7318/5709/5700/5692/7323/5689/5691/5719/5706/5694/5713/5718/5693/5707/10197/5701/3135/5708/9861/5702/7347/7322/5711/5715/5710/5705/5714/7317
WP5124	Alzheimer 39 s disease	162/ 3807	263/ 8776	0.61597	1.41995	6.05222	1.26E-09	3.10E-07	5685/5682/5687/489/9776/5695/808/841/5684/7846/5683/83464/5610/1435/840/3667/5704/4843/5686/5690/5688/1454/836/5709/355/5700/5692/5533/351/1856/5689/823/55851/5691/54205/9706/5970/7417/5289/5719/776/5706/5694/4035/322/5713/27121/84790/5718/8324/7476/10213/5693/23621/54361/11047/5707/5664/91860/7280/3643/7976/51107/5605/572/5293/7124/5332/22863/5701/5534/5601/8408/842/5708/3831/8323/637/1855/1457/4040/9861/1452/7477/5702/8503/9451/292/5291/5743/1649/4217/1965/291/102/7473/7416/7132/468/64837/8313/3551/23385/5295/5331/84617/8772/5715/5663/3709/10383/3028/29982/5710/7494/4790/203068/1147/7186/5530/1536/6622/2475/55626/3552/2081/3845/801/23130/2906/10381/7419/2902/10297/3553/1460/80326/89953/8660/7481/5705/5714/369/5599/3710/3265/22926/4311/3708/10105/7855/348/55102/6868/3799/824/5532/79861/3416/488/2535/5609
WP2059	Alzheimer 39 s disease and miRNA effects	162/ 3807	268/ 8776	0.60448	1.39346	5.72576	8.77E-09	1.62E-06	5685/5682/5687/489/9776/5695/808/841/5684/7846/5683/83464/5610/1435/840/3667/5704/4843/5686/5690/5688/1454/836/5709/355/5700/5692/5533/351/1856/5689/823/55851/5691/54205/9706/5970/7417/5289/5719/776/5706/5694/4035/322/5713/27121/84790/5718/8324/7476/10213/5693/23621/54361/11047/5707/5664/91860/7280/3643/7976/51107/5605/572/5293/7124/5332/22863/5701/5534/5601/8408/842/5708/3831/8323/637/1855/1457/4040/9861/1452/7477/5702/8503/9451/292/5291/5743/1649/4217/1965/291/102/7473/7416/7132/468/64837/8313/3551/23385/5295/5331/84617/8772/5715/5663/3709/10383/3028/29982/5710/7494/4790/203068/1147/7186/5530/1536/6622/2475/55626/3552/2081/3845/801/23130/2906/10381/7419/2902/10297/3553/1460/80326/89953/8660/7481/5705/5714/369/5599/3710/3265/22926/4311/3708/10105/7855/348/55102/6868/3799/824/5532/79861/3416/488/2535/5609
WP231	TNF alpha signaling	64/ 3807	89/ 8776	0.71910	1.65769	5.45837	4.14E-08	6.13E-06	2752/841/330/7133/840/8517/836/4794/5606/29110/5515/8737/1326/8837/10131/6654/572/3725/7124/5790/5601/7295/842/4215/5708/9020/637/1457/11140/7128/4217/329/10010/7132/3551/6610/8772/8567/4214/4790/1147/56616/7186/5836/6416/5608/3845/8844/5590/6500/23291/11035/5966/3320/5599/257397/3265/56957/23118/117584/1535/6885/10454/5609
WP4217	Ebola virus infection in host	85/ 3807	129/ 8776	0.65891	1.51895	5.19717	1.76E-07	2.11E-05	3133/6772/3105/3107/3108/3106/3134/3122/3115/3113/3480/3112/3109/7301/5610/3123/684/4240/2934/153090/29110/7150/65082/5970/7251/3685/27183/2348/57617/3672/55823/3119/7020/5293/2621/5058/708/3135/3111/3836/2318/7099/857/1212/2060/1211/219931/1213/8503/5291/8575/3655/9021/27072/1965/2316/3675/3127/3678/3661/5295/3117/3688/4790/3665/3384/558/389/3385/9367/5868/23339/60/7879/858/5922/2317/5966/4864/7410/8218/3120/6868/3118/2033
WP254	Apoptosis	62/ 3807	88/ 8776	0.70455	1.62414	5.15041	2.27E-07	2.11E-05	3659/834/578/843/5366/841/3480/8743/330/4170/7133/840/8517/3660/836/355/4794/8626/1029/3663/54205/4193/5970/4609/8737/8837/8718/835/665/839/572/3725/7124/27242/842/637/3664/599/3070/329/837/7132/3551/3661/5295/7161/8772/4214/4790/3665/1147/56616/7186/331/666/6416/8795/27113/10018/9169/332/1677
WP477	Cytoplasmic ribosomal proteins	62/ 3807	88/ 8776	0.70455	1.62414	5.15041	2.27E-07	2.11E-05	6122/6194/4736/23521/6125/6189/6138/6195/6129/9349/6160/6136/6188/6191/6154/6164/6130/6128/6232/6146/6156/6124/6135/6203/6210/6201/9801/6208/6202/9045/6134/6175/6168/6171/6196/6199/6170/6133/6165/6132/6161/6228/6234/6209/6167/6176/6152/6230/6137/6229/6141/25873/6204/7311/6217/6157/6193/2197/6187/6233/6159/6169
WP4656	Joubert syndrome	54/ 3807	76/ 8776	0.71053	1.63792	4.88874	8.91E-07	7.33E-05	403/5108/57545/6469/79867/203286/582/5147/79600/26123/6102/4646/5158/51199/4644/4867/27077/1069/261734/55212/27241/1855/117177/9094/200728/5116/9738/2316/51684/4218/468/585/472/23322/142/80210/27130/402/51259/91147/2475/9731/129880/56623/10524/79848/22897/200894/10111/95681/80184/27031/65062/123016
WP2359	Parkin ubiquitin proteasomal system pathway	50/ 3807	71/ 8776	0.70423	1.62340	4.61628	3.44E-06	2.55E-04	834/9246/5717/841/7846/5704/5709/5700/3306/3313/3303/5719/5706/5713/84790/5718/3308/10213/5707/7280/5701/118424/3304/5708/7332/3305/9861/7326/5702/10273/51182/55294/5711/84617/5715/25897/10383/5710/203068/6622/10381/5705/5714/7317/3309/3312/79861/8573/898/6477
WP2004	miR targeted genes in lymphocytes	229/ 3807	424/ 8776	0.54009	1.24504	4.52695	4.21E-06	2.67E-04	79751/103/53/64866/4343/26064/7846/58533/4170/3667/160/51479/8943/7170/10241/57590/347902/3918/8546/9341/2580/1075/1026/1029/79665/5955/10423/27250/64422/2247/283237/9019/51616/10797/571/5887/253558/27230/3783/55503/84705/114793/1326/23621/5937/1871/8202/3475/2589/8774/2771/5878/10802/9513/7298/4552/781/3915/255520/51715/708/79969/10971/5236/2997/5534/7296/10672/9528/83858/1051/8161/3839/6675/5870/7127/51654/54964/5874/10449/391/57678/85440/27236/822/24145/29901/5925/55717/6902/79073/51014/5527/55660/3992/117145/7171/9497/7168/2820/83451/56262/4924/10036/56655/57532/124540/1284/23129/1869/372/5757/9354/285598/5479/4860/10270/1371/55225/595/8683/23224/4643/9076/8636/23589/8036/1465/10857/4774/8649/10282/51673/2673/1788/10484/3784/10611/55722/51125/558/4853/23423/5792/22862/64359/94081/83440/6509/23378/134266/5530/5725/29128/604/90522/80005/1027/23339/4144/84061/2948/4233/5510/8795/6386/7798/9522/3845/55347/5270/546/10061/10634/29099/4580/2483/1977/8801/594/84188/80335/23471/8974/9526/8744/23291/2539/6560/25828/10487/26973/64951/6576/3202/9319/865/29116/10558/23761/9296/27314/57552/9212/79602/9415/8301/6558/60559/4613/290/4691/10105/8487/83442/8829/4651/23647/5139/92935/79188/3159/488/8763/27243/54901/4851/8243/81034
WP4197	Immune response to tuberculosis	20/ 3807	22/ 8776	0.90909	2.09566	4.50365	4.33E-06	2.67E-04	6890/5696/3659/6772/3437/8519/3430/10379/3717/6773/4599/8651/3434/3454/3455/4938/3460/3459/3716/5771
WP5234	Bardet Biedl syndrome	59/ 3807	88/ 8776	0.67045	1.54555	4.50191	5.83E-06	3.32E-04	403/5311/9742/114327/23059/582/5147/6608/57560/167691/6102/23432/79659/51098/28981/1259/55081/54585/84314/5314/2736/79809/5727/51715/92482/55212/80258/27241/112752/11020/51684/57728/51626/374654/8239/4750/585/57539/80173/27130/55764/49855/26146/51259/91147/9731/129880/56623/22897/200894/22954/132320/201163/5310/95681/80184/27031/4952/123016
WP1772	Apoptosis modulation and signaling	61/ 3807	92/ 8776	0.66304	1.52847	4.45997	7.05E-06	3.73E-04	834/578/843/8793/5366/841/9131/8743/330/4170/7133/840/7850/7189/836/355/8682/1029/3303/54205/4615/3654/8737/8837/8718/835/839/9531/90427/572/3725/842/9020/637/826/599/638/4217/329/837/7132/2021/3551/8772/4671/8567/5783/4790/56616/331/666/8795/27113/84883/10018/2353/5599/51651/54472/332/1677
WP45	G1 to S cell cycle control	45/ 3807	64/ 8776	0.70313	1.62086	4.36322	1.13E-05	5.60E-04	1026/1017/1029/148327/4193/4609/896/7465/6117/4176/894/1871/1032/90993/7029/902/4174/993/1388/5427/1385/5925/1869/1022/10488/595/5426/472/84699/1019/4999/1647/4171/1027/8318/9134/7027/5001/4173/1870/1031/5000/1030/898/4331
WP5039	SARS CoV 2 innate immunity evasion and cell specific immune response	46/ 3807	66/ 8776	0.69697	1.60667	4.33010	1.31E-05	5.76E-04	6772/8519/6373/6773/4599/841/3433/23367/4088/7706/79132/7189/6347/2920/29110/7040/7188/6352/8737/3454/7020/4283/3627/7124/9540/2921/3455/5473/682/3570/4928/6372/8480/10010/3066/164/3661/8772/4790/3665/3716/7186/59272/1437/57506/2033

WP2003	miR targeted genes in leukocytes	79/ 3807	127/ 8776	0.62205	1.43396	4.31171	1.32E-05	5.76E-04	103/53/64866/7846/58533/4170/8943/7170/57590/2580/1075/10423/10797/3783/114793/1326/3475/2771/5878/10802/7298/10971/2997/7296/10672/1051/5870/7127/51654/54964/10449/7057/391/822/771/29901/55717/6902/79073/55660/3992/23657/56655/23129/372/5479/4860/8036/1465/8649/10611/51125/23423/94081/83440/6509/604/23339/5270/2483/23471/9526/8744/2539/25828/10487/26973/6576/10558/23761/9296/79602/290/10105/83442/79188/3159/488/8763
WP179	Cell cycle	75/ 3807	120/ 8776	0.62500	1.44077	4.25522	1.71E-05	7.03E-04	994/4088/1875/1026/1017/1029/7040/4193/4609/10459/896/996/7465/1111/4176/894/1871/1032/7529/5933/5934/7029/902/10971/4174/7043/8379/7042/993/5925/8881/4089/29882/9978/1874/1869/3066/1022/595/472/1019/4999/1647/7272/4171/7534/545/2810/1027/8318/10393/25847/9134/7027/5001/4173/1870/6500/1031/7532/11200/990/699/5000/9700/6502/8556/51433/9184/995/7709/1030/2033/8243/898
WP4666	Hepatitis B infection	91/ 3807	151/ 8776	0.60265	1.38924	4.22289	1.90E-05	7.20E-04	6772/843/3717/6773/64135/841/7046/6777/4088/6774/8517/148022/5582/7189/836/355/7098/1026/5606/29110/148327/7040/54205/4615/5970/4609/3654/537/3339/6778/4773/3454/7529/90993/5605/6654/572/3725/5293/7124/10971/5601/7043/842/7042/7099/1388/637/2185/6776/1385/8503/4776/5291/4772/4089/5600/468/10488/3551/3661/5295/8772/84699/4214/1643/4790/3665/1147/3716/7534/6416/5608/3845/9586/7419/6714/5578/369/2353/5599/3265/114609/23118/1642/57506/6885/332/10454/2033/5609
WP1984	Integrated breast cancer pathway	92/ 3807	153/ 8776	0.60131	1.38615	4.21748	1.94E-05	7.20E-04	994/6772/578/4149/25780/5371/9958/841/7046/3667/80271/3612/4091/836/84640/1017/4092/60489/64781/4193/4609/7465/571/2956/1111/55526/5898/23077/8202/9175/2664/27005/1605/572/3725/57820/5058/10557/10600/842/993/637/6667/140739/580/1385/5925/641/4089/4436/5284/7249/1869/6790/1022/5888/7248/595/472/79877/8772/8061/1019/4664/4790/2308/1147/1647/3716/2355/2475/545/3845/659/23613/7035/26284/253959/4953/23411/11200/6597/369/10111/657/8846/79902/3156/466/10454/2033/8438

(C) PathBank pathway analysis

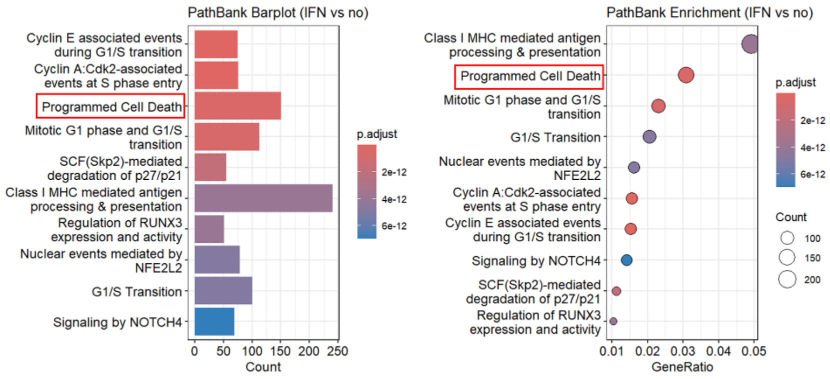

Top 20 PathBank pathway

ID	Description	Gene Ratio	Bg Ratio	Rich Factor	Fold Enrichment	z-Score	p-value	p. adjust	Gene ID
R-HSA-69202	Cyclin E associated events during G1/S transition	75/ 4886	83/ 11146	0.90361	2.06134	8.57403	4.45E-19	7.21E-16	PSMB8/PSMB9/PSME1/PSME2/PSMB10/PSMA4/PSMA1/PSMA6/MAX/UBB/PSMD11/PSMB7/UBC/PSMA3/PSMA2/LIN52/PSMC4/PSMA5/E2F5/PSMB2/PSMA7/PSMD3/PSMC1/PSMF1/PSMB4/PSMB1/CDKN1A/CDK2/CABLES1/PSMB3/MYC/PSMD13/PSMC6/WEE1/PSMB6/PSMD7/RBBP4/PSMD12/PSMD14/PSMB5/PSMD1/RBL2/TFDP2/CCNH/PSME3/PSMC2/PSMD2/CDC25A/PSMD6/PSMC3/RB1/E2F4/E2F1/CDK7/PSMD5/CCND1/PSMD9/CDK4/PSMD4/LIN37/LIN9/PTK6/CDKN1B/CCNE2/TFDP1/LIN54/SKP1/PSMC5/PSMD8/UBA52/SKP2/RPS27A/CKS1B/CCNE1/MNAT1
R-HSA-69656	Cyclin A:Cdk2-associated events at S phase entry	76/ 4886	85/ 11146	0.89412	2.03967	8.50037	1.15E-18	9.34E-16	PSMB8/PSMB9/PSME1/CDC25B/PSME2/PSMB10/PSMA4/PSMA1/PSMA6/MAX/UBB/PSMD11/PSMB7/UBC/PSMA3/PSMA2/LIN52/PSMC4/PSMA5/E2F5/PSMB2/PSMA7/PSMD3/PSMC1/PSMF1/PSMB4/PSMB1/CDKN1A/CDK2/CABLES1/PSMB3/MYC/PSMD13/PSMC6/WEE1/PSMB6/PSMD7/RBBP4/PSMD12/PSMD14/PSMB5/PSMD1/RBL2/TFDP2/CCNH/PSME3/PSMC2/PSMD2/CDC25A/PSMD6/PSMC3/RB1/E2F4/E2F1/CDK7/PSMD5/CCND1/PSMD9/CDK4/PSMD4/LIN37/LIN9/PTK6/CDKN1B/CCNE2/TFDP1/LIN54/SKP1/PSMC5/PSMD8/UBA52/SKP2/RPS27A/CKS1B/CCNE1/MNAT1
R-HSA-5357801	Programmed Cell Death	151/ 4886	213/ 11146	0.70892	1.61720	8.03477	7.08E-16	3.82E-13	PSMB8/PSMB9/PSME1/IRF1/PSME2/PSMB10/CASP1/BAK1/PSMA4/PSMA1/PSMA6/GSDMD/UBB/PSMD11/PSMB7/PMAIP1/CASP8/UBC/PSMA3/TNFSF10/DYNLL2/BIRC3/PSMA2/CASP7/PSMC4/DAPK1/MLKL/STAT3/PSMA5/PSMB2/TICAM1/PSMA7/IRF2/CASP3/PSMD3/FAS/PSMC1/PSMF1/PSMB4/GSN/PPP3CC/PSMB1/TP63/CDKN2A/NMT1/PSMB3/AVEN/CYCS/PSMD13/PSMC6/PSMB6/PSMD7/STK24/RIPK1/PSMD12/UACA/PSMD14/PSMB5/CFLAR/HMGB1/PSMD1/PTK2/MAGED1/PLEC/YWHAB/CASP5/CASP6/ARHGAP10/BMF/TFDP2/BAD/APPL1/DAPK2/TJP2/PSME3/C1QBP/YWHAQ/PSMC2/PPP3R1/DNM1L/KPNA1/CASP9/PSMD2/TLR4/UBE2L3/SATB1/BID/PSMD6/CARD8/CLSPN/PSMC3/CDC37/STUB1/BIRC2/CASP4/OCLN/PAK2/E2F1/CHMP4B/IL18/KPNB1/DCC/TP73/PSMD5/HMGB2/DAPK3/FADD/PSMD9/PSMD4/UNC5B/DIAB

									LO/TRAF2/LMNB1/YWHAZ/XIAP/FNTA/SFN/CHMP4A/IL1A/CHMP3/TFDP1/TNFRSF10B/CHMP4C/SDCBP/BBC3/DBNL/IL1B/ROCK1/BCL2L11/RIPK3/ADD1/YWHAG/OMA1/PSMC5/HSP90AA1/PSMD8/UNC5A/MAPK8/UBA52/PPP1R13B/OPA1/PDCD6IP/CHMP6/CHMP7/PRKCQ/BCAP31/RPS27A/APIP/OGT/CHMP2A/DFFB
R-HSA-453279	Mitotic G1 phase and G1/S transition	113/ 4886	149/ 11146	0.75839	1.73005	7.92567	1.29E-15	5.21E-13	PSMB8/PSMB9/PSME1/PSME2/PSMB10/PSMA4/PSMA1/PSMA6/JAK2/MAX/UBB/LYN/PSMD11/PSMB7/UBC/PSMA3/PSMA2/LIN52/PSMC4/PSMA5/E2F5/PSMB2/PSMA7/PSMD3/PSMC1/PSMF1/PSMB4/PSMB1/CDKN1A/CDK2/CDKN2A/CABLES1/PSMB3/MYC/PPP2CA/PSMD13/CCND3/PSMC6/WEE1/PSMB6/PSMD7/RPA1/RBBP4/PSMD12/PSMD14/PSMB5/GMNN/MCM7/CCND2/E2F3/PSMD1/CDKN2D/RBL1/TYMS/RBL2/TFDP2/PPP2CB/CCNH/PSME3/PSMC2/MCM5/PSMD2/CDC25A/DYRK1A/TK1/PSMD6/POLE2/MCM8/FBXO5/PSMC3/RB1/POLA1/POLE4/E2F4/E2F1/CDK7/PSMD5/CCND1/POLE/PSMD9/MYBL2/CDK4/PSMD4/ORC2/MCM10/LIN37/LIN9/MCM2/PTK6/CDKN1B/CDC45/CCNE2/TFDP1/ORC5/POLE3/MCM4/LIN54/E2F2/SKP1/SRC/CDKN2C/PSMC5/PSMD8/CDC6/UBA52/ORC4/SKP2/RRM2/RPS27A/CDKN2B/CKS1B/CCNE1/MNAT1
R-HSA-187577	SCF(Skp2)-mediated degradation of p27/p21	55/ 4886	60/ 11146	0.91667	2.09111	7.48663	5.65E-15	1.83E-12	PSMB8/PSMB9/PSME1/PSME2/PSMB10/PSMA4/PSMA1/PSMA6/UBB/PSMD11/PSMB7/UBC/PSMA3/PSMA2/PSMC4/PSMA5/PSMB2/PSMA7/PSMD3/PSMC1/PSMF1/PSMB4/PSMB1/CDKN1A/CDK2/PSMB3/PSMD13/PSMC6/PSMB6/PSMD7/PSMD12/PSMD14/PSMB5/PSMD1/PSME3/PSMC2/PSMD2/PSMD6/PSMC3/PSMD5/CCND1/PSMD9/CDK4/PSMD4/PTK6/CDKN1B/CCNE2/SKP1/PSMC5/PSMD8/UBA52/SKP2/RPS27A/CKS1B/CCNE1
R-HSA-983169	Class I MHC mediated antigen processing & presentation	240/ 4886	381/ 11146	0.62992	1.43698	7.66744	1.60E-14	3.97E-12	TAP1/PSMB8/PSMB9/HLA-E/TAP2/PSME1/B2M/PSME2/PSMB10/HLA-A/UBE2L6/HLA-C/HLA-B/PSMA4/PSMA1/HLA-F/DTX3L/PSMA6/RNF213/CTSS/UBB/PSMD11/PSMB7/FBXO6/TRIM21/ERAP2/FBXO7/UBR2/TRIM69/UBC/PSMA3/RNF114/CUL7/PSMA2/KCTD7/UBA6/PSMC4/CUL2/ITGB5/RBCK1/PSMA5/UBOX5/PSMB2/IKBKG/RNF19B/HERC6/TAPBP/PSMA7/UBA7/UBE2Z/PSMD3/KLHL22/SEC24C/PSMC1/PSMF1/PSMB4/ASB1/UBE2D3/ERAP1/PDIA3/PSMB1/VAMP3/MKRN1/KLHL5/FBXO2/ZNRF1/PSMB3/STX4/MYD88/FBXL5/PIK3C3/PSMD13/SEC31A/ITGAV/FBXO31/CDC27/PSMC6/RNF130/PSMB6/SOCS1/PSMD7/RNF34/CUL3/KLHL3/PSMD12/VHL/PSMD14/LMO7/PSMB5/RNF115/UBE2E3/FBXO21/TRIM11/HMGB1/FBXO10/PSMD1/UBE2M/SEC24A/KLHL21/ASB7/UBE3B/VAMP8/HERC1/FBXO4/UBE2Q1/PSME3/UBE2V2/ASB14/ATG14/PSMC2/HLA-G/RNF123/RNF217/RNF126/TRIP12/UBE2J2/SEC61B/PSMD2/TLR4/UBE2L3/HERC4/UBE2Q2/UBE2F/RNF6/LONRF1/PSMD6/DZIP3/HERC5/KEAP1/UBE2G1/PSMC3/CBLB/TRAF7/STUB1/CDC16/UBE2E1/RNF7/UBE2O/SOCS3/ASB8/ANAPC2/HECTD3/KLHL2/RBX1/FBXO41/RNF4/CALR/UBE2D2/RLIM/LRSAM1/SEC22B/UBE4A/FBXW7/RNF144B/IKBKB/SEC24B/LRRC41/PSMD5/HACE1/ASB6/UBE2N/PSMD9/RNF19A/LNPEP/FBXO44/KLHL13/UNKL/KCTD6/FBXL18/PSMD4/TRIM37/UBE2E2/SEC23A/UBE3D/CHUK/FBXO9/TRAIP/BLMH/NCF1/CYBB/DET1/FBXW2/S100A1/ANAPC10/ANAPC13/CDC34/RNF14/RNF138/TRIM32/FBXO22/SEC13/RCHY1/FBXW10/SAR1B/THOP1/FBXL20/SKP1/UBE2K/KBTBD8/FBXW11/HERC3/UBA3/ATG7/SPSB1/FBXL3/FBXO32/PSMC5/FBXL12/PSMD8/FBXW5/MYLIP/WSB1/UBR4/RNF41/UBA52/ZNRF2/TIRAP/UBA1/SKP2/KLHL25/ANAPC5/HSPA5/MIB2/MGRN1/BCAP31/CYBA/CCNF/FBXW9/RPS27A/ARIH2/UBA5/FBXL22/FBXL19/FBXL15/FBXL13/FBXL14/CDC26/SIAH1/FBXL8/RNF25
R-HSA-8941858	Regulation of RUNX3 expression and activity	51/ 4886	55/ 11146	0.92727	2.11531	7.32520	1.71E-14	3.97E-12	PSMB8/PSMB9/PSME1/PSME2/PSMB10/PSMA4/PSMA1/PSMA6/UBB/PSMD11/PSMB7/UBC/PSMA3/PSMA2/PSMC4/PSMA5/PSMB2/PSMA7/PSMD3/PSMC1/PSMF1/PSMB4/PSMB1/CDKN2A/TGFB1/PSMB3/MDM2/PSMD13/PSMC6/PSMB6/PSMD7/PSMD12/PSMD14/PSMB5/PSMD1/PSME3/PSMC2/PSMD2/PSMD6/PSMC3/PSMD5/PSMD9/PSMD4/SRC/PSMC5/CBFB/PSMD8/UBA52/RUNX3/RPS27A/EP300
R-HSA-9759194	Nuclear events mediated by NFE2L2	79/ 4886	97/ 11146	0.81443	1.85789	7.49700	2.50E-14	4.87E-12	PSMB8/PSMB9/PSME1/PSME2/PSMB10/PSMA4/PSMA1/PSMA6/UBB/PSMD11/PSMB7/UBC/PSMA3/PSMA2/PSMC4/PSMA5/PSMB2/PSMA7/GSTA1/PSMD3/PSMC1/PSMF1/PSMB4/CCL2/PSMB1/CDKN2A/AREG/PSMB3/RELA/MYC/PSMD13/PSMC6/BACH1/PSMB6/ABCC3/PSMD7/SRXN1/PSMD12/PSMD14/PSMB5/PSMD1/GCLM/SQSTM1/ME1/GCLC/PSME3/PSMC2/TXN/TXNRD1/PSMD2/SP1/PRKAA2/PSMD6/KEAP1/MAFG/PSMC3/TALDO1/RBX1/SLC7A11/ATF4/NQO1/PSMD5/PSMD9/PSMD4/PGD/NFKB1/ABCC1/ABCF2/GSR/SKP1/G6PD/PSMC5/PSMD8/UBA52/TKT/RPS27A/MAFK/NOTCH1/EP300
R-HSA-69206	G1/S Transition	100/ 4886	131/ 11146	0.76336	1.74138	7.54077	2.71E-14	4.87E-12	PSMB8/PSMB9/PSME1/PSME2/PSMB10/PSMA4/PSMA1/PSMA6/MAX/UBB/PSMD11/PSMB7/UBC/PSMA3/PSMA2/LIN52/PSMC4/PSMA5/E2F5/PSMB2/PSMA7/PSMD3/PSMC1/PSMF1/PSMB4/PSMB1/CDKN1A/CDK2/CABLES1/PSMB3/MYC/PPP2CA/PSMD13/PSMC6/WEE1/PSMB6/PSMD7/RPA1/RBBP4/PSMD12/PSMD14/PSMB5/GMNN/MCM7/PSMD1/RBL1/TYMS/RBL2/TFDP2/PPP2CB/CCNH/PSME3/PSMC2/MCM5/PSMD2/CDC25A/TK1/PSMD6/POLE2/MCM8/FBXO5/PSMC3/RB1/POLA1/POLE4/E2F4/E2F1/CDK7/PSMD5/CCND1/POLE/PSMD9/CDK4/PSMD4/ORC2/MCM10/LIN37/LIN9/MCM2/PTK6/CDKN1B/CDC45/CCNE2/TFDP1/ORC5/POLE3/MCM4/LIN54/SKP1/PSMC5/PSMD8/CDC6/UBA52/ORC4/SKP2/RRM2/RPS27A/CKS1B/CCNE1/MNAT1
R-HSA-9013694	Signaling by NOTCH4	69/ 4886	82/ 11146	0.84146	1.91956	7.38344	4.57E-14	6.99E-12	PSMB8/PSMB9/PSME1/PSME2/PSMB10/PSMA4/PSMA1/PSMA6/UBB/PSMD11/PSMB7/UBC/PSMA3/PSMA2/APH1B/SMAD3/PSMC4/PSMA5/PSMB2/PSMA7/PSMD3/PSMC1/PSMF1/PSMB4/PSMB1/PSENEN/PSMB3/PSMD13/PSMC6/PSMB6/PSMD7/SNW1/PSMD12/PSMD14/PSMB5/PSMD1/PSEN2/RBPJ/APH1A/PSME3/PSMC2/PSMD2/PSMD6/PSMC3/JAG1/RBX1/NOTCH4/ADAM10/MAML3/FBXW7/NCSTN/PSMD5/PSMD9/PSEN1/PSMD4/NOTCH2/KAT2B/YWHAZ/SKP1/DLL4/PSMC5/PSMD8/UBA52/MAML1/MAML2/HES1/RPS27A/NOTCH1/EP300
R-HSA-69242	S Phase	118/ 4886	162/ 11146	0.72840	1.66162	7.49407	4.74E-14	6.99E-12	PSMB8/PSMB9/PSME1/CDC25B/PSME2/PSMB10/PSMA4/PSMA1/PSMA6/MAX/UBB/PSMD11/PSMB7/UBC/PSMA3/PSMA2/LIN52/PSMC4/PSMA5/E2F5/PSMB2/PSMA7/PSMD3/PSMC1/PSMF1/PSMB4/PSMB1/CDKN1A/CDK2/CABLES1/PSMB3/MYC/PSMD13/CDC27/PSMC6/WEE1/PSMB6/PSMD7/RPA1/RBBP4/PSMD12/PSMD14/PSMB5/GMNN/MCM7/PSMD1/RBL2/TFDP2/CCNH/PSME3/PSMC2/MCM5/RFC2/ANAPC16/POLD1/PSMD2/CDC25A/PDS5B/PSMD6/POLE2/LIG1/MCM8/PSMC3/RB1/CDC16/UBE2E1/POLA1/ANAPC2/GINS2/RBX1/FEN1/POLE4/E2F4/E2F1/CDK7/PSMD5/CCND1/POLE/PSMD9/CDK4/PSMD4/ORC2/RFC5/LIN37/LIN9/GINS4/POLD3/MCM2/PTK6/CDKN1B/CDC45/ANAPC10/CCNE2/TFDP1/ORC5/POLE3/ESCO2/MCM4/CDCA5/LIN54/RFC4/SKP1/RFC3/PSMC5/PSMD8/CDC6/UBA52/ORC4/GINS3/SKP2/ANAPC5/POLD4/RPS27A/CKS1B/CDC26/SMC1A/CCNE1/MNAT1
R-HSA-450408	AUF1 (hnRNP D0) binds and destabilizes mRNA	51/ 4886	56/ 11146	0.91071	2.07753	7.14148	1.10E-13	1.49E-11	PSMB8/PSMB9/PSME1/PSME2/PSMB10/PSMA4/PSMA1/PSMA6/UBB/PSMD11/PSMB7/HSPB1/UBC/PSMA3/PSMA2/PSMC4/PSMA5/PSMB2/PSMA7/PSMD3/PSMC1/PSMF1/PSMB4/PSMB1/HSPA1A/PSMB3/PSMD13/PSMC6/PSMB6/PSMD7/PSMD12/PSMD14/PSMB5/PSMD1/EIF4G1/PSME3/PSMC2/HSPA1B/PSMD2/PSMD6/PSMC3/PSMD5/PSMD9/PSMD4/PABPC1/PSMC5/PSMD8/UBA52/HSPA8/RPS27A/HNRNPD
R-HSA-9020702	Interleukin-1 signaling	89/ 4886	115/ 11146	0.77391	1.76546	7.28942	1.75E-13	2.18E-11	PSMB8/NLRC5/PSMB9/PSME1/PSME2/PSMB10/PSMA4/PSMA1/PSMA6/UBB/PSMD11/PSMB7/CASP8/UBC/PSMA3/PSMA2/NOD1/ALPK1/SAA1/PSMC4/USP18/IL1R2/PSMA5/PSMB2/IKBKG/PSMA7/TRAF6/PSMD3/PSMC1/PSMF1/PSMB4/APP/PSMB1/RIPK2/PSMB3/MYD88/RELA/IL1RAP/PSMD13/PSMC6/PSMB6/PSMD7/IRAK1/PSMD12/PSMD14/MAP3K8/PSMB5/PELI3/HMGB1/PSMD1/NKIRAS1/SQSTM1/NLRX1/PSME3/PSMC2/MAP3K3/PSMD2/PSMD6/IL1RN/PSMC3/RBX1/IKBKB/PSMD5/UBE2N/PSMD9/NKIRAS2/PSMD4/NFKB1/CHUK/TRAF2/LRRC14/MAP2K4/IL1A/MAP2K6/USP14/SKP1/IL1B/FBXW11/PSMC5/PSMD8/TAB3/UBA52/TAB2/MAP3K7/RPS27A/TOLLIP/IKBIP/TAB1/PELI2
R-HSA-8878159	Transcriptional regulation by RUNX3	77/ 4886	96/ 11146	0.80208	1.82972	7.21301	2.53E-13	2.93E-11	PSMB8/PSMB9/PSME1/PSME2/PSMB10/PSMA4/PSMA1/PSMA6/UBB/PSMD11/PSMB7/UBC/PSMA3/PSMA2/SMAD3/PSMC4/TEAD2/PSMA5/PSMB2/PSMA7/PSMD3/PSMC1/PSMF1/PSMB4/PSMB1/CDKN1A/CDKN2A/TGFB1/PSMB3/BRD2/MDM2/MYC/PSMD13/PSMC6/PSMB6/PSMD7/SNW1/PSMD12/PSMD14/PSMB5/TCF7L1/PSMD1/RUNX1/RBPJ/YAP1/PSME3/FOXO3/PSMC2/PSMD2/PSMD6/PSMC3/ZFHX3/SMAD4/JAG1/MAML3/PSMD5/CCND1/TCF7L2/PSMD9/PSMD4/KAT2B/

									TEAD1/KRAS/SRC/BCL2L11/PSMC5/CBFB/PSMD8/UBA52/MAML1/RUNX3/TEAD4/MAML2/HES1/RPS27A/NOTCH1/EP300
R-HSA-9010553	Regulation of expression of SLITs and ROBOs	122/ 4886	172/ 11146	0.70930	1.61807	7.21686	4.14E-13	4.47E-11	PSMB8/PSMB9/PSME1/PSME2/PSMB10/PSMA4/PSMA1/PSMA6/UBB/PSMD11/PSMB7/UBC/PSMA3/PSMA2/PSMC4/CUL2/PSMA5/PSMB2/PSMA7/PSMD3/PSMC1/PSMF1/PSMB4/PSMB1/MSI1/PSMB3/RPL3/PSMD13/PSMC6/RPS6/PSMB6/PSMD7/PSMD12/ETF1/PSMD14/RPL10A/PSMB5/RPL13A/RPL5/RPS3A/USP33/PSMD1/EIF4G1/RPL15/RPL7/RPL23/RPL31/RPL12/DAG1/UPF2/RPS3/PSME3/PSMC2/NCBP2/PSMD2/RPS4X/RPL26/RPL34/RPL7A/PSMD6/RPL6/PSMC3/RPS27/LDB1/RPL22/RPL30/RPL4/EIF4A3/RPL11/RPS9/RBX1/RPS15A/RPS7/RPS14/RPS8/RPL14/RPL10/RPLP0/PSMD5/GSPT2/RPL37A/PSMD9/RPL41/UPF3A/PSMD4/RPL39/ROBO3/RPL9/PABPC1/RPL35A/RPL8/RPL32/RPL36AL/RPL26L1/RPS23/RPS28/RPS15/RPL37/RPLP1/RPL24/RPS25/MAGOH/RPL13/RPS24/RPL18/RPL36/RPS10/RBM8A/LHX4/PSMC5/PSMD8/UBA52/RNPS1/HOXA2/RPS16/RPL27A/RPS5/FAU/RPS2/RPS27A/RPL29/RPL38
R-HSA-9604323	Negative regulation of NOTCH4 signaling	49/ 4886	54/ 11146	0.90741	2.06999	6.96307	4.81E-13	4.87E-11	PSMB8/PSMB9/PSME1/PSME2/PSMB10/PSMA4/PSMA1/PSMA6/UBB/PSMD11/PSMB7/UBC/PSMA3/PSMA2/PSMC4/PSMA5/PSMB2/PSMA7/PSMD3/PSMC1/PSMF1/PSMB4/PSMB1/PSMB3/PSMD13/PSMC6/PSMB6/PSMD7/PSMD12/PSMD14/PSMB5/PSMD1/PSME3/PSMC2/PSMD2/PSMD6/PSMC3/RBX1/NOTCH4/FBXW7/PSMD5/PSMD9/PSMD4/YWHAZ/SKP1/PSMC5/PSMD8/UBA52/RPS27A
R-HSA-376176	Signaling by ROBO receptors	148/ 4886	219/ 11146	0.67580	1.54164	7.15175	7.28E-13	6.94E-11	PSMB8/PSMB9/PSME1/PSME2/PSMB10/PSMA4/PSMA1/PSMA6/UBB/PSMD11/PSMB7/SRGAP2/UBC/PSMA3/PSMA2/PSMC4/CUL2/PSMA5/PSMB2/PSMA7/PSMD3/PSMC1/PSMF1/PSMB4/GPC1/PSMB1/MSI1/PSMB3/RPL3/FLRT3/PSMD13/PSMC6/RPS6/PSMB6/PSMD7/PSMD12/PRKACB/ETF1/PSMD14/RPL10A/PSMB5/RPL13A/RPL5/RPS3A/USP33/PSMD1/EIF4G1/NELL2/RPL15/RPL7/RPL23/RPL31/CXCR4/ENAH/RPL12/DAG1/UPF2/SOS1/RPS3/PAK1/PSME3/AKAP5/PSMC2/NCBP2/PAK6/PSMD2/RPS4X/PFN2/RPL26/SRGAP1/RPL34/RPL7A/PSMD6/RPL6/PFN1/PSMC3/RPS27/LDB1/RPL22/RPL30/RPL4/EIF4A3/RPL11/RPS9/RBX1/RPS15A/RPS7/RPS14/PAK2/RPS8/RPL14/RPL10/DCC/RPLP0/PSMD5/GSPT2/RPL37A/PSMD9/RPL41/EVL/UPF3A/PSMD4/RPL39/ROBO3/CAP2/RPL9/PABPC1/RPL35A/RPL8/RPL32/RPL36AL/RPL26L1/RPS23/RPS28/RPS15/RPL37/RPLP1/RPL24/RPS25/MAGOH/RPL13/RPS24/RPL18/SRC/PRKCA/RPL36/CAP1/RPS10/RBM8A/LHX4/PSMC5/PSMD8/UBA52/ABL2/RNPS1/HOXA2/RPS16/RPL27A/RPS5/VASP/NRP1/FAU/PPP3CB/RPS2/RPS27A/RPL29/PRKACA/RPL38
R-HSA-109581	Apoptosis	126/ 4886	180/ 11146	0.70000	1.59685	7.13191	7.95E-13	7.16E-11	PSMB8/PSMB9/PSME1/PSME2/PSMB10/BAK1/PSMA4/PSMA1/PSMA6/GSDMD/UBB/PSMD11/PSMB7/PMAIP1/CASP8/UBC/PSMA3/TNFSF10/DYNLL2/PSMA2/CASP7/PSMC4/DAPK1/STAT3/PSMA5/PSMB2/TICAM1/PSMA7/CASP3/PSMD3/FAS/PSMC1/PSMF1/PSMB4/GSN/PPP3CC/PSMB1/TP63/CDKN2A/NMT1/PSMB3/AVEN/CYCS/PSMD13/PSMC6/PSMB6/PSMD7/STK24/RIPK1/PSMD12/UACA/PSMD14/PSMB5/CFLAR/HMGB1/PSMD1/PTK2/MAGED1/PLEC/YWHAB/CASP6/ARHGAP10/BMF/TFDP2/BAD/APPL1/DAPK2/TJP2/PSME3/C1QBP/YWHAQ/PSMC2/PPP3R1/DNM1L/KPNA1/CASP9/PSMD2/TLR4/SATB1/BID/PSMD6/CARD8/CLSPN/PSMC3/BIRC2/OCLN/PAK2/E2F1/KPNB1/DCC/TP73/PSMD5/HMGB2/DAPK3/FADD/PSMD9/PSMD4/UNC5B/DIABLO/TRAF2/LMNB1/YWHAZ/XIAP/FNTA/SFN/TFDP1/TNFRSF10B/BBC3/DBNL/ROCK1/BCL2L11/ADD1/YWHAG/OMA1/PSMC5/PSMD8/UNC5A/MAPK8/UBA52/PPP1R13B/OPA1/PRKCQ/BCAP31/RPS27A/APIP/DFFB
R-HSA-1168372	Downstream signaling events of B Cell Receptor (BCR)	68/ 4886	83/ 11146	0.81928	1.86894	7.01979	9.38E-13	8.00E-11	PSMB8/PSMB9/PSME1/PSME2/PSMB10/PSMA4/PSMA1/PSMA6/UBB/RASGRP3/PSMD11/PSMB7/CALM3/UBC/PSMA3/PSMA2/PSMC4/PSMA5/PSMB2/IKBKG/PSMA7/PSMD3/PSMC1/PSMF1/PSMB4/PSMB1/NFKBIE/PSMB3/RELA/PSMD13/PSMC6/PSMB6/PSMD7/PSMD12/PSMD14/PSMB5/NFATC2/PSMD1/BCL10/PSME3/PSMC2/PPP3R1/PSMD2/RASGRP1/PSMD6/PSMC3/NFATC1/IKBKB/PSMD5/MALT1/PSMD9/PSMD4/NFKB1/CHUK/PPP3CA/KRAS/CALM1/SKP1/FBXW11/REL/PSMC5/FKBP1A/PSMD8/HRAS/UBA52/PPP3CB/MAP3K7/RPS27A
R-HSA-169911	Regulation of Apoptosis	48/ 4886	53/ 11146	0.90566	2.06600	6.87229	1.00E-12	8.11E-11	PSMB8/PSMB9/PSME1/PSME2/PSMB10/PSMA4/PSMA1/PSMA6/UBB/PSMD11/PSMB7/UBC/PSMA3/PSMA2/PSMC4/PSMA5/PSMB2/PSMA7/PSMD3/PSMC1/PSMF1/PSMB4/PSMB1/PSMB3/PSMD13/PSMC6/PSMB6/PSMD7/PSMD12/PSMD14/PSMB5/PSMD1/ARHGAP10/PSME3/PSMC2/PSMD2/PSMD6/PSMC3/PAK2/PSMD5/PSMD9/PSMD4/OMA1/PSMC5/PSMD8/UBA52/OPA1/RPS27A

(D) KEGG pathway analysis

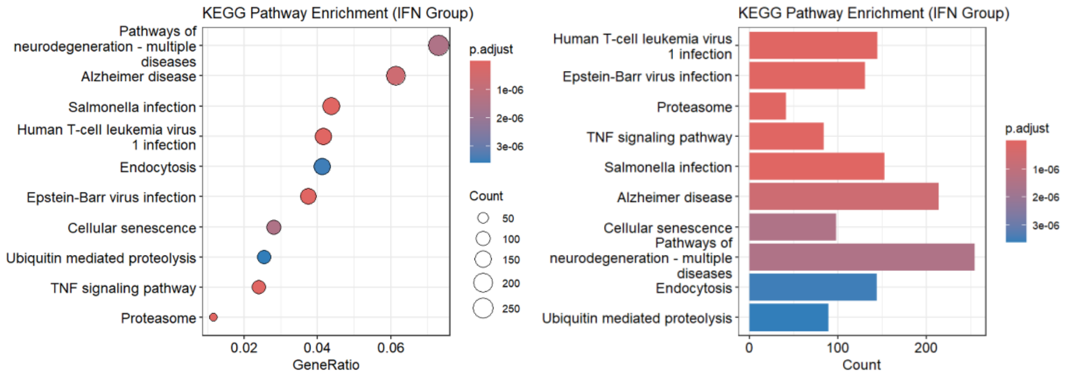

Top 20 KEGG pathway

category	Sub-category	ID	Description	Gene Ratio	Bg Ratio	Rich actor	Fold Erichment	z-score	p-value	p. adjust	q-value	geneID
Human Diseases	Infectious disease: viral	hsa05166	Human T-cell leukemia virus 1 infection	145/3489	224/8538	0.64732	1.58407	7.36354	2.53E-13	8.73E-11	5.56E-11	HLA-E/B2M/IL15RA/HLA-A/HLA-C/HLA-DMA/HLA-B/HLA-F/ICAM1/HLA-DRA/HLA-DPB1/IL15/HLA-DPA1/HLA-DOB/TGFBR1/HLA-DMB/STAT5B/SMAD3/HLA-DRB1/IL1R2/IKBKG/PPP3CC/CDKN1A/CDK2/CDKN2A/CREB3L4/TGFB1/ETS2/ADCY3/TCF3/RELA/MYC/CD40/VDAC2/ITGB2/LCK/CCND3/ELK4/CDC27/PRKACB/CHEK1/NFYB/CCND2/HLA-DQB1/E2F3/NFATC2/CREB3L1/MAP2K2/JUN/PIK3CD/TNF/HLA-G/PPP3R1/MAPK9/HLA-DOA/TGFB3/MAD1L1/ANAPC16/TGFB2/MAP3K3/ZFP36/MAP3K14/ATF6B/GPS2/STAT5A/ETS1/LTBR/CREB1/PIK3R3/NFATC4/SLC25A5/PIK3CB/RB1/NFATC1/CDC16/SMAD4/POLB/ANAPC2/SLC25A4/CALR/BUB1B/HLA-DRB5/VDAC1/E2F1/ADCY6/TNFRSF1A/ATF4/CREB3/SLC2A1/IKBKB/PIK3R1/CCND1/ATM/CREB3L3/FOSL1/CDK4/TLN2/MAP3K1/HLA-DQA1/NFKB1/CHUK/JAK1/KAT2B/PPP3CA/IL2RG/XIAP/KAT5/ATR/ADCY4/ANAPC10/MAP2K4/ANAPC13/CCNE2/KRAS/CSF2/MAD2L1/CREB5/VDAC3/E2F2/CRTC2/DLG1/CDKN2C/CRTC1/TBP/CHEK2/FOS/MAPK8/HRAS/CRTC3/ESPL1/ADCY9/ANAPC5/BUB3/ADCY1/HLA-DQB2/NRP1/PPP3CB/CDKN2B/HLA-DQA2/PRKACA/TRRAP/CDC26/EP300/TBPL1/CCNE1
Human Diseases	Infectious disease: viral	hsa05169	Epstein-Barr virus infection	131/3489	204/8538	0.64216	1.57143	6.86681	8.64E-12	1.28E-09	8.17E-10	TAP1/HLA-E/TAP2/B2M/STAT1/HLA-A/HLA-C/HLA-DMA/HLA-B/BAK1/OAS2/HLA-F/ICAM1/OAS3/IRF9/HLA-DRA/ISG15/STAT2/HLA-DPB1/LYN/PSMD11/CASP8/HLA-DPA1/CIR1/HLA-DOB/HLA-DMB/EIF2AK2/PSMC4/HLA-DRB1/STAT3/IKBKG/TAPBP/TRAF6/CASP3/PSMD3/FAS/PSMC1/PDIA3/NFKBIE/CDKN1A/CDK2/MAP2K3/TBK1/CYCS/TRAF5/MYD88/MDM2/RELA/MYC/CD40/PSMD13/CCND3/PSMC6/PSMD7/IRAK1/RIPK1/SNW1/PSMD12/PSMD14/SIN3A/CCND2/HLA-DQB1/E2F3/ADRM1/PSMD1/ENTPD1/IFNAR1/RBPJ/CXCL10/JUN/PIK3CD/TNF/PSMC2/HLA-G/MAPK9/HLA-DOA/CD58/IFNAR2/CASP9/PSMD2/OAS1/MAP3K14/BID/PSMD6/PSMC3/PIK3R3/TNFAIP3/CD44/PIK3CB/RB1/CALR/HLA-DRB5/MAPK11/E2F1/HDAC2/IKBKB/IRF3/PIK3R1/CCND1/FADD/CDK4/PSMD4/HLA-DQA1/DDB2/NFKB1/IRF7/CHUK/GADD45A/JAK1/TRAF2/CDKN1B/MAP2K4/MAP2K6/CCNE2/SAP30L/E2F2/BCL2L11/PSMC5/PSMD8/MAPK8/SKP2/RUNX3/TAB2/HES1/HLA-DQB2/MAVS/MAP3K7/HLA-DQA2/TAB1/MAP2K7/CCNE1
Genetic Information Processing	Folding, sorting and degradation	hsa03050	Proteasome	41/3489	46/8538	0.89130	2.18113	6.67685	1.11E-11	1.28E-09	8.17E-10	PSMB8/PSMB9/PSME1/PSME2/PSMB10/PSMA4/PSMA1/PSMA6/PSMD11/PSMB7/PSMA3/POMP/PSMA2/PSMC4/PSMA5/PSMB2/PSMA7/PSMD3/PSMC1/PSMF1/PSMB4/PSMB1/PSMB3/PSMD13/PSMC6/PSMB6/PSMD7/PSMD12/PSMD14/PSMB5/ADRM1/PSMD1/PSME3/PSMC2/PSMD2/PSMD6/PSMC3/PSMD9/PSMD4/PSMC5/PSMD8
Environmental Information Processing	Signal transduction	hsa04668	TNF signaling pathway	84/3489	119/8538	0.70588	1.72738	6.64208	3.88E-11	3.35E-09	2.14E-09	IRF1/ICAM1/CASP10/CX3CL1/IL15/CASP8/CYLD/BIRC3/FRMD8/TNFRSF1B/CSF1/CASP7/MLKL/IKBKG/CASP3/FAS/CCL2/CXCL2/DAB2IP/MAP2K3/CREB3L4/TRAF5/RPS6KA4/RELA/CCL5/IL18R1/RIPK1/MAP3K8/CFLAR/RHBDF2/CREB3L1/CXCL10/JUN/PIK3CD/TNF/CCL20/DNM1L/MAPK9/CXCL3/RPS6KA5/MAP3K14/ATF6B/CEBPB/CREB1/PIK3R3/TNFAIP3/PIK3CB/PTGS2/MAP3K5/CXCL6/SOCS3/BIRC2/JAG1/MAPK11/TNFRSF1A/ATF4/CREB3/IKBKB/PIK3R1/FADD/CREB3L3/NFKB1/CHUK/TRAF2/XIAP/MAP2K4/MAP2K6/CSF2/CREB5/IL1B/RIPK3/JUNB/MMP14/FOS/MAPK8/TAB3/EDN1/TAB2/RHBDF1/ADAM17/PGAM5/MAP3K7/TAB1/MAP2K7
Human Diseases	Infectious disease: bacterial	hsa05132	Salmonella infection	153/3489	251/8538	0.60956	1.49167	6.57223	6.03E-11	4.16E-09	2.65E-09	CASP1/BAK1/GSDMD/CASP8/TNFSF10/TUBA1A/DYNLL2/BIRC3/NOD1/CASP7/MLKL/IKBKG/BRK1/TRAF6/CASP3/MAP2K3/EXOC2/RIPK2/VPS33A/CYCS/DYNLT1/MYD88/RELA/MYC/PIK3C3/ARF1/PKN1/IRAK1/RIPK1/MYL12A/TUBA1C/MYO6/DYNC2H1/MYL12B/RALA/ACTR10/ACTR3/VPS18/VPS11/ARPC5L/DYNC1LI1/TCF7L1/KPNA4/RAB5C/ACTR1B/TUBB2A/CASP5/DCTN6/MAP2K2/TLR5/RPS3/JUN/PIK3CD/TNF/ELMO2/ARL8A/PAK1/DYNC1H1/DYNLRB1/RILP/MAPK9/TXN/KPNA1/S100A10/KLC1/TLR4/ABI1/PFN2/KPNA3/RHOG/PFN1/PIK3CB/MYL9/VPS41/BIRC2/ARPC3/SNX9/FLNA/WASL/CYFIP2/CASP4/DYNC1LI2/MAPK11/DYNC2LI1/TNFRSF1A/DCTN4/KLC2/IL18/IKBKB/TCF7L2/TUBB6/FADD/CYTH4/NAIP/TUBB4B/AHNAK2/ACBD3/NCKAP1/M6PR/NFKB1/FHOD1/TUBB/CHUK/TRAF2/RAB9A/RAB5A/DYNLT3/VPS39/ARHGEF26/ACTB/EXOC7/RAB7A/STX10/MAP2K4/MAP2K6/TNFRSF10B/ARF6/SNX33/PODXL/FBXO22/ACTR2/TUBB3/SKP1/IL1B/KLC4/TXN2/RIPK3/AHNAK/CYTH3/HSP90AA1/FOS/ARL8B/MAPK8/TAB3/HRAS/TIRAP/NLRC4/DCTN3/PLEKHM2/FYCO1/TAB2/MYL5/KIF5B/TUBAL3/MAP3K7/ACTR1A/EXOC4/MYL2/ACTR3C/DYNC1I2/TAB1/WASF3/MAP2K7
Human Diseases	Neurodegenerative disease	hsa05010	Alzheimer disease	214/3489	391/8538	0.54731	1.33934	5.70992	1.06E-08	6.07E-07	3.87E-07	PSMA4/PSMA1/PSMA6/ATP2A3/ATG13/NDUFA9/PSMD11/PSMB7/CALM3/CASP8/PSMA3/TUBA1A/PSMA2/APH1B/EIF2AK2/CSF1/CASP7/IRS1/PSMC4/NOS2/NDUFS2/PSMA5/PSMB2/IKBKG/PSMA7/CSNK1E/CASP3/PSMD3/FAS/PSMC1/PSMB4/PPP3CC/APP/DVL2/PSMB1/UQCRFS1/CAPN1/SLC39A1/PSENEN/PSMB3/CYCS/NDUFAB1/ULK2/RELA/VDAC2/PIK3C3/PSMD13/CACNA1D/SDHA/PSMC6/PSMB6/LRP1/CYC1/APBB1/PSMD7/DKK4/TUBA1C/PSMD12/FZD7/WNT7A/NDUFV2/PSMD14/PSMB5/BACE1/WNT4/ADRM1/PSMD1/SDHB/SLC39A11/PSEN2/CALML4/NDUFS3/BACE2/TUBB2A/INSR/FZD3/NDUFS6/NDUFV1/APH1A/MAP2K2/BAD/PIK3CD/NDUFB5/TNF/SLC39A14/PLCB4/ATG14/NDUFS7/UQCRC1/PSMC2/SLC39A13/PPP3R1/MAPK9/COX5A/NDUFB3/ULK1/CASP9/PSMD2/KLC1/FZD6/BID/DVL1/NDUFB7/CSNK2A1/SLC11A2/LRP6/PSMD6/UQCRC2/SLC39A10/CSNK1A1/WNT7B/PSMC3/PIK3R3/EIF2AK3/SLC25A5/PIK3CB/PTGS2/DDIT3/MAP3K5/EIF2S1/NDUFS1/COX8A/SLC25A4/NDUFB10/ADAM10/NDUFA10/WNT3/VDAC1/COX7B/TNFRSF1A/ATF4/KLC2/AXIN2/IKBKB/NCSTN/PIK3R1/PLCB3/TUBB6/FADD/PSMD9/NDUFB9/PSEN1/ITPR2/NDUFC2/TUBB4B/HSD17B10/NRBF2/PSMD4/XBP1/NFKB1/TUBB/CHUK/WNT9

											A/COX7A2L/NDUFA1/TRAF2/PPP3CA/CYBB/SNCA/NDUFB8/SDHC/MTOR/AMBRA1/IL1A/ERN1/SDHD/NDUFS8/KRAS/CALM1/ATG2A/GRIN2D/TUBB3/VDAC3/NDUFA8/GRIN1/APC2/NDUFB4/IL1B/CSNK2B/WNT10A/KLC4/IRS2/WNT11/PSMC5/PSMD8/ARAF/FZD4/MAPK8/ITPR3/HRAS/ATF6/MME/ITPR1/UQCRH/PPIF/COX4I1/FZD5/APOE/ATG2B/ADAM17/KIF5B/CAPN2/PPP3CB/TUBAL3/IDE/NDUFA5/NDUFV3/SLC39A5/ATP2A2/FZD2/COX7C/MAP2K7/SLC39A3/UQCR10
Cellular Processes	Cell growth and death	hsa04218	Cellular senescence	98/3489	157/8538	0.62420	1.52750	5.54531	3.21E-08	1.48E-06	9.42E-07	HLA-E/HLA-A/HLA-C/HLA-B/HLA-F/CALM3/TGFBR1/LIN52/SMAD3/NBN/E2F5/PPP3CC/CDKN1A/CDK2/MAP2K3/CDKN2A/CAPN1/TGFB1/MDM2/RELA/MYC/VDAC2/CACNA1D/CCND3/RBBP4/RAD9A/CHEK1/TRPV4/CCND2/E2F3/NFATC2/CALML4/SQSTM1/RBL1/MAP2K2/RBL2/PIK3CD/FOXO3/HLA-G/PPP3R1/TGFB3/ZFP36L1/TGFB2/TRAF3IP2/CDC25A/ETS1/TRPM7/PIK3R3/NFATC4/SLC25A5/PIK3CB/RB1/NFATC1/SLC25A4/MAPKAPK2/TSC2/MAPK11/E2F4/MRAS/VDAC1/E2F1/TSC1/PIK3R1/RAD1/CCND1/ATM/MYBL2/ITPR2/CDK4/NFKB1/FOXO1/LIN37/LIN9/GADD45A/PPP3CA/MTOR/ATR/IL1A/MAP2K6/CCNE2/KRAS/CALM1/VDAC3/LIN54/E2F2/FBXW11/SIRT1/CHEK2/RAD50/ITPR3/HRAS/PPP1CC/ITPR1/CAPN2/IGFBP3/PPP3CB/CDKN2B/CCNE1
Human Diseases	Neurodegenerative disease	hsa05022	Pathways of neurodegeneration - multiple diseases	255/3489	483/8538	0.52795	1.29196	5.49112	3.42E-08	1.48E-06	9.42E-07	UBE2L6/BAK1/PSMA4/PSMA1/PSMA6/ATP2A3/ATG13/NDUFA9/UBB/PSMD11/PSMB7/CALM3/CASP8/OPTN/UBC/PSMA3/TUBA1A/PSMA2/TNFRSF1B/CSF1/CASP7/PSMC4/NOS2/NDUFS2/MFN2/PSMA5/PSMB2/SOD1/PSMA7/PRKCG/CSNK1E/UBA7/CASP3/PSMD3/FAS/PSMC1/PSMB4/PPP3CC/APP/HIP1/SPG11/DVL2/PSMB1/UQCRFS1/MAP2K3/TBK1/CAPN1/PLCG1/PSMB3/CYCS/NDUFAB1/FIG4/ULK2/RELA/VDAC2/PIK3C3/PSMD13/CACNA1D/SDHA/PSMC6/PSMB6/CYC1/PSMD7/DKK4/TUBA1C/TOMM40L/DNAL4/GPX8/PSMD12/CAT/FZD7/HTT/WNT7A/NDUFV2/PSMD14/ACTR10/PSMB5/WNT4/DERL1/TRAP1/IFT57/ADRM1/GPX2/PSMD1/SDHB/DNALI1/PSEN2/ACTR1B/ATXN2L/CALML4/DNAH12/NDUFS3/SQSTM1/TUBB2A/FZD3/DCTN6/NDUFS6/NDUFV1/MAP2K2/BAD/GPX3/DNAL1/NDUFB5/TNF/PLCB4/MAP3K10/ATG14/NDUFS7/UQCRC1/PSMC2/PPP3R1/MAPK9/COX5A/NDUFB3/ULK1/UBE2J2/STX1A/CASP9/PSMD2/KLC1/UBE2L3/FZD6/BID/DVL1/NDUFB7/SMCR8/CSNK2A1/LRP6/PSMD6/UQCRC2/SPTBN2/DNAH6/CSNK1A1/WNT7B/UBE2G1/PSMC3/EIF2AK3/SLC25A5/PTGS2/DDIT3/DNAH5/MAP3K5/EIF2S1/ALS2/NDUFS1/COX8A/RAB8A/SLC25A4/NDUFB10/NDUFA10/TANK/WNT3/MAPK11/VDAC1/DNAH2/COX7B/TNFRSF1A/ATF4/DCTN4/KLC2/AXIN2/DNAH11/PLCB3/VCP/TUBB6/FADD/PSMD9/NDUFB9/MAP1LC3B/PSEN1/ATXN3/ITPR2/NDUFC2/TUBB4B/HSD17B10/NRBF2/PSMD4/XBP1/NFKB1/TUBB/WNT9A/COX7A2L/NDUFA1/RAB1A/TRAF2/PPP3CA/CYBB/CAMK2G/SNCA/RAB5A/NDUFB8/SDHC/MTOR/AMBRA1/IL1A/MAP2K6/ERN1/SDHD/NDUFS8/C9orf72/KRAS/CALM1/ATG2A/PRNP/GRIN2D/CAMK2D/MFN1/TUBB3/VDAC3/NDUFA8/GRIN1/APC2/NDUFB4/IL1B/GPX1/CSNK2B/PRKCA/WNT10A/KLC4/WNT11/PSMC5/PSMD8/ARAF/FZD4/MAPK8/ITPR3/HRAS/UBA52/UBA1/ATF6/CCS/TOMM40/DCTN3/HSPA5/WDR41/ITPR1/UQCRH/PPIF/COX4I1/FZD5/ATG2B/KIF5B/CAPN2/PPP3CB/TUBAL3/ACTR1A/DNAH9/RPS27A/NDUFA5/NDUFV3/ATP2A2/FZD2/COX7C/MAP2K7/UQCR10
Cellular Processes	Transport and catabolism	hsa04144	Endocytosis	144/3489	252/8538	0.57143	1.39835	5.33577	9.12E-08	3.50E-06	2.23E-06	HLA-E/HLA-A/HLA-C/HLA-B/HLA-F/GIT2/RAB31/PML/ARF3/IGF1R/TGFBR1/IQSEC1/SNX6/SMAD3/ARRB1/AP2A1/EPN3/DNM3/TRAF6/FGFR2/EHD2/FGFR3/VPS37A/PIP5K1B/ARAP3/DNM1/HSPA1A/MDM2/RAB10/TSG101/FGFR4/TFRC/ARF1/VPS4A/FOLR1/PSD3/ARFGAP1/SNX2/GIT1/AGAP2/ACTR3/SH3GLB1/SH3KBP1/CHMP5/ARPC5L/SMAP2/SNX3/PARD6G/ARFGAP3/EHD1/RAB5C/RAB11B/SNX32/CXCR4/AP2A2/AGAP1/RAB11FIP3/ARF5/VPS37B/PARD6A/HLA-G/HGS/PLD2/ARAP2/RAB35/HSPA1B/SNX1/CAV1/CHMP1A/HSPA1L/CLTB/VPS4B/STAM/EPS15/CLTA/GRK5/GRK6/LDLRAP1/CLTC/PARD6B/CBLB/CAPZA1/ARFGAP2/ARPC3/WASL/RAB8A/CHMP4B/IST1/STAM2/SH3GLB2/CYTH4/RAB11FIP2/ASAP2/ACAP2/EPS15L1/SNX12/RAB5A/IL2RG/ZFYVE9/RAB7A/CHMP4A/CAV2/BIN1/CHMP3/ARF6/ARRB2/CHMP4C/SNX5/VTA1/WIPF2/ASAP1/ACTR2/CBL/PIP5K1C/PRKCZ/VPS26A/VPS35/USP8/SRC/ARFGEF1/CYTH3/EHD4/VPS45/VPS28/HRAS/RNF41/ACAP1/PDCD6IP/CLTCL1/RAB11FIP5/PARD3/CHMP6/HSPA8/DAB2/GBF1/CHMP7/EPN1/KIF5B/AGAP3/ACTR3C/ASAP3/WIPF3/CHMP2A/ZFYVE27
Genetic Information Processing	Folding, sorting and degradation	hsa04120	Ubiquitin mediated proteolysis	89/3489	142/8538	0.62676	1.53376	5.33154	1.04E-07	3.60E-06	2.29E-06	UBE2L6/UBB/PML/RHOBTB2/UBC/BIRC3/CUL7/UBA6/CUL2/UBOX5/UBA7/TRAF6/UBE2Z/PIAS3/UBE2D3/MID1/FBXO2/NHLRC1/MDM2/CDC27/SOCS1/CUL3/WWP2/VHL/UBE2E3/SYVN1/UBE2M/UBE3B/HERC1/FBXO4/UBE2Q1/TRIP12/UBE2J2/ANAPC16/UBE2L3/HERC4/UBE2Q2/UBE2F/RHOBTB1/KEAP1/UBE2G1/CBLB/UBE4B/STUB1/CDC16/UBE2E1/RNF7/UBE2O/SOCS3/ANAPC2/BIRC2/RBX1/SAE1/UBE2D2/UBE4A/FBXW7/UBE2N/FANCL/KLHL13/TRIM37/MAP3K1/DDB2/UBE2E2/PIAS4/DET1/XIAP/ANAPC10/ANAPC13/CDC34/TRIM32/RCHY1/CBL/SKP1/BIRC6/UBE2K/FBXW11/HERC3/UBA3/UBA2/UBA52/UBA1/SKP2/ANAPC5/DDB1/MGRN1/RPS27A/PIAS2/CDC26/SIAH1
Genetic Information Processing	Folding, sorting and degradation	hsa04141	Protein processing in endoplasmic reticulum	103/3489	170/8538	0.60588	1.48267	5.28398	1.30E-07	4.08E-06	2.60E-06	BAK1/FBXO6/DNAJA1/DNAJA2/BAG1/SIL1/NPLOC4/DNAJC1/SEC24C/UBE2D3/PDIA3/CAPN1/FBXO2/HSPA1A/YOD1/UBXN4/PREB/SVIP/SEC31A/RAD23B/LMAN2/DERL1/UBQLN1/SYVN1/PLAA/SSR4/SEC24A/PRKCSH/P4HB/SSR2/DNAJB11/MAPK9/NGLY1/UBE2J2/HSPA1B/SEC61B/NSFL1C/EDEM2/ATF6B/GANAB/HSPA1L/SEC23B/UBQLN2/UBXN6/UBE2G1/MBTPS1/SSR1/UBE4B/EIF2AK3/DNAJC5/STUB1/DDIT3/RRBP1/MAP3K5/DERL2/DNAJB2/EIF2S1/SSR3/RBX1/CALR/UBE2D2/ATF4/BAG2/SEC24B/MAN1B1/VCP/HYOU1/ATXN3/UGGT2/XBP1/SEC23A/DNAJB12/HSPBP1/TRAF2/SEL1L/DNAJC3/MAGT1/CKAP4/ERN1/SEC63/SAR1A/ERP29/SEC13/SAR1B/SKP1/TRAM1/DAD1/DERL3/UBXN2A/HSP90AA1/TXNDC5/MAPK8/PPP1R15A/ATF6/HSPA5/UBQLN4/HSPA8/UGGT1/CAPN2/BCAP31/OSTC/MAP2K7/MBTPS2
Human Diseases	Infectious disease: parasitic	hsa05145	Toxoplasmosis	73/3489	112/8538	0.65179	1.59500	5.26883	1.51E-07	4.34E-06	2.77E-06	STAT1/HLA-DMA/HLA-DRA/JAK2/HLA-DPB1/CASP8/HLA-DPA1/BIRC3/HLA-DOB/HLA-DMB/NOS2/HLA-DRB1/STAT3/IKBKG/TRAF6/CASP3/LAMC2/MAP2K3/CIITA/LAMB2/HSPA1A/TGFB1/CYCS/MYD88/RELA/CD40/SOCS1/IRAK1/ALOX5/HLA-DQB1/GNAI2/BAD/LAMC1/TNF/MAPK9/HLA-DOA/TGFB3/HSPA1B/CASP9/TGFB2/TLR4/IFNGR2/IFNGR1/HSPA1L/LAMA5/ITGA6/BIRC2/HLA-DRB5/MAPK11/TNFRSF1A/LAMB3/IKBKB/GNAI3/HLA-DQA1/ITGB1/NFKB1/CHUK/JAK1/LAMA1/XIAP/MAP2K6/IL12A/MAPK8/LAMA4/TAB2/HSPA8/PPIF/HLA-DQB2/MAP3K7/LAMA3/HLA-DQA2/TAB1/PDPK1
Human Diseases	Infectious disease: viral	hsa05161	Hepatitis B	98/3489	163/8538	0.60123	1.47127	5.04982	4.39E-07	1.05E-05	6.68E-06	STAT1/CASP10/JAK2/STAT2/IFIH1/CASP8/TGFBR1/STAT5B/SMAD3/STAT3/IKBKG/TICAM1/PRKCG/TRAF6/CASP3/FAS/TLR3/CDKN1A/CDK2/MAP2K3/TBK1/CREB3L4/TGFB1/CYCS/MYD88/RELA/MYC/IRAK1/ATP6AP1/HSPG2/STAT6/E2F3/NFATC2/IFNAR1/YWHAB/CREB3L1/MAP2K2/SOS1/BAD/JUN/PIK3CD/TNF/YWHAQ/MAPK9/TGFB3/CASP9/TGFB2/TLR4/ATF6B/BID/PTK2B/STAT5A/CREB1/PIK3R3/NFATC4/PIK3CB/RB1/NFATC1/SMAD4/MAPK11/E2F1/ATF4/CREB3/IKBKB/IRF3/PIK3R1/FADD/CREB3L3/MAP3K1/DDB2/NFKB1/IRF7/CHUK/JAK1/YWHAZ/MAP2K4/MAP2K6/CCNE2/KRAS/CREB5/VDAC3/E2F2/SRC/P

												RKCA/ARAF/FOS/MAPK8/HRAS/TIRAP/TAB2/DDB1/MAVS/MAP3K7/BIRC5/TAB1/EP300/MAP2K7/CCNE1
Cellular Processes	Transport and catabolism	hsa04140	Autophagy - animal	101/3489	169/8538	0.59763	1.46248	5.04777	4.41E-07	1.05E-05	6.68E-06	ATG13/UBB/OPTN/IGF1R/WDR45/UBC/IRS1/DAPK1/CALCOCO2/TRAF6/YKT6/RRAGC/TBK1/RRAGB/GABARAP/ATG3/VPS33A/ULK2/PIK3C3/PPP2CA/MTMR14/PRKACB/STX17/IGBP1/CFLAR/VPS18/SH3GLB1/HMGB1/VPS11/TAX1BP1/SQSTM1/RRAGD/VAMP8/MAP2K2/ATG16L1/BAD/PIK3CD/DAPK2/PPP2CB/ATG14/ATG4A/MAPK9/ULK1/AKT1S1/PRKAA1/WDFY3/SMCR8/PRKAA2/CAMKK2/PIK3R3/EIF2AK3/PIK3CB/ATG12/VPS41/EIF2S1/RAB8A/UVRAG/LAMP2/TSC2/TANK/MRAS/HIF1A/TSC1/PIK3R1/DAPK3/VMP1/MAP1LC3B/RAB33B/NRBF2/RPS6KB2/GORASP1/RAB1A/MTOR/VPS39/EIF2AK4/RAB7A/AMBRA1/ERN1/C9orf72/KRAS/ATG2A/BIRC6/CTSD/ATG4B/ATG7/IRS2/MAPK8/HRAS/ATG16L2/UBA52/RRAGA/WDR41/ITPR1/PRKCQ/ATG2B/MAP3K7/ATG10/RPS27A/LAMP1/PRKACA/PDPK1
Human Diseases	Neurodegenerative disease	hsa05014	Amyotrophic lateral sclerosis	198/3489	371/8538	0.53369	1.30601	5.00947	4.55E-07	1.05E-05	6.68E-06	CASP1/PSMA4/PSMA1/PSMA6/ATG13/NDUFA9/PSMD11/PSMB7/OPTN/PSMA3/TUBA1A/PSMA2/TNFRSF1B/PSMC4/NOS2/NDUFS2/ANG/PSMA5/PSMB2/SOD1/PSMA7/CASP3/PSMD3/PSMC1/PSMB4/PPP3CC/SPG11/SETX/PSMB1/UQCRFS1/MAP2K3/TBK1/PSMB3/CYCS/NDUFAB1/FIG4/ULK2/PIK3C3/PSMD13/SDHA/PSMC6/PSMB6/CYC1/PSMD7/TUBA1C/TOMM40L/DNAL4/GPX8/PSMD12/CAT/NDUFV2/PSMD14/ACTR10/PSMB5/DERL1/UBQLN1/ADRM1/GPX2/PSMD1/SDHB/DNALI1/ACTR1B/ATXN2L/NUP43/DNAH12/NDUFS3/SQSTM1/TUBB2A/DCTN6/NDUFS6/NDUFV1/BAD/NUP210/GPX3/DNAL1/NDUFB5/TNF/ATG14/NDUFS7/UQCRC1/PSMC2/PPP3R1/COX5A/NDUFB3/ULK1/NRG4/CASP9/PSMD2/KLC1/PFN2/BID/NDUFB7/NUP50/SMCR8/UBQLN2/PSMD6/HNRNPA1/UQCRC2/DNAH6/PFN1/PSMC3/ANXA7/EIF2AK3/DDIT3/NRG1/DNAH5/MAP3K5/NUP98/EIF2S1/ALS2/NDUFS1/COX8A/RAB8A/RAE1/NDUFB10/NDUFA10/TANK/MAPK11/VDAC1/DNAH2/COX7B/TNFRSF1A/ATF4/DCTN4/KLC2/DNAH11/CHCHD10/VCP/TUBB6/PSMD9/NDUFB9/MAP1LC3B/NDUFC2/TUBB4B/NRBF2/PSMD4/XBP1/TUBB/NUP153/COX7A2L/NDUFA1/RAB1A/TRAF2/PPP3CA/RAB5A/NDUFB8/SDHC/MTOR/ANXA11/ACTB/AMBRA1/MAP2K6/ERN1/SDHD/NDUFS8/C9orf72/SEC13/ATG2A/GRIN2D/TUBB3/NDUFA8/GRIN1/NDUFB4/SEH1L/GPX1/KLC4/PSMC5/PSMD8/NUP205/ITPR3/ATF6/CCS/TOMM40/HNRNPA2B1/DCTN3/NUP188/HSPA5/SRSF3/NUP85/NUP35/UBQLN4/WDR41/UQCRH/COX4I1/ATG2B/KIF5B/ALYREF/PPP3CB/NUP214/TUBAL3/ACTR1A/DNAH9/NDUFA5/NDUFV3/NXF1/POM121C/COX7C/UQCR10
Human Diseases	Infectious disease: viral	hsa05165	Human papillomavirus infection	179/3489	333/8538	0.53754	1.31542	4.88057	8.82E-07	1.90E-05	1.21E-05	HLA-E/IRF1/STAT1/HLA-A/HLA-C/HLA-B/BAK1/HLA-F/IRF9/ISG15/STAT2/MX1/CASP8/ATP6V1B2/EIF2AK2/ITGB5/TCIRG1/IKBKG/TICAM1/CASP3/FAS/PSMC1/LAMC2/DVL2/TLR3/TUBG2/CDKN1A/CDK2/TBK1/LAMB2/CREB3L4/COL6A1/MX2/MDM2/RELA/PPP2CA/OASL/ITGAV/LFNG/CCND3/ATP6V0B/ATP6V0D1/ATP6AP1/PRKACB/PPP2R3A/FZD7/WNT7A/ATP6V1H/WNT4/CCND2/VTN/ITGA1/TCF7L1/PARD6G/PTK2/MAGI1/RFNG/IFNAR1/RBPJ/RBL1/FZD3/CREB3L1/COL9A2/MAP2K2/SOS1/RBL2/BAD/LAMC1/NOTCH3/PIK3CD/PPP2CB/TNF/ATP6V0C/ATP6V0E2/PARD6A/HLA-G/IFNAR2/FZD6/DVL1/THBS1/ATP6V1E1/LAMA5/CSNK1A1/CREB1/WNT7B/PARD6B/PIK3R3/PIK3CB/RB1/PTGS2/ITGA6/ATP6V1A/PPP2R5C/JAG1/ITGA3/NOTCH4/ITGA9/TSC2/WNT3/ATP6V0A2/COL4A2/E2F1/MAML3/TNFRSF1A/HDAC2/ITGA5/ITGB8/LAMB3/ATP6V1D/CREB3/AXIN2/IKBKB/IRF3/TSC1/PIK3R1/HES2/CCND1/TCF7L2/ATM/FADD/ITGB6/PSEN1/CREB3L3/CDK4/ATP6V1G1/RPS6KB2/ITGB1/NFKB1/FOXO1/CHUK/NOTCH2/WNT9A/JAK1/ITGA7/LAMA1/PXN/MTOR/ATR/CDKN1B/CCNE2/ITGB7/KRAS/GNAS/CREB5/ITGB4/APC2/PRKCZ/CHAD/DLG1/WNT10A/HES6/THBS3/WNT11/TBP/CRB3/FZD4/UBR4/HRAS/ATP6V1F/TADA3/CHD4/MAML1/LAMA4/COMP/PPP2R2D/MAML2/HES1/PARD3/LLGL2/FZD5/BCAP31/ITGA2B/LAMA3/PRKACA/FZD2/NOTCH1/EP300/TBPL1/CCNE1
Human Diseases	Neurodegenerative disease	hsa05016	Huntington disease	168/3489	311/8538	0.54019	1.32192	4.80732	1.28E-06	2.42E-05	1.55E-05	PSMA4/PSMA1/PSMA6/TGM2/ATG13/NDUFA9/PSMD11/PSMB7/CASP8/PSMA3/TUBA1A/PSMA2/PSMC4/NDUFS2/PPARG/AP2A1/PSMA5/SOD2/PSMB2/SOD1/PSMA7/CASP3/PSMD3/PSMC1/PSMB4/HIP1/PSMB1/UQCRFS1/CREB3L4/PSMB3/CYCS/NDUFAB1/ULK2/VDAC2/PIK3C3/PSMD13/SDHA/SLC1A3/PSMC6/PSMB6/CYC1/PSMD7/POLR2J/TUBA1C/DNAL4/GPX8/PSMD12/HTT/NDUFV2/PSMD14/SIN3A/ACTR10/PSMB5/IFT57/ADRM1/GPX2/PSMD1/SDHB/DNALI1/ACTR1B/DNAH12/NDUFS3/TUBB2A/AP2A2/DCTN6/NDUFS6/NDUFV1/CREB3L1/GPX3/DNAL1/NDUFB5/PLCB4/MAP3K10/ATG14/NDUFS7/UQCRC1/PSMC2/MAPK9/COX5A/NDUFB3/ULK1/STX1A/CASP9/PSMD2/KLC1/SP1/CLTB/NDUFB7/PSMD6/UQCRC2/CLTA/DNAH6/CREB1/CLTC/PSMC3/SLC25A5/DNAH5/MAP3K5/POLR2I/NDUFS1/COX8A/SLC25A4/NDUFB10/NDUFA10/VDAC1/DNAH2/COX7B/HDAC2/DCTN4/KLC2/CREB3/DNAH11/PLCB3/TUBB6/PSMD9/NDUFB9/CREB3L3/NDUFC2/TUBB4B/NRBF2/PSMD4/TUBB/COX7A2L/NDUFA1/TRAF2/NDUFB8/SDHC/MTOR/AMBRA1/ERN1/SDHD/NDUFS8/BBC3/ATG2A/CREB5/TUBB3/VDAC3/NDUFA8/GRIN1/NDUFB4/GPX1/KLC4/PSMC5/TBP/PSMD8/MAPK8/POLR2B/POLR2A/DCTN3/CLTCL1/ITPR1/UQCRH/PPIF/COX4I1/ATG2B/KIF5B/TUBAL3/ACTR1A/DNAH9/NDUFA5/NDUFV3/POLR2E/POLR2J3/EP300/COX7C/TBPL1/MAP2K7/UQCR10
Human Diseases	Neurodegenerative disease	hsa05012	Parkinson disease	149/3489	271/8538	0.54982	1.34546	4.80412	1.33E-06	2.42E-05	1.55E-05	UBE2L6/PSMA4/PSMA1/PSMA6/NDUFA9/UBB/PSMD11/PSMB7/CALM3/UBC/PSMA3/TUBA1A/PSMA2/PSMC4/NDUFS2/MFN2/PSMA5/PSMB2/SOD1/PSMA7/UBA7/CASP3/PSMD3/PSMC1/PSMB4/PSMB1/UQCRFS1/SLC39A1/PLCG1/PSMB3/CYCS/NDUFAB1/VDAC2/PSMD13/SDHA/PSMC6/PSMB6/CYC1/PSMD7/TUBA1C/DUSP1/PSMD12/PRKACB/NDUFV2/PSMD14/PSMB5/TRAP1/ADRM1/PSMD1/SDHB/GNAI2/SLC39A11/CALML4/NDUFS3/TUBB2A/NDUFS6/NDUFV1/NDUFB5/ADORA2A/SLC39A14/DRD1/NDUFS7/UQCRC1/PSMC2/SLC39A13/MAPK9/COX5A/TXN/NDUFB3/UBE2J2/CASP9/PSMD2/KLC1/UBE2L3/NDUFB7/SLC11A2/PSMD6/UQCRC2/SLC39A10/KEAP1/UBE2G1/PSMC3/EIF2AK3/SLC25A5/DDIT3/MAP3K5/EIF2S1/NDUFS1/COX8A/SLC25A4/NDUFB10/NDUFA10/VDAC1/COX7B/ATF4/KLC2/TUBB6/PSMD9/GNAI3/NDUFB9/ITPR2/NDUFC2/TUBB4B/PSMD4/XBP1/TUBB/COX7A2L/NDUFA1/CAMK2G/SNCA/NDUFB8/SDHC/ERN1/MAOB/SDHD/NDUFS8/CALM1/GNAS/CAMK2D/MFN1/TUBB3/VDAC3/NDUFA8/NDUFB4/KLC4/TXN2/PSMC5/PSMD8/MAPK8/ITPR3/UBA52/UBA1/ATF6/MAOA/HSPA5/ITPR1/UQCRH/PPIF/COX4I1/KIF5B/TUBAL3/RPS27A/NDUFA5/NDUFV3/PRKACA/SLC39A5/COX7C/SLC39A3/UQCR10
Human Diseases	Infectious disease: viral	hsa05168	Herpes simplex virus 1 infection	106/3489	182/8538	0.58242	1.42525	4.82033	1.33E-06	2.42E-05	1.55E-05	TAP1/HLA-E/TAP2/B2M/STAT1/HLA-A/HLA-C/HLA-DMA/HLA-B/BAK1/CD74/OAS2/HLA-F/SP100/OAS3/IRF9/HLA-DRA/JAK2/STAT2/IFIH1/HLA-DPB1/PML/ZC3HAV1/CASP8/HLA-DPA1/BIRC3/HLA-DOB/HLA-DMB/EIF2AK2/HLA-DRB1/BST2/IKBKG/TAPBP/TICAM1/TRAF6/CASP3/FAS/CCL2/PDIA3/TLR3/TBK1/CYCS/TRAF5/MYD88/RELA/CCL5/IRAK1/HLA-DQB1/IFNAR1/BAD/PIK3CD/TNF/HLA-G/HLA-DOA/IFNAR2/CASP9/C3/OAS1/IFNGR2/IFNGR1/BID/RNASEL/PIK3R3/EIF2AK3/PIK3CB/SOCS3/EIF2S1/BIRC2/CALR/TSC2/HLA-DRB5/TNFRSF1A/ITGA5/IKBKB/IRF3/TSC1/PIK3R1/SRSF9/FADD/HLA-DQA1/NFKB1/IRF7/CHUK/SRSF8/JAK1/TRAF2/TNFRSF14/MTOR/EIF2B2/POU2F1/EIF2AK4/IL12A/IL1B/EIF2AK1/SRC/PPP1CC/TAB2/SRSF3/HLA-DQB2/MAVS/ALYREF/MAP3K7/HLA-DQA2/SRSF1/NXF1/TAB1

Human Diseases	Neurodegenerative disease	hsa05017	Spinocerebellar ataxia	87/3489	144/8538	0.60417	1.47847	4.81338	1.45E-06	2.49E-05	1.59E-05	PSMA4/PSMA1/PSMA6/ATP2A3/ATG13/PSMD11/PSMB7/PSMA3/PSMA2/PSMC4/PSMA5/PSMB2/PSMA7/PRKCG/PSMD3/PSMC1/PSMB4/PSMB1/GTF2B/PSMB3/CYCS/ULK2/VDAC2/PIK3C3/PSMD13/PSMC6/PSMB6/PSMD7/PSMD12/PSMD14/PSMB5/ADRM1/PSMD1/ATXN2L/RBPJ/PIK3CD/PLCB4/ATG14/PSMC2/MAPK9/KCNC3/ULK1/PSMD2/SP1/PSMD6/SPTBN2/PSMC3/PIK3R3/SLC25A5/PIK3CB/MAP3K5/SLC25A4/VDAC1/PIK3R1/PLCB3/PSMD9/ATXN3/ITPR2/NRBF2/PSMD4/XBP1/TRAF2/MTOR/NOP56/KAT5/GRIN3A/AMBRA1/ERN1/ATG2A/GRIN2D/VDAC3/GRIN1/RORA/PRKCA/OMA1/PSMC5/TBP/PSMD8/MAPK8/PUM1/ITPR3/OPA1/ITPR1/PPIF/ATG2B/ATP2A2/TBPL1

(E) Gene Set Enrichment Analysis (GSEA) : GO BP (Biological Process)

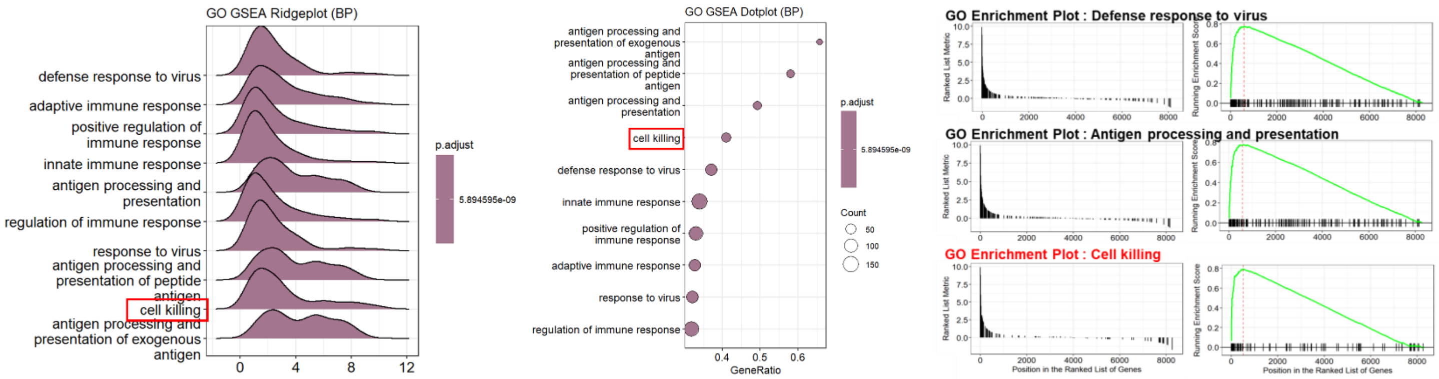

Top 20 enriched GO biological process (BP) term

ID	Description	set Size	ES	NES	p-value	p.adjust	q-value	leading edge	Core enrichment
GO:0051607	defense response to virus	186	0.77563	2.89360	1.00E-10	5.89E-09	4.73E-09	tags=37%, list=7%, signal=35%	GBP5/CXCL9/CXCL10/IFI44L/BST2/OAS2/IFITM1/GBP1/NLRC5/ZBP1/FGL2/CD40/APOBEC3G/IFI6/RSAD2/IFIT3/ISG15/GBP2/MX1/STAT1/OAS3/CASP1/IRF1/SAMHD1/IFITM3/IFIT1/PARP9/DDX60/AIM2/OASL/IFIT5/IFI27/MX2/RTP4/USP18/IFIH1/IFI16/APOBEC3D/ISG20/IFIT2/STAT2/PMAIP1/IRF9/OAS1/DDX60L/DTX3L/NLRP6/TRIM22/IL15/GBP3/MICB/MLKL/ZNFX1/TRIM31/TRIM21/DHX58/HERC5/EIF2AK2/IFITM2/APOBEC3H/TLR3/PML/MOV10/ADAR/APOBEC3F/ZC3HAV1/TRIM56/ZDHHC11/TRIM38
GO:0002250	adaptive immune response	202	0.75353	2.83280	1.00E-10	5.89E-09	4.73E-09	tags=33%, list=6%, signal=31%	SERPING1/HLA-DPA1/HLA-DPB1/HLA-DOA/HLA-DRA/HLA-DQA1/C1S/HLA-DRB1/HLA-DRB5/HLA-DQB1/CD74/HLA-DQA2/TNFSF13B/HLA-DQB2/HLA-DMB/CD274/C2/CD40/C1R/HLA-DMA/RSAD2/CFI/HLA-F/HLA-B/CLC/HLA-DOB/LAMP3/TAP1/ICAM1/IRF1/BTN3A1/HLA-E/SECTM1/HLA-C/TAP2/IL18BP/B2M/BTN3A3/HLA-A/PRDM1/BTN3A2/ASCL2/CD7/C3/JAK2/CTSS/ADA/IL12A/ERAP2/MICB/LAT2/PRKCQ/HLA-G/ALCAM/TNFRSF1B/IL18R1/C8G/CD55/IL6R/FCER1G/PARP3/RNF19B/IL20RB/SOCS3/CEACAM1/RIPK2
GO:0050778	positive regulation of immune response	348	0.70287	2.80135	1.00E-10	5.89E-09	4.73E-09	tags=33%, list=10%, signal=31%	GBP5/SERPING1/IDO1/HLA-DPA1/HLA-DPB1/HLA-DOA/HLA-DRA/HLA-DQA1/C1S/HLA-DRB1/CFH/HLA-DRB5/HLA-DQB1/CD74/HLA-DQA2/TNFSF13B/HLA-DQB2/HLA-DMB/CD274/GBP1/NLRC5/C2/ZBP1/CD40/C1R/HLA-DMA/CD38/RSAD2/CFI/HLA-F/HLA-B/HLA-DOB/GBP2/SLC15A3/OAS3/CASP1/CD5L/IRF1/BTN3A1/BTN1A1/PARP9/HLA-E/DDX60/SECTM1/HLA-C/TAP2/B2M/IFI35/BTN3A3/HLA-A/AIM2/OASL/BTN3A2/IFIH1/CD7/IFI16/CFB/C3/BIRC3/BTN2A2/EREG/OAS1/JAK2/CTSS/NLRP6/ADA/PDE4B/IL15/NMI/IL12A/PIK3AP1/MICB/ZNFX1/TRIM31/LAT2/PRKCQ/HLA-G/BANK1/DHX58/LGALS9/EIF2AK2/IL18R1/C8G/CD55/FCER1G/CCL5/TLR3/ALPK1/RBCK1/CYLD/NR1H4/CEACAM1/RIPK2/CD47/ZC3HAV1/TRIL/TRIM56/TRIM15/OTUD4/LYN/RASGRP1/WDFY1/PRKD2/TICAM1/NLRC3/PLSCR1/RNF31/S100A14/NOD1/TRIM25/UBR2/USP15/POLR3G/HSPD1/IRF7
GO:0045087	innate immune response	441	0.68968	2.80120	1.00E-10	5.89E-09	4.73E-09	tags=34%, list=10%, signal=32%	GBP5/SERPING1/HLA-DPA1/CXCL10/GBP4/C1S/CFH/CIITA/BST2/CD74/OAS2/CD274/IFITM1/GBP1/NLRC5/C2/ZBP1/NOS2/UBE2L6/CD40/C1R/APOBEC3G/IFI6/RSAD2/CFI/IFIT3/HLA-F/HLA-B/UBD/ISG15/GBP2/MX1/STAT1/SLC15A3/OAS3/CASP1/TRIM17/IRF1/SAMHD1/IFITM3/IFIT1/PARP9/HLA-E/DDX60/APOL1/GBP6/HLA-C/NCF1/B2M/IFI35/TRIM40/HLA-A/PRDM1/AIM2/OASL/IFIT5/IFI27/MX2/USP18/TRIM69/IFIH1/IFI16/APOBEC3D/ISG20/IFIT2/PARP14/STAT2/ASS1/CFB/C3/BIRC3/EREG/OAS1/DTX3L/JAK2/CTSS/NLRP6/TRIM22/IRF8/NMI/IL12A/NUB1/PIK3AP1/SAMD9/

									GBP3/CSF1/ZNFX1/TRIM31/TRIM21/HLA-G/DHX58/SP100/TRAFD1/LGALS9/HERC5/GSDMD/EIF2AK2/IFITM2/APOBEC3H/C8G/CD55/FCER1G/CCL5/TLR3/PML/ADAR/CSF1R/ALPK1/RNF19B/OPTN/LCN2/HK1/CITED1/CYLD/NR1H4/UBA7/CEACAM1/APOBEC3F/RIPK2/CASP8/CD47/ZC3HAV1/TRIL/KYNU/TRIM56/TRIM14/ZDHHC11/TRIM38/GCH1/TRIM26/TRIM15/OTUD4/LYN/RASGRP1/WDFY1/CALCOCO2/USP38/TICAM1/IFNE/NLRC3/PLSCR1/S100A14/NOD1/TRIM25/RAB43/USP15/ENDOD1/POLR3G/HSPD1/IRF7
GO:0019882	antigen processing and presentation	67	0.86248	2.79827	1.00E-10	5.89E-09	4.73E-09	tags=49%, list=4%, signal=47%	HLA-DPA1/HLA-DPB1/HLA-DOA/HLA-DRA/HLA-DQA1/HLA-DRB1/HLA-DRB5/HLA-DQB1/CD74/HLA-DQA2/HLA-DQB2/HLA-DMB/FGL2/HLA-DMA/HLA-F/HLA-B/IFI30/HLA-DOB/TAP1/ICAM1/HLA-E/HLA-C/TAP2/B2M/HLA-A/PSMB8/PSME1/CTSS/ERAP2/LGMN/TAPBPL/MICB/HLA-G
GO:0050776	regulation of immune response	424	0.69096	2.79299	1.00E-10	5.89E-09	4.73E-09	tags=32%, list=10%, signal=30%	GBP5/SERPING1/IDO1/HLA-DPA1/HLA-DPB1/HLA-DOA/HLA-DRA/HLA-DQA1/C1S/HLA-DRB1/CFH/HLA-DRB5/HLA-DQB1/CD74/HLA-DQA2/TNFSF13B/HLA-DQB2/HLA-DMB/CD274/GBP1/NLRC5/C2/ZBP1/FGL2/CD40/C1R/HLA-DMA/CD38/RSAD2/CFI/HLA-F/HLA-B/CLC/ISG15/HLA-DOB/GBP2/SLC15A3/OAS3/CASP1/CD5L/IRF1/SAMHD1/BTN3A1/BTN1A1/PARP9/HLA-E/DDX60/SECTM1/HLA-C/NCF1/TAP2/B2M/IFI35/BTN3A3/HLA-A/AIM2/OASL/BTN3A2/USP18/ASCL2/IFIH1/CD7/IFI16/PARP14/STAT2/CFB/C3/BIRC3/BTN2A2/EREG/OAS1/JAK2/CTSS/NLRP6/ADA/PDE4B/IL15/NMI/IL12A/PIK3AP1/MICB/ZNFX1/TRIM31/LAT2/PRKCQ/TRIM21/HLA-G/BANK1/DHX58/TRAFD1/LGALS9/TNFRSF1B/EIF2AK2/IL18R1/C8G/CD55/FCER1G/PARP3/CCL5/TLR3/ADAR/ALPK1/IL20RB/RBCK1/CYLD/NR1H4/CEACAM1/RIPK2/CASP8/CD47/ZC3HAV1/PSMA1/FGL1/TRIL/TRIM56/TRIM15/OTUD4/LYN/RASGRP1/WDFY1/USP38/PRKD2/TICAM1/NLRC3/SPNS2/PLSCR1/RNF31/S100A14/NOD1/TRIM25/UBR2/USP15/POLR3G/HSPD1/IRF7
GO:0009615	response to virus	237	0.72980	2.79010	1.00E-10	5.89E-09	4.73E-09	tags=32%, list=7%, signal=31%	GBP5/CXCL9/CXCL10/IFI44L/BST2/OAS2/IFITM1/GBP1/NLRC5/ZBP1/FGL2/IFI44/CD40/APOBEC3G/IFI6/RSAD2/IFIT3/CCL22/ISG15/GBP2/MX1/STAT1/OAS3/CASP1/IRF1/SAMHD1/IFITM3/IFIT1/PARP9/DDX60/AIM2/OASL/IFIT5/IFI27/MX2/RTP4/USP18/IFIH1/IFI16/APOBEC3D/ISG20/IFIT2/STAT2/PMAIP1/IRF9/OAS1/DDX60L/DTX3L/JAK2/NLRP6/TRIM22/IL15/NMI/IL12A/GBP3/MICB/MLKL/ZNFX1/TRIM31/TRIM21/DHX58/LGALS9/HERC5/EIF2AK2/IFITM2/APOBEC3H/CCL5/TLR3/PML/MOV10/ADAR/APOBEC3F/ZC3HAV1/TRIM56/ZDHHC11/TRIM38
GO:0048002	antigen processing and presentation of peptide antigen	50	0.89400	2.75751	1.00E-10	5.89E-09	4.73E-09	tags=58%, list=4%, signal=56%	HLA-DPA1/HLA-DPB1/HLA-DOA/HLA-DRA/HLA-DQA1/HLA-DRB1/HLA-DRB5/HLA-DQB1/CD74/HLA-DQA2/HLA-DQB2/HLA-DMB/HLA-DMA/HLA-F/HLA-B/IFI30/HLA-DOB/TAP1/HLA-E/HLA-C/TAP2/B2M/HLA-A/CTSS/ERAP2/LGMN/TAPBPL/MICB/HLA-G
GO:0001906	cell killing	95	0.79588	2.74024	1.00E-10	5.89E-09	4.73E-09	tags=41%, list=6%, signal=39%	GBP5/CXCL9/PLA2G2A/CXCL10/HLA-DRA/HLA-DRB1/CFH/CXCL11/GBP1/NOS2/CCL25/HLA-F/CCL22/HLA-B/GBP2/CD5L/ICAM1/HLA-E/DAO/APOL1/HLA-C/TAP2/B2M/HLA-A/C3/CXCL14/NLRP6/IL12A/GSDMB/GBP3/MICB/HLA-G/LGALS9/CXCL2/C8G/CD55/RNF19B/CEACAM1/MUC7
GO:0019884	antigen processing and presentation of exogenous antigen	35	0.92818	2.73032	1.00E-10	5.89E-09	4.73E-09	tags=66%, list=4%, signal=64%	HLA-DPA1/HLA-DPB1/HLA-DOA/HLA-DRA/HLA-DQA1/HLA-DRB1/HLA-DRB5/HLA-DQB1/CD74/HLA-DQA2/HLA-DQB2/HLA-DMB/HLA-DMA/HLA-F/IFI30/HLA-DOB/HLA-E/TAP2/B2M/HLA-A/PSME1/CTSS/LGMN
GO:1903900	regulation of viral life cycle	82	0.79842	2.68082	1.00E-10	5.89E-09	4.73E-09	tags=46%, list=8%, signal=43%	HLA-DRB1/CIITA/BST2/CD74/OAS2/IFITM1/APOBEC3G/RSAD2/ISG15/LAMP3/MX1/OAS3/IFITM3/IFIT1/OASL/IFIT5/IL32/IFIH1/IFI16/APOBEC3D/ISG20/OAS1/PROX1/TRIM22/ZNFX1/TRIM31/TRIM21/LGALS9/EIF2AK2/IFITM2/APOBEC3H/CCL5/ADAR/APOBEC3F/ZC3HAV1/TRIM38/TRIM26/TRIM15
GO:0050792	regulation of viral process	95	0.77729	2.67623	1.00E-10	5.89E-09	4.73E-09	tags=43%, list=8%, signal=40%	HLA-DRB1/CIITA/BST2/CD74/OAS2/IFITM1/APOBEC3G/RSAD2/ISG15/LAMP3/MX1/STAT1/OAS3/IFITM3/IFIT1/OASL/IFIT5/IL32/IFIH1/IFI16/APOBEC3D/ISG20/OAS1/PROX1/TRIM22/ZNFX1/TRIM31/TRIM21/SP100/LGALS9/EIF2AK2/IFITM2/APOBEC3H/CCL5/ADAR/APOBEC3F/ZC3HAV1/TRIM14/TRIM38/TRIM26/TRIM15
GO:0002478	antigen processing and presentation of exogenous peptide antigen	31	0.93800	2.66555	1.00E-10	5.89E-09	4.73E-09	tags=71%, list=4%, signal=69%	HLA-DPA1/HLA-DPB1/HLA-DOA/HLA-DRA/HLA-DQA1/HLA-DRB1/HLA-DRB5/HLA-DQB1/CD74/HLA-DQA2/HLA-DQB2/HLA-DMB/HLA-DMA/HLA-F/IFI30/HLA-DOB/HLA-E/TAP2/B2M/HLA-A/CTSS/LGMN
GO:0048525	negative regulation of viral process	64	0.82522	2.65700	1.00E-10	5.89E-09	4.73E-09	tags=55%, list=8%, signal=51%	CIITA/BST2/CD74/OAS2/IFITM1/APOBEC3G/RSAD2/ISG15/MX1/STAT1/OAS3/IFITM3/IFIT1/OASL/IFIT5/IL32/IFIH1/IFI16/APOBEC3D/ISG20/OAS1/PROX1/ZNFX1/TRIM31/TRIM21/SP100/EIF2AK2/IFITM2/APOBEC3H/CCL5/APOBEC3F/ZC3HAV1/TRIM14/TRIM26/TRIM15
GO:0034341	response to type II interferon	72	0.80807	2.65559	1.00E-10	5.89E-09	4.73E-09	tags=49%, list=7%, signal=46%	GBP5/HLA-DPA1/GBP4/CIITA/BST2/CD74/IFITM1/GBP1/NLRC5/NOS2/CD40/UBD/GBP2/STAT1/CASP1/IRF1/IFITM3/PARP9/GBP6/PARP14/ASS1/JAK2/IRF8/NUB1/GBP3/TRIM21/SP100/LGALS9/IFITM2/CCL5/TLR3/CITED1/CD47/KYNU/GCH1
GO:0002460	adaptive immune response based on somatic recombination of immune receptors built from immunoglobulin superfamily domains	147	0.72081	2.63676	1.00E-10	5.89E-09	4.73E-09	tags=31%, list=6%, signal=29%	SERPING1/HLA-DRA/C1S/HLA-DRB1/CD74/TNFSF13B/CD274/C2/CD40/C1R/RSAD2/CFI/HLA-F/HLA-B/CLC/ICAM1/HLA-E/SECTM1/HLA-C/TAP2/IL18BP/B2M/BTN3A3/HLA-A/BTN3A2/ASCL2/CD7/C3/JAK2/ADA/IL12A/MICB/PRKCQ/HLA-G/TNFRSF1B/IL18R1/C8G/CD55/IL6R/FCER1G/PARP3/IL20RB/SOCS3/CEACAM1/RIPK2

GO:0002684	positive regulation of immune system process	495	0.64177	2.63082	1.00E-10	5.89E-09	4.73E-09	tags=23%, list=6%, signal=23%	GBP5/SERPING1/IDO1/HLA-DPA1/HLA-DPB1/CXCL10/HLA-DOA/HLA-DRA/HLA-DQA1/C1S/HLA-DRB1/CFH/HLA-DRB5/HLA-DQB1/CD74/HLA-DQA2/TNFSF13B/HLA-DQB2/HLA-DMB/CD274/GBP1/NLRC5/C2/ZBP1/NOS2/CD40/C1R/HLA-DMA/CX3CL1/CD38/RSAD2/CFI/HLA-F/HLA-B/HLA-DOB/GBP2/SLC15A3/OAS3/CASP1/IL7/CD5L/SOCS1/ICAM1/IRF1/BTN3A1/BTN1A1/PARP9/HLA-E/DDX60/SECTM1/HLA-C/TAP2/B2M/IFI35/BTN3A3/HLA-A/AIM2/OASL/BTN3A2/IL15RA/ASCL2/IFIH1/CD7/IFI16/CFB/C3/BIRC3/BTN2A2/EREG/OAS1/CAMK1D/JAK2/CTSS/NLRP6/ADA/PDE4B/IL15/NMI/IL12A/PIK3AP1/DPP4/LGMN/MICB/MDK/CSF1/ZNFX1/TRIM31/LAT2/PRKCQ/HLA-G/BANK1/DHX58/ITGA2B/LGALS9/EIF2AK2/IL18R1/GPR68/C8G/CD55/IL6R/FCER1G/CCL5/TLR3/CSF1R/ALPK1/HK1/RBCK1/CYLD/NR1H4/CEACAM1/RIPK2/CASP8/CD47
GO:0002831	regulation of response to biotic stimulus	276	0.66679	2.59600	1.00E-10	5.89E-09	4.73E-09	tags=33%, list=10%, signal=31%	GBP5/SERPING1/HLA-DRB1/CFH/CD274/NLRC5/ZBP1/FGL2/CX3CL1/APOBEC3G/RSAD2/HLA-F/HLA-B/ISG15/GBP2/STAT1/SLC15A3/OAS3/CASP1/IRF1/SAMHD1/PARP9/HLA-E/DDX60/NCF1/IFI35/HLA-A/AIM2/OASL/USP18/IFIH1/IFI16/PARP14/STAT2/BIRC3/CARD16/EREG/OAS1/DTX3L/CTSS/NLRP6/TRIM22/IL15/NMI/IL12A/PIK3AP1/MICB/ZNFX1/TRIM31/TRIM21/HLA-G/DHX58/TRAFD1/LGALS9/HERC5/EIF2AK2/CD55/CCL5/TLR3/PML/ADAR/ALPK1/OPTN/CYLD/NR1H4/CEACAM1/APOBEC3F/RIPK2/CASP8/ZC3HAV1/TRIL/TRIM56/ZDHHC11/TRIM38/TRIM15/OTUD4/LYN/RASGRP1/WDFY1/USP38/TICAM1/NLRC3/PLSCR1/RNF31/S100A14/NOD1/TRIM25/USP15/POLR3G/HSPD1/IRF7
GO:0045069	regulation of viral genome replication	50	0.84115	2.59450	1.00E-10	5.89E-09	4.73E-09	tags=54%, list=7%, signal=51%	BST2/OAS2/IFITM1/APOBEC3G/RSAD2/ISG15/MX1/OAS3/IFITM3/IFIT1/OASL/IFIT5/IFIH1/IFI16/APOBEC3D/ISG20/OAS1/PROX1/ZNFX1/EIF2AK2/IFITM2/APOBEC3H/CCL5/ADAR/APOBEC3F/ZC3HAV1/TRIM38
GO:0002504	antigen processing and presentation of peptide or polysaccharide antigen via MHC class II	26	0.93422	2.59172	1.00E-10	5.89E-09	4.73E-09	tags=69%, list=4%, signal=67%	HLA-DPA1/HLA-DPB1/HLA-DOA/HLA-DRA/HLA-DQA1/HLA-DRB1/HLA-DRB5/HLA-DQB1/CD74/HLA-DQA2/HLA-DQB2/HLA-DMB/HLA-DMA/IFI30/HLA-DOB/B2M/CTSS/LGMN

ES, enrichment Score; NES, normalized enrichment Score

(F) GSEA (Gene Set Enrichment Analysis): Canonical Pathways (C2)

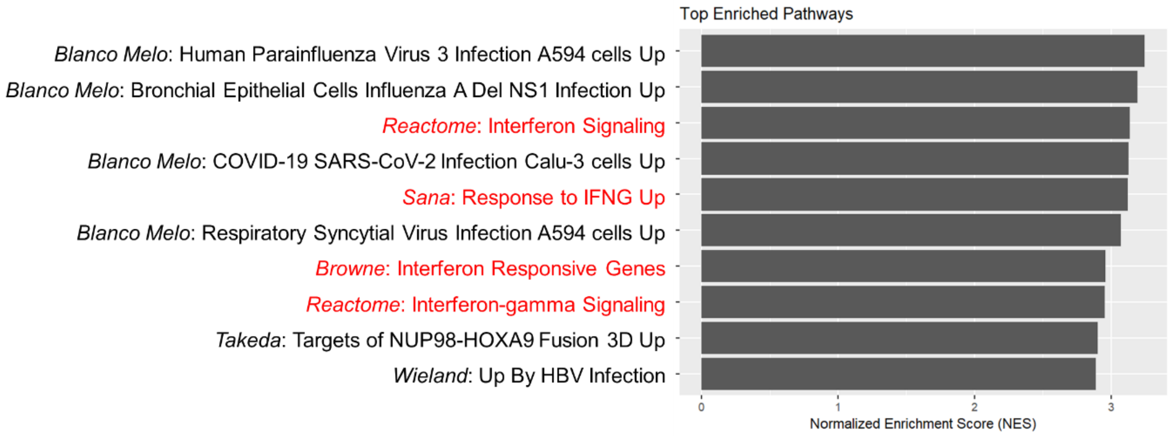

Supplementary Material S4. REACTOME Network Plot

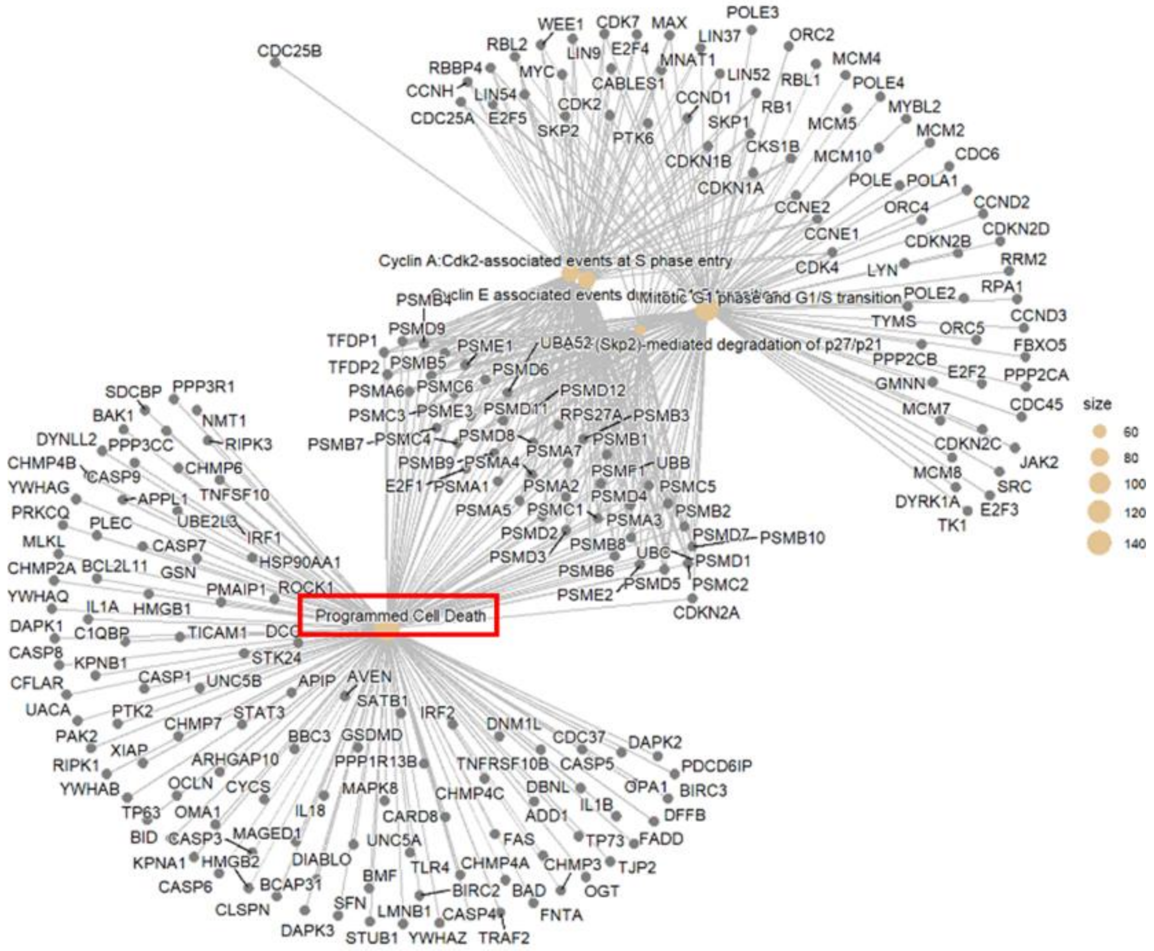

Supplementary Material S5. Morphology of intestinal organoids and number of isolated intestinal epithelial cells used for single-cell RNA-sequencing

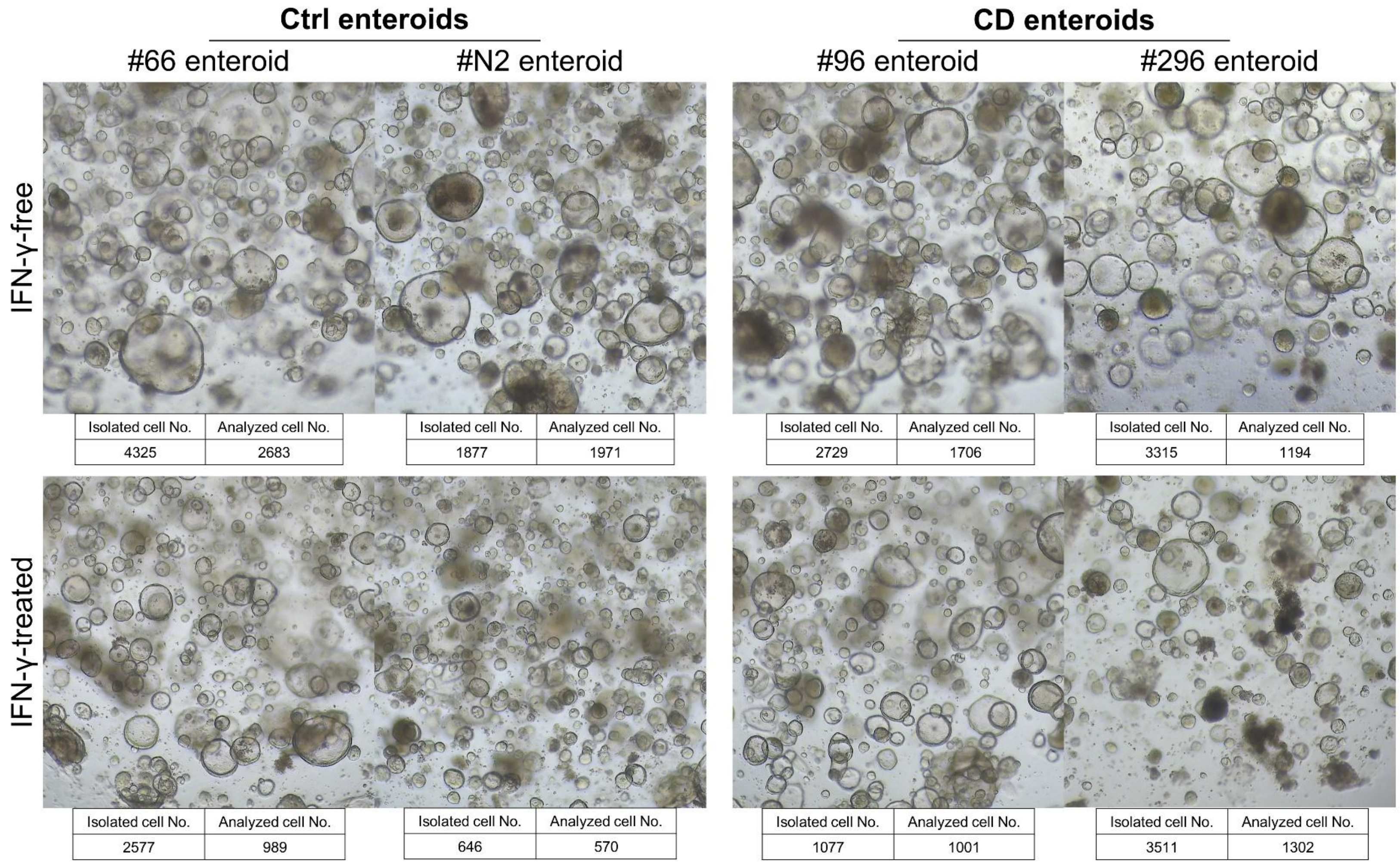

		Isolated cell No.	Analyzed cell No.
#66 enteroid	IFN-γ-free	4325	2683
#N2 enteroid	IFN-γ-free	1877	1971
#96 enteroid	IFN-γ-free	2729	1706
#296 enteroid	IFN-γ-free	3315	1194
#66 enteroid	IFN-γ-treated	2577	989
#N2 enteroid	IFN-γ-treated	646	570
#96 enteroid	IFN-γ-treated	1077	1001
#296 enteroid	IFN-γ-treated	3511	1302

Supplementary Material S6. Differentially expressed genes in cell clusters. (A) Top 10 differentially expressed genes in each cluster. (B) Top 10 gene ontology categories enriched with differentially expressed genes in each cluster identified by Gene Set Enrichment Analysis (GSEA)

(A) Top 10 differentially expressed genes (positively differentially expressed gene only, minimum percentage of cells in cluster > 25%, and logFC > 0.25) in each cluster

Cluster	Gene symbol	Average log2 fold change (logFC)	Percentage of cells where the gene is detected in the cluster	Percentage of cells where the gene is detected on average in the other clusters	*p*-value	Adjusted *p*-value, (Bonferroni correction)
0	MT1X	1.10	85.0%	64.3%	8.78E-176	1.60E-171
	CLIC3	0.60	74.9%	50.1%	8.59E-101	1.56E-96
	TFF1	0.54	98.6%	91.1%	2.09E-113	3.80E-109
	SLC6A8	0.54	86.0%	58.6%	2.05E-150	3.73E-146
	SLURP2	0.45	27.5%	13.3%	9.62E-51	1.75E-46
	CA9	0.44	71.9%	44.1%	1.31E-93	2.39E-89
	TFF2	0.44	87.6%	68.7%	4.62E-85	8.39E-81
	NDRG1	0.39	75.2%	49.2%	4.44E-102	8.07E-98
	ANKRD37	0.37	36.2%	18.0%	3.38E-65	6.15E-61
	DUOXA2	0.36	92.4%	70.8%	4.68E-123	8.50E-119
1	UBD	0.57	64.7%	51.8%	9.54E-42	1.74E-37
	MT-ATP8	0.43	82.3%	61.1%	5.08E-74	9.24E-70
	PI3	0.42	66.5%	48.7%	1.43E-41	2.59E-37
2	S100P	2.64	86.3%	63.3%	4.94E-221	8.98E-217
	GAS5	2.30	88.4%	59.5%	3.85E-245	7.01E-241

	RPS2	2.10	99.9%	90.7%	0	0
	RPL21	2.07	100.0%	89.4%	0	0
	RPL10A	2.06	99.9%	85.8%	0	0
	RPS8	2.06	100.0%	97.0%	0	0
	RPL17	2.03	100.0%	95.0%	0	0
	RPS16	2.02	100.0%	90.6%	0	0
	RPL37A	2.02	99.9%	88.2%	0	0
	SNHG8	2.00	96.7%	67.9%	0	0
4	MT1X	2.42	91.6%	64.7%	1.50E-255	2.73E-251
	SLC6A8	1.90	96.4%	59.0%	0	0
	ERO1A	1.88	99.3%	74.7%	0	0
	TFF1	1.87	99.5%	91.4%	1.01E-235	1.84E-231
	DUOXA2	1.78	97.9%	71.3%	2.47E-302	4.49E-298
	SCD	1.78	97.3%	74.4%	5.66E-274	1.03E-269
	NDRG1	1.70	88.8%	49.2%	3.58E-272	6.52E-268
	IGFBP3	1.69	75.0%	53.7%	1.69E-89	3.07E-85
	TFF2	1.62	94.6%	69.0%	9.84E-219	1.79E-214
	EGLN3	1.58	94.9%	55.1%	0	0
5	ALDH1A3	3.21	95.5%	41.7%	0	0
	PLAAT4	3.07	95.9%	74.0%	6.90E-171	1.25E-166
	CXCL5	3.07	77.2%	58.7%	7.72E-114	1.40E-109
	MMP7	2.73	95.9%	75.5%	7.80E-107	1.42E-102
	CCND1	2.65	99.2%	55.6%	0	0
	IFI6	2.60	90.2%	61.4%	2.05E-110	3.73E-106
	CCND2	2.31	45.7%	21.9%	1.12E-65	2.04E-61

	CCN2	2.20	79.5%	28.2%	4.49E-238	8.16E-234
	DCBLD2	2.18	83.6%	27.4%	1.14E-307	2.06E-303
	KRT7	2.13	99.9%	90.0%	3.44E-257	6.25E-253
7	NEAT1	3.36	99.1%	84.4%	4.82E-218	8.76E-214
	MALAT1	3.19	99.6%	91.6%	3.07E-215	5.58E-211
	MTRNR2L12	2.09	76.1%	23.3%	4.86E-215	8.83E-211
	WSB1	1.87	90.8%	42.2%	3.36E-205	6.11E-201
	XIST	1.82	46.9%	20.3%	1.37E-72	2.50E-68
	KCNQ1OT1	1.81	69.7%	13.7%	3.34E-296	6.08E-292
	PARP14	1.77	85.7%	39.0%	7.03E-177	1.28E-172
	DST	1.66	91.3%	54.0%	4.33E-156	7.87E-152
	VMP1	1.54	88.7%	47.6%	2.20E-170	4.01E-166
	CYP3A5	1.41	89.6%	47.4%	2.26E-155	4.11E-151
8	MT1G	3.30	36.8%	12.2%	1.92E-64	3.50E-60
	MT2A	2.57	41.8%	22.4%	6.80E-32	1.24E-27
	PHGR1	2.54	99.6%	95.4%	2.01E-164	3.65E-160
	KRT20	2.41	84.2%	31.5%	3.15E-210	5.72E-206
	FABP2	2.39	84.8%	28.5%	1.73E-227	3.15E-223
	PIGR	2.38	99.8%	75.0%	1.54E-219	2.80E-215
	REG4	2.28	93.8%	76.7%	4.46E-109	8.11E-105
	ALDOB	2.27	90.5%	37.9%	1.34E-213	2.44E-209
	RBP2	2.20	55.6%	18.5%	5.98E-107	1.09E-102
	LGALS3	2.05	100.0%	97.2%	3.13E-186	5.70E-182
9	PLA2G2A	0.26	29.5%	14.6%	2.71E-14	4.92E-10
10	UBE2C	3.22	92.1%	3.8%	0	0

	CKS2	3.06	99.7%	42.3%	9.07E-259	1.65E-254
	PTTG1	2.94	94.9%	8.6%	0	0
	HIST1H4C	2.86	84.1%	36.4%	8.31E-150	1.51E-145
	H2AFZ	2.84	100.0%	70.2%	1.03E-215	1.87E-211
	TUBA1B	2.69	100.0%	58.8%	9.29E-222	1.69E-217
	PCLAF	2.66	98.9%	13.1%	0	0
	UBE2S	2.64	97.2%	24.5%	0	0
	HMGB2	2.48	94.6%	12.0%	0	0
	CENPW	2.37	99.4%	27.7%	1.42E-307	2.59E-303
11	APOC3	5.76	77.8%	16.4%	5.01E-226	9.11E-222
	APOA1	5.70	81.4%	16.3%	3.81E-251	6.92E-247
	APOA4	5.25	71.2%	9.8%	2.35E-288	4.27E-284
	RBP2	4.72	89.5%	18.1%	3.16E-283	5.75E-279
	ANPEP	3.57	88.9%	11.1%	0	0
	SELENOP	3.20	84.6%	11.8%	0	0
	ALDOB	3.20	98.4%	38.5%	6.06E-209	1.10E-204
	MT1G	3.01	50.3%	12.2%	5.00E-100	9.09E-96
	FABP2	2.85	95.1%	29.1%	7.56E-213	1.38E-208
	APOB	2.67	70.6%	4.4%	0	0
12	APOC3	2.67	77.6%	16.6%	2.10E-183	3.81E-179
	APOA1	2.67	77.2%	16.6%	4.45E-181	8.08E-177
	RBP2	2.24	90.7%	18.3%	1.38E-230	2.51E-226
	APOA4	2.16	63.8%	10.2%	4.83E-182	8.79E-178
	ANPEP	1.64	80.2%	11.6%	5.15E-248	9.36E-244
	MT1G	1.61	54.1%	12.3%	1.09E-100	1.99E-96

	SELENOP	1.14	69.4%	12.4%	4.03E-167	7.32E-163
	PRAP1	1.01	92.5%	69.4%	5.09E-46	9.25E-42
	MT1H	0.99	26.1%	3.5%	2.54E-79	4.62E-75
	ALDOB	0.89	87.3%	39.0%	8.93E-76	1.62E-71
14	IGFBP3	1.51	90.8%	55.0%	9.01E-57	1.64E-52
	CCN2	1.50	78.9%	30.4%	1.32E-71	2.41E-67
	SLC2A3	1.49	43.9%	4.0%	1.48E-174	2.70E-170
	LOXL4	1.40	78.9%	24.3%	1.39E-93	2.53E-89
	HSPG2	1.33	99.6%	63.8%	1.56E-76	2.85E-72
	LAMC2	1.26	89.5%	47.7%	1.51E-62	2.74E-58
	CDA	1.19	96.5%	66.8%	1.47E-60	2.68E-56
	F3	1.19	99.6%	67.4%	5.12E-62	9.31E-58
	SLC2A1	1.19	93.9%	54.8%	2.05E-62	3.72E-58
	CST6	1.16	68.9%	21.1%	8.50E-79	1.55E-74
15	UBD	3.14	89.6%	53.0%	1.29E-54	2.35E-50
	LTB	2.81	74.0%	16.1%	1.01E-109	1.84E-105
	CLDN18	2.42	61.3%	10.0%	1.46E-114	2.65E-110
	PLAU	2.41	79.8%	12.8%	1.55E-168	2.83E-164
	CCL20	2.30	63.6%	29.2%	2.26E-31	4.12E-27
	CXCL8	2.27	78.0%	30.4%	2.99E-59	5.44E-55
	SERPINB7	2.05	52.0%	8.4%	5.73E-95	1.04E-90
	CXCL1	2.02	86.1%	48.8%	2.68E-46	4.87E-42
	ISG20	2.00	93.6%	47.4%	3.75E-78	6.81E-74
	SAA1	2.00	52.6%	21.4%	1.70E-28	3.10E-24
16	ALDH1A3	1.38	84.1%	44.8%	1.16E-16	2.10E-12

	CCND2	1.33	53.7%	23.1%	2.32E-13	4.23E-09
	MT-ND3	1.33	100.0%	92.5%	7.27E-17	1.32E-12
	GAS5	1.33	95.1%	62.9%	2.93E-25	5.32E-21
	S100P	1.32	92.7%	66.0%	4.18E-19	7.60E-15
	CCND1	1.29	87.8%	58.1%	6.95E-14	1.26E-09
	DDX21	1.27	90.2%	35.2%	9.04E-36	1.64E-31
	EIF5B	1.23	98.8%	47.5%	1.68E-33	3.05E-29
	ENC1	1.23	75.6%	41.2%	2.86E-15	5.19E-11
	AC020656.1	1.21	95.1%	43.3%	6.37E-32	1.16E-27

* If no features pass the logFC threshold, DEGs were not identified or a limited number of DEGs were identified.

(B) Top 10 gene ontology categories enriched in each cluster identified by Gene Set Enrichment Analysis (GSEA) of Differentially Expressed Genes (DEGs)

Cluster	Gene ontology	*p*-value	FDR *q*-value
0	GOMF_Lyase_Activity [200]	1.35 e^{-6}	1.1 e^{-2}
	GOMF_Carbon_Carbon_Lyase_Activity [52]	1.37 e^{-6}	1.1 e^{-2}
	HP_Porokeratosis [8]	4.69 e^{-6}	1.93 e^{-2}
	GOBP_Fructose_1_6_Bisphosphate_Metabolic_Process [9]	6.03 e^{-6}	1.93 e^{-2}
	GOMF_Aldehyde_Lyase_Activity [9]	6.03 e^{-6}	1.93 e^{-2}
	GOBP_Fructose_Metabolic_Process [14]	1.52 e^{-5}	4.06
2	GOCC_Cytosolic_Ribosome [118]	1.72 e^{-172}	2.75 e^{-168}
	GOBP_Cytoplasmic_Translation [156]	2.39 e^{-166}	1.92 e^{-162}
	GOMF_Structural_Constituent_Of_Ribosome [169]	9.34 e^{-154}	4.98 e^{-150}
	GOCC_Ribosomal_Subunit [188]	1.53 e^{-152}	6.13 e^{-149}

	GOCC_Ribosome [239]	4.53 e^{-149}	1.45 e^{-145}
	GOBP_Peptide_Biosynthetic_Process [866]	6.64 e^{-115}	1.77 e^{-111}
	GOBP_Peptide_Metabolic_Process [997]	6.95 e^{-110}	1.59 e^{-106}
	GOBP_Amide_Biosynthetic_Process [1004]	1.23 e^{-109}	2.46 e^{-106}
	GOMF_Structural_Molecule_Activity [809]	1.17 e^{-101}	2.07 e^{-98}
	GOBP_Amide_Metabolic_Process [1301]	1.72 e^{-100}	2.76 e^{-97}
4	GOBP_Sterol_Biosynthetic_Process [68]	7.28 e^{-16}	1.06 e^{-11}
	GOBP_Small_Molecule_Metabolic_Process [1871]	1.32 e^{-15}	1.06 e^{-11}
	GOBP_Small_Molecule_Biosynthetic_Process [608]	6.06 e^{-14}	3.23 e^{-10}
	GOBP_Sterol_Metabolic_Process [155]	9.58 e^{-14}	3.84 e^{-10}
	GOBP_Organic_Hydroxy_Compound_Metabolic_Process [567]	3.08 e^{-13}	9.86 e^{-10}
	GOBP_Alcohol_Metabolic_Process [373]	4.37 e^{-12}	1.17 e^{-8}
	GOBP_Steroid_Metabolic_Process [327]	1.69 e^{-11}	3.85 e^{-8}
	GOBP_Steroid_Biosynthetic_Process [185]	2.03 e^{-11}	4.05 e^{-8}
	GOCC_Nuclear_Outer_Membrane_Endoplasmic_Reticulum_Membrane_Network [1212]	4.38 e^{-11}	7.26 e^{-8}
	GOBP_Negative_Regulation_Of_Molecular_Function [792]	4.54 e^{-11}	7.26 e^{-8}
5	GOBP_Apoptotic_Process [1965]	5.75 e^{-17}	9.2 e^{-13}
	GOCC_Anchoring_Junction [901]	1.3 e^{-16}	1.04 e^{-12}
	GOBP_Regulation_Of_Programmed_Cell_Death [1563]	1.35 e^{-14}	7.2 e^{-11}
	GOBP_Cell_Motility [1813]	4.55 e^{-14}	1.53 e^{-10}
	GOBP_Regulation_Of_Catalytic_Activity [1497]	4.78 e^{-14}	1.53 e^{-10}
	GOBP_Circulatory_System_Development [1224]	6.64 e^{-14}	1.77 e^{-10}
	GOMF_Cell_Adhesion_Molecule_Binding [558]	2.42 e^{-13}	5.53 e^{-10}
	GOBP_Regulation_Of_Multicellular_Organismal_Development [1484]	3.54 e^{-13}	7.09 e^{-10}
	GOBP_Anatomical_Structure_Formation_Involved_In_Morphogenesis [1259]	1.11 e^{-12}	1.97 e^{-9}

	GOMF_Cytoskeletal_Protein_Binding [991]	$1.38\ e^{-12}$	$2.22\ e^{-9}$
7	HP_Centrocecal_Scotoma [13]	$6.7\ e^{-19}$	$1.07\ e^{-14}$
	HP_Leber_Optic_Atrophy [10]	$2.98\ e^{-17}$	$2.38\ e^{-13}$
	HP_Mitochondrial_Respiratory_Chain_Defects [20]	$6.47\ e^{-17}$	$3.45\ e^{-13}$
	HP_Central_Retinal_Vessel_Vascular_Tortuosity [12]	$1.96\ e^{-16}$	$7.83\ e^{-13}$
	HP_Abnormal_Cardiovascular_System_Physiology [1408]	$7.51\ e^{-16}$	$2.41\ e^{-12}$
	HP_Retinal_Telangiectasia [27]	$1.13\ e^{-15}$	$3\ e^{-12}$
	HP_Mitochondrial_Inheritance [29]	$2.17\ e^{-15}$	$4.34\ e^{-12}$
	HP_Vascular_Tortuosity [29]	$2.17\ e^{-15}$	$4.34\ e^{-12}$
	HP_Abnormality_Of_Cardiovascular_System_Electrophysiology [542]	$6.61\ e^{-15}$	$1.18\ e^{-11}$
	HP_Slow_Decrease_In_Visual_Acuity [34]	$9.09\ e^{-15}$	$1.46\ e^{-11}$
8	GOCC_Inner_Mitochondrial_Membrane_Protein_Complex [158]	$1.95\ e^{-47}$	$3.12\ e^{-43}$
	GOBP_Oxidative_Phosphorylation [148]	$4.69\ e^{-44}$	$3.75\ e^{-40}$
	GOCC_Mitochondrial_Protein_Containing_Complex [300]	$1.54\ e^{-42}$	$7.89\ e^{-39}$
	GOBP_Aerobic_Respiration [197]	$1.97\ e^{-42}$	$7.89\ e^{-39}$
	GOBP_Cellular_Respiration [243]	$9.69\ e^{-40}$	$3.1\ e^{-36}$
	GOBP_Generation_Of_Precursor_Metabolites_And_Energy [511]	$7.86\ e^{-37}$	$2.1\ e^{-33}$
	GOCC_Organelle_Inner_Membrane [553]	$9.95\ e^{-36}$	$2.28\ e^{-32}$
	GOBP_Energy_Derivation_By_Oxidation_Of_Organic_Compounds [337]	$1.25\ e^{-35}$	$2.51\ e^{-32}$
	GOCC_Mitochondrial_Envelope [804]	$1.96\ e^{-33}$	$3.34\ e^{-30}$
	GOBP_Atp_Synthesis_Coupled_Electron_Transport [102]	$2.29\ e^{-33}$	$3.34\ e^{-30}$
10	GOBP_Cell_Cycle [1841]	$1.12\ e^{-29}$	$1.8\ e^{-25}$
	GOBP_Cell_Cycle_Process [1323]	$8.69\ e^{-29}$	$6.95\ e^{-25}$
	GOBP_Mitotic_Cell_Cycle [932]	$1.44\ e^{-28}$	$7.66\ e^{-25}$
	GOBP_Chromosome_Organization [630]	$5.41\ e^{-28}$	$2.16\ e^{-24}$

	GOBP_Cell_Division [639]	$5.28\ e^{-25}$	$1.69\ e^{-21}$
	GOBP_Chromosome_Segregation [441]	$1.4\ e^{-24}$	$3.74\ e^{-21}$
	GOBP_Organelle_Fission [491]	$1.61\ e^{-23}$	$3.69\ e^{-20}$
	GOBP_Dna_Metabolic_Process [1037]	$2.42\ e^{-23}$	$4.84\ e^{-20}$
	GOBP_Mitotic_Cell_Cycle_Process [775]	$6.94\ e^{-23}$	$1.23\ e^{-19}$
	GOBP_Mitotic_Nuclear_Division [277]	$4.47\ e^{-21}$	$7.16\ e^{-18}$
11	GOBP_Lipid_Metabolic_Process [1407]	$7.44\ e^{-20}$	$1.19\ e^{-15}$
	GOBP_Small_Molecule_Metabolic_Process [1871]	$1.52\ e^{-18}$	$1.22\ e^{-14}$
	GOBP_Cellular_Lipid_Metabolic_Process [1005]	$7.17\ e^{-18}$	$3.83\ e^{-14}$
	GOCC_Brush_Border [108]	$2.79\ e^{-17}$	$1.12\ e^{-13}$
	GOBP_Intestinal_Absorption [41]	$4.37\ e^{-16}$	$1.4\ e^{-12}$
	GOCC_Cluster_Of_Actin_Based_Cell_Projections [165]	$5.08\ e^{-15}$	$1.35\ e^{-11}$
	GOBP_Organic_Acid_Metabolic_Process [980]	$9.01\ e^{-15}$	$2.06\ e^{-11}$
	GOBP_Hormone_Metabolic_Process [241]	$1.7\ e^{-14}$	$3.04\ e^{-11}$
	GOCC_Apical_Part_Of_Cell [471]	$1.81\ e^{-14}$	$3.04\ e^{-11}$
	GOBP_Digestion [134]	$1.9\ e^{-14}$	$3.04\ e^{-11}$
12	GOMF_Lipid_Binding [852]	$2.87\ e^{-10}$	$2.19\ e^{-6}$
	GOBP_Response_To_Zinc_Ion [51]	$3.29\ e^{-10}$	$2.19\ e^{-6}$
	GOBP_Detoxification_Of_Copper_Ion [16]	$4.1\ e^{-10}$	$2.19\ e^{-6}$
	GOCC_Triglyceride_Rich_Plasma_Lipoprotein_Particle [20]	$1.09\ e^{-9}$	$3.76\ e^{-6}$
	GOBP_Detoxification [158]	$1.59\ e^{-9}$	$3.76\ e^{-6}$
	GOBP_Detoxification_Of_Inorganic_Compound [22]	$1.64\ e^{-9}$	$3.76\ e^{-6}$
	GOBP_Intestinal_Lipid_Absorption [22]	$1.64\ e^{-9}$	$3.76\ e^{-6}$
	GOBP_Chemical_Homeostasis [1037]	$1.9\ e^{-9}$	$3.8\ e^{-6}$
	GOBP_Lipid_Localization [503]	$2.17\ e^{-9}$	$3.82\ e^{-6}$

	GOBP_Cellular_Response_To_Zinc_Ion [24]	2.38 e^{-9}	3.82 e^{-6}
14	GOCC_Collagen_Containing_Extracellular_Matrix [430]	1.89 e^{-23}	3.03 e^{-19}
	GOCC_External_Encapsulating_Structure [564]	6.68 e^{-21}	5.35 e^{-17}
	GOBP_Cell_Adhesion [1531]	6.11 e^{-20}	3.26 e^{-16}
	GOCC_Cell_Substrate_Junction [434]	4.63 e^{-19}	1.85 e^{-15}
	GOCC_Anchoring_Junction [901]	6.03 e^{-19}	1.93 e^{-15}
	GOBP_Cell_Motility [1813]	6.45 e^{-18}	1.72 e^{-14}
	GOBP_Anatomical_Structure_Formation_Involved_In_Morphogenesis [1259]	8.22 e^{-18}	1.88 e^{-14}
	GOMF_Extracellular_Matrix_Structural_Constituent [166]	1.32 e^{-16}	2.65 e^{-13}
	GOMF_Cell_Adhesion_Molecule_Binding [558]	8.82 e^{-16}	1.48 e^{-12}
	HP_Hypoplastic_Dermoepidermal_Hemidesmosomes [7]	9.27 e^{-16}	1.48 e^{-12}
15	GOBP_Biological_Process_Involved_In_Interspecies_Interaction_Between_Organisms [1759]	4.7 e^{-27}	7.52 e^{-23}
	GOBP_Cell_Adhesion [1531]	1.98 e^{-24}	1.59 e^{-20}
	GOBP_Defense_Response [1855]	8.8 e^{-22}	4.7 e^{-18}
	GOBP_Locomotion [1335]	1.32 e^{-21}	5.28 e^{-18}
	GOBP_Cell_Motility [1813]	5.76 e^{-20}	1.84 e^{-16}
	GOMF_Signaling_Receptor_Binding [1495]	3.81 e^{-19}	9.28 e^{-16}
	GOBP_Defense_Response_To_Other_Organisnism [1235]	4.06 e^{-19}	9.28 e^{-16}
	GOBP_Response_To_Bacterium [742]	2.26 e^{-18}	4.52 e^{-15}
	GOBP_Wound_Healing [425]	7.65 e^{-18}	1.36 e^{-14}
	GOCC_Anchoring_Junction [901]	9.09 e^{-18}	1.46 e^{-14}
16	HP_Abnormality_Of_The_Upper_Urinary_Tract [1443]	1.64 e^{-16}	1.65 e^{-12}
	GOCC_Cell_Substrate_Junction [434]	2.06 e^{-16}	1.65 e^{-12}
	GOMF_Protein_Containing_Complex_Binding [1762]	1.81 e^{-14}	9.63 e^{-11}
	GOMF_Cadherin_Binding [335]	4.57 e^{-14}	1.83 e^{-10}

	GOBP_Cytoplasmic_Translation [156]	$9.18\ e^{-14}$	$2.48\ e^{-10}$
	HP_Abnormal_Renal_Morphology [1118]	$9.3\ e^{-14}$	$2.48\ e^{-10}$
	GOBP_Peptide_Metabolic_Process [997]	$1.17\ e^{-13}$	$2.68\ e^{-10}$
	GOCC_Cytosolic_Ribosome [118]	$2\ e^{-13}$	$3.65\ e^{-10}$
	GOMF_Cell_Adhesion_Molecule_Binding [558]	$2.05\ e^{-13}$	$3.65\ e^{-10}$
	GOBP_Peptide_Biosynthetic_Process [866]	$1.27\ e^{-12}$	$2.03\ e^{-9}$

Supplementary material S7. Epithelial lineage-specific marker gene expression in each cluster of single-cell RNA sequencing. (Bold indicates the epithelial cell lineage marker in each single-cell cluster)

Cluster	Gene symbol	Epithelial cell lineage	Average log2 fold change	Percentage of cells where the gene is detected in the cluster	Percentage of cells where the gene is detected on average in the other clusters	*p*-value	Adjusted *p*-value, (Bonferroni correction)
0	RNF186	Enterocytes	0.3004778	50.3%	28.7%	1.57E-64	2.85E-60
0	DMBT1	Enterocytes	0.2882969	65.2%	45.7%	3.46E-55	6.30E-51
2	GPX2	Paneth cells	1.8443475	100.0%	90.8%	0.00E+00	0.00E+00
2	**OLFM4**	**Crypt cells**	1.7992902	87.6%	63.0%	7.23E-185	1.31E-180
2	LYZ	Paneth cells	1.3852443	99.9%	96.0%	0.00E+00	0.00E+00
2	TNFAIP2	Microfold cells	1.3478442	84.7%	64.1%	3.95E-131	7.19E-127
2	PRSS1	Paneth cells	1.3334085	43.0%	15.7%	5.09E-153	9.25E-149
2	AGR2	Paneth cells	1.3140263	99.8%	89.5%	0.00E+00	0.00E+00
2	GSTM3	Enterocytes	1.2738238	92.7%	58.4%	0.00E+00	0.00E+00
2	KRTCAP3	Enterocytes	1.271556	93.2%	51.5%	0.00E+00	0.00E+00
2	PPARG	Enterocytes	1.2666207	82.1%	45.9%	2.16E-269	3.92E-265
2	KLF5	Enterocytes	1.2602163	87.1%	55.7%	1.54E-233	2.79E-229
2	CD55	Enterocytes	1.2365755	87.7%	67.4%	1.13E-136	2.05E-132
2	ANXA5	Microfold cells	1.2360449	96.0%	68.6%	1.04E-304	1.89E-300
2	KLF3	Tuft cells	1.2147825	74.0%	43.5%	3.42E-175	6.22E-171
2	G0S2	Enterocytes	1.2042635	35.5%	12.4%	3.10E-129	5.64E-125
2	CDC42EP5	Enterocytes	1.1910175	99.7%	84.9%	7.31E-304	1.33E-299
2	ELF3	Enterocytes	1.1852043	97.7%	81.1%	4.80E-215	8.73E-211
2	CD24	Paneth cells	1.1762028	99.6%	89.4%	9.15E-293	1.66E-288
2	TRNP1	Enteroendocrine cells	1.1599809	83.8%	49.4%	8.26E-237	1.50E-232
2	KRT7	Goblet cells	1.1369919	99.5%	89.3%	4.37E-227	7.94E-223
2	FABP1	Enterocytes	1.1308551	98.9%	94.1%	7.60E-117	1.38E-112
2	SLC40A1	Enterocytes	1.1214392	85.0%	67.2%	9.51E-118	1.73E-113

2	CYBA	Microfold cells	1.1114272	100.0%	90.3%	0.00E+00	0.00E+00
2	CFB	Paneth cells	1.1013568	73.2%	54.8%	1.18E-90	2.15E-86
2	PMEPA1	Enterocytes	1.0935637	73.0%	52.4%	2.92E-89	5.32E-85
2	TM4SF20	Paneth cells	1.0372421	70.9%	54.5%	2.59E-55	4.72E-51
2	FABP5	Enteroendocrine cells	1.0247479	63.4%	21.5%	5.01E-287	9.12E-283
2	MARCKSL1	Microfold cells	0.9992996	95.5%	68.0%	1.44E-273	2.61E-269
2	TMEM45B	Enterocytes	0.9856707	90.8%	63.9%	1.48E-181	2.69E-177
2	SOX9	Tuft cells	0.9759621	74.0%	44.7%	1.20E-141	2.18E-137
2	EHF	Enterocytes	0.973753	62.8%	28.2%	7.02E-193	1.28E-188
2	DMBT1	Paneth cells	0.9691498	66.9%	45.8%	4.14E-62	7.53E-58
2	BACE2	Goblet cells	0.9389672	89.4%	59.9%	8.16E-202	1.48E-197
2	CCL20	Microfold cells	0.9353321	38.3%	28.5%	3.55E-16	6.45E-12
2	AHR	Microfold cells	0.9275281	63.8%	31.4%	6.42E-166	1.17E-161
2	MUC4	Goblet cells	0.922067	45.3%	23.5%	9.21E-91	1.67E-86
2	FERMT1	Enterocytes	0.9071143	76.4%	39.7%	7.77E-202	1.41E-197
2	ONECUT2	Microfold cells	0.8963527	60.0%	27.1%	8.44E-170	1.54E-165
2	CFD	Paneth cells	0.8929138	51.0%	21.5%	1.39E-145	2.53E-141
2	PPP1R14D	Enterocytes	0.8878413	80.3%	56.5%	4.24E-141	7.72E-137
2	FUOM	Enterocytes	0.879743	89.9%	58.6%	9.93E-212	1.81E-207
2	PRSS2	Enterocytes	0.879006	41.1%	20.7%	3.22E-76	5.85E-72
2	KLF6	Tuft cells	0.8721274	79.2%	55.5%	2.07E-104	3.77E-100
2	PRR15L	Enterocytes	0.8714892	83.8%	47.7%	7.95E-224	1.45E-219
2	REG4	Enterochromaffin cells	0.8135389	87.6%	75.9%	2.15E-59	3.91E-55
2	CBR1	Enterocytes	0.8027643	82.1%	41.3%	1.01E-248	1.84E-244
2	TFF3	Goblet cells	0.7971737	99.7%	98.9%	2.32E-125	4.22E-121
2	CXCL16	Microfold cells	0.7968075	81.2%	49.4%	2.72E-172	4.95E-168
2	TMPRSS2	Microfold cells	0.7935473	77.0%	56.3%	9.50E-81	1.73E-76
2	IRF2	Microfold cells	0.7901825	56.3%	25.0%	6.03E-166	1.10E-161
2	CDH17	Enterocytes	0.7889938	82.8%	55.3%	2.48E-136	4.52E-132
2	CTSD	Microfold cells	0.7758681	95.7%	83.1%	1.42E-131	2.59E-127
2	NFIB	Microfold cells	0.7547055	50.1%	19.6%	1.29E-167	2.35E-163
2	FOSL2	Microfold cells	0.7276502	53.3%	30.7%	1.02E-89	1.86E-85
2	CTNNB1	Paneth cells	0.717171	70.5%	43.7%	9.28E-117	1.69E-112
2	KCNE3	Crypt cells	0.6969017	70.2%	31.5%	2.71E-205	4.92E-201

2	MMP7	Paneth cells	0.6876848	89.9%	74.9%	1.82E-101	3.30E-97
2	CREB3L1	Goblet cells	0.6684001	73.1%	40.2%	6.98E-159	1.27E-154
2	VEGFA	Enteroendocrine cells	0.6535365	71.0%	48.5%	3.62E-80	6.58E-76
2	PLIN3	Enterocytes	0.6335838	68.3%	39.0%	1.13E-130	2.05E-126
2	IL13RA1	Tuft cells	0.6252154	50.8%	27.9%	6.59E-92	1.20E-87
2	PROM1	Crypt cells	0.6174054	51.5%	27.2%	1.35E-93	2.45E-89
2	PLA2G10	Goblet cells	0.6171797	75.3%	45.3%	7.10E-137	1.29E-132
2	PDHA1	Enterocytes	0.6154997	58.3%	22.9%	2.28E-198	4.15E-194
2	MANF	Goblet cells	0.6046249	67.4%	31.9%	2.59E-172	4.71E-168
2	ALCAM	Enteroendocrine cells	0.5904739	42.3%	19.3%	1.81E-92	3.29E-88
2	NFIC	Microfold cells	0.5820787	43.6%	14.6%	1.98E-168	3.60E-164
2	TMC4	Enterocytes	0.5748455	66.4%	45.9%	3.13E-80	5.69E-76
2	CTSH	Microfold cells	0.5744379	68.2%	36.6%	1.34E-143	2.43E-139
2	GALNT12	Goblet cells	0.5709297	45.9%	17.2%	6.24E-151	1.14E-146
2	KCNN4	Paneth cells	0.5592862	53.6%	23.4%	4.52E-144	8.22E-140
2	KCNQ1	Crypt cells	0.5495315	47.7%	16.1%	7.31E-185	1.33E-180
2	ACSL5	Enterocytes	0.5459609	68.8%	38.7%	7.34E-125	1.33E-120
2	RAC2	Microfold cells	0.507982	53.8%	26.4%	7.68E-114	1.40E-109
2	DGAT1	Enterocytes	0.4980593	66.5%	38.6%	5.98E-108	1.09E-103
2	CDX1	Enterocytes	0.4955692	46.4%	21.8%	4.68E-102	8.50E-98
2	LGR4	Paneth cells	0.4954727	49.8%	25.2%	1.88E-94	3.42E-90
2	CCK	Enteroendocrine cells	0.4851624	44.5%	25.0%	9.59E-63	1.74E-58
2	RAP1A	Paneth cells	0.4795732	49.6%	23.5%	6.22E-109	1.13E-104
2	VNN1	Enterocytes	0.4763629	48.0%	27.4%	1.16E-68	2.10E-64
2	RIPK2	Paneth cells	0.4721917	35.5%	13.4%	1.16E-107	2.10E-103
2	CDX2	Enterocytes	0.4715356	48.2%	23.8%	9.91E-94	1.80E-89
2	VIL1	Enterocytes	0.4678996	72.5%	44.3%	3.25E-100	5.91E-96
2	NUCB2	Enteroendocrine cells	0.4674282	43.7%	18.2%	7.63E-116	1.39E-111
2	SLC11A2	Enterocytes	0.4651626	59.1%	37.2%	7.58E-66	1.38E-61
2	ALOX5	Tuft cells	0.438358	46.7%	23.0%	2.01E-91	3.66E-87
2	IRF6	Microfold cells	0.4328625	46.5%	24.3%	9.99E-79	1.82E-74
2	CEACAM1	Enterocytes	0.4236273	61.4%	45.3%	4.56E-32	8.28E-28
2	CASP6	Enterocytes	0.4023809	43.7%	18.4%	2.30E-111	4.18E-107
2	HNF4A	Enterocytes	0.4018946	47.2%	24.3%	1.03E-83	1.87E-79

2	MAOA	Enterocytes	0.399945	38.6%	20.3%	2.68E-60	4.88E-56
2	GJB2	Microfold cells	0.3841629	30.5%	10.2%	5.69E-108	1.04E-103
2	ABHD11	Enterocytes	0.3674104	42.8%	19.7%	4.82E-90	8.76E-86
2	IL4R	Paneth cells	0.3672267	38.0%	17.9%	7.63E-74	1.39E-69
2	TULP4	Microfold cells	0.3489819	35.7%	17.9%	2.76E-60	5.01E-56
2	FAHD1	Enterocytes	0.3461915	36.9%	15.0%	1.66E-95	3.02E-91
2	GLS	Enterocytes	0.3377488	38.1%	21.9%	3.32E-44	6.04E-40
2	SLC12A2	Crypt cells	0.3205903	64.0%	32.8%	4.39E-124	7.99E-120
2	DEGS2	Enterocytes	0.3183624	36.0%	14.4%	2.41E-92	4.39E-88
2	KLK1	Goblet cells	0.3121654	27.3%	8.7%	4.38E-99	7.96E-95
2	GJB1	Enterochromaffin cells	0.3110341	38.6%	19.3%	7.97E-66	1.45E-61
2	GULP1	Enterocytes	0.3026288	33.0%	15.1%	6.00E-65	1.09E-60
2	RELB	Microfold cells	0.2994006	37.7%	20.1%	1.28E-53	2.32E-49
2	MUC13	Goblet cells	0.2985961	73.3%	55.9%	9.08E-35	1.65E-30
2	BMI1	Crypt cells	0.2896259	27.1%	9.4%	4.14E-86	7.54E-82
2	SLC5A1	Enterocytes	0.2875894	40.6%	25.7%	2.80E-32	5.09E-28
2	NPC1L1	Enterocytes	0.2683014	39.6%	21.9%	6.78E-50	1.23E-45
2	NR5A2	Enterocytes	0.2612851	34.7%	17.3%	8.69E-55	1.58E-50
4	TM4SF20	Paneth cells	1.3097768	75.0%	54.7%	2.54E-77	4.62E-73
4	MUC13	Goblet cells	1.2413327	86.3%	55.2%	1.78E-171	3.25E-167
4	CEACAM1	Enterocytes	1.1959813	83.9%	43.6%	2.29E-229	4.16E-225
4	RNF186	Enterocytes	1.1642879	75.2%	27.3%	1.03E-275	1.87E-271
4	FABP1	Enterocytes	1.1520904	98.2%	94.4%	1.05E-67	1.91E-63
4	CD55	Enterocytes	1.1245983	94.2%	67.5%	1.20E-179	2.18E-175
4	SLC40A1	Enterocytes	1.0942233	95.4%	66.8%	1.33E-203	2.42E-199
4	CCL20	Microfold cells	1.048947	51.1%	27.5%	1.50E-72	2.72E-68
4	TMPRSS2	Microfold cells	1.0075329	88.3%	56.0%	5.41E-157	9.83E-153
4	DMBT1	Paneth cells	1.0011236	70.2%	46.2%	2.43E-72	4.41E-68
4	TMEM45B	Enterocytes	0.9915064	94.8%	64.5%	7.18E-207	1.31E-202
4	CTSD	Microfold cells	0.9321794	99.0%	83.2%	1.62E-175	2.94E-171
4	ELF3	Enterocytes	0.9249493	98.7%	81.6%	3.23E-185	5.87E-181
4	CDC42EP5	Enterocytes	0.8885963	99.1%	85.5%	7.90E-164	1.44E-159
4	TFF3	Goblet cells	0.8690179	99.9%	98.9%	3.97E-134	7.23E-130
4	VEGFA	Enteroendocrine cells	0.8476351	84.7%	47.9%	8.20E-176	1.49E-171

4	SLC11A2	Enterocytes	0.8385241	71.6%	36.7%	1.26E-142	2.30E-138
4	TNFAIP2	Microfold cells	0.8207	87.1%	64.7%	6.02E-77	1.10E-72
4	ADM	Enterochromaffin cells	0.7892354	57.9%	21.6%	8.11E-167	1.48E-162
4	CFB	Paneth cells	0.7567676	84.4%	54.3%	2.90E-119	5.28E-115
4	KLF3	Tuft cells	0.7552432	74.9%	44.6%	3.20E-103	5.82E-99
4	KLF6	Tuft cells	0.7534225	85.8%	55.7%	1.07E-117	1.94E-113
4	BACE2	Goblet cells	0.745421	92.1%	60.7%	7.62E-161	1.39E-156
4	FOSL2	Microfold cells	0.7192228	63.2%	30.5%	1.92E-120	3.50E-116
4	PRAP1	Enterocytes	0.7027482	88.3%	68.1%	2.76E-86	5.02E-82
4	PPP1R14D	Enterocytes	0.6238619	83.9%	57.0%	1.83E-97	3.32E-93
4	SOX9	Tuft cells	0.6126189	72.8%	45.9%	2.49E-77	4.52E-73
4	KLF5	Enterocytes	0.6067136	83.6%	57.3%	2.11E-86	3.84E-82
4	TMC4	Enterocytes	0.5956275	76.9%	45.5%	5.50E-110	1.00E-105
4	**MUC4**	**Goblet cells**	0.539216	48.0%	24.1%	6.84E-65	1.24E-60
4	PPARG	Enterocytes	0.5282967	74.3%	48.1%	1.25E-80	2.28E-76
4	IRF6	Microfold cells	0.5132067	52.2%	24.5%	3.85E-91	7.01E-87
4	CFD	Paneth cells	0.5074274	41.0%	23.6%	4.71E-40	8.56E-36
4	CD24	Paneth cells	0.5032573	98.7%	89.9%	7.02E-95	1.28E-90
4	IRF2	Microfold cells	0.496896	52.3%	26.5%	2.57E-76	4.67E-72
4	ALCAM	Enteroendocrine cells	0.4910988	39.2%	20.4%	9.03E-50	1.64E-45
4	IL13RA1	Tuft cells	0.4701594	56.1%	28.2%	3.91E-81	7.11E-77
4	PMEPA1	Enterocytes	0.4543878	72.5%	53.2%	2.34E-40	4.26E-36
4	OLFM4	Crypt cells	0.4528637	82.7%	64.4%	9.93E-47	1.81E-42
4	PROM1	Crypt cells	0.4503314	47.6%	28.5%	2.99E-44	5.44E-40
4	CDH17	Enterocytes	0.4445248	81.4%	56.5%	1.85E-75	3.37E-71
4	AGR2	Goblet cells	0.4427061	98.8%	90.0%	6.83E-85	1.24E-80
4	KRT20	Goblet cells	0.4308565	43.0%	32.8%	7.89E-15	1.43E-10
4	PRR15L	Enterocytes	0.4302311	74.0%	50.0%	2.48E-59	4.50E-55
4	TRNP1	Enteroendocrine cells	0.4261066	73.6%	51.7%	1.27E-50	2.31E-46
4	IRF7	Tuft cells	0.4122107	39.0%	13.9%	3.58E-104	6.51E-100
4	LYZ	Paneth cells	0.409537	99.9%	96.1%	1.17E-89	2.13E-85
4	PLIN3	Enterocytes	0.4093718	65.0%	40.5%	5.30E-61	9.64E-57
4	NUCB2	Enteroendocrine cells	0.4001016	39.0%	19.6%	1.42E-53	2.58E-49
4	FUOM	Enterocytes	0.3804752	78.8%	60.9%	2.96E-41	5.38E-37

4	VIL1	Enterocytes	0.3570593	66.1%	46.0%	3.07E-42	5.58E-38
4	RAC2	Microfold cells	0.3538066	49.8%	27.8%	6.16E-52	1.12E-47
4	CXCL16	Microfold cells	0.3502451	72.5%	51.5%	1.11E-43	2.02E-39
4	MARCKSL1	Microfold cells	0.3304908	89.9%	69.6%	3.79E-51	6.89E-47
4	LGR4	Paneth cells	0.3240335	47.1%	26.4%	2.73E-48	4.96E-44
4	IL4R	Paneth cells	0.3177515	35.8%	18.8%	5.36E-42	9.74E-38
4	RELB	Microfold cells	0.3176998	39.1%	20.6%	3.16E-47	5.74E-43
4	CYBA	Microfold cells	0.3134769	98.9%	90.8%	8.45E-44	1.54E-39
4	ONECUT2	Microfold cells	0.302114	47.2%	29.6%	1.91E-31	3.47E-27
4	AHR	Microfold cells	0.29998	51.4%	33.9%	6.71E-32	1.22E-27
4	REG4	Enterochromaffin cells	0.2958033	82.1%	76.9%	2.45E-12	4.46E-08
4	GPX2	Paneth cells	0.2931944	97.6%	91.4%	1.40E-38	2.54E-34
4	CTNNB1	Paneth cells	0.2822729	62.6%	45.5%	1.87E-31	3.40E-27
4	MAOA	Enterocytes	0.2797748	36.5%	21.2%	1.07E-30	1.94E-26
4	MANF	Goblet cells	0.2750719	50.9%	34.9%	4.55E-27	8.27E-23
4	SLC15A1	Enterocytes	0.2718359	33.5%	17.7%	3.89E-37	7.07E-33
4	EHF	Enterocytes	0.2682849	46.5%	31.2%	1.67E-25	3.04E-21
4	PLA2G10	Goblet cells	0.26469	68.1%	47.2%	1.38E-35	2.50E-31
4	PHGR1	Goblet cells	0.2631344	98.5%	95.3%	3.58E-23	6.51E-19
4	CREB3L1	Goblet cells	0.2631336	62.2%	42.5%	1.01E-33	1.83E-29
5	**MMP7**	**Paneth cells**	2.7289648	95.9%	75.5%	7.80E-107	1.42E-102
5	KRT7	Goblet cells	2.1298867	99.9%	90.0%	3.44E-257	6.25E-253
5	SPINK4	Paneth cells	1.9687568	29.0%	14.6%	1.19E-30	2.17E-26
5	PMEPA1	Enterocytes	1.8662328	98.3%	52.1%	3.06E-260	5.56E-256
5	SLC12A2	Crypt cells	1.6983929	70.9%	34.5%	1.14E-116	2.07E-112
5	ANXA5	Microfold cells	1.4382588	97.0%	70.4%	1.57E-173	2.85E-169
5	CD24	Paneth cells	1.4040072	99.7%	90.1%	3.96E-182	7.20E-178
5	CCK	Enteroendocrine cells	1.2807094	62.8%	25.1%	2.11E-135	3.83E-131
5	CTNNB1	Paneth cells	1.0944337	74.3%	45.3%	2.20E-76	4.00E-72
5	MARCKSL1	Microfold cells	1.0271872	97.9%	69.7%	4.25E-147	7.72E-143
5	TNFAIP2	Microfold cells	1.010284	87.8%	65.3%	1.44E-77	2.62E-73
5	FERMT1	Enterocytes	1.0095148	84.0%	41.7%	3.37E-146	6.12E-142
5	COL18A1	Enterocytes	0.9677843	58.0%	12.1%	3.69E-260	6.70E-256
5	KCNE3	Crypt cells	0.9604159	61.8%	34.7%	2.05E-67	3.73E-63

5	CYBA	Microfold cells	0.9496354	99.2%	91.0%	5.64E-79	1.03E-74
5	SOX9	Tuft cells	0.9084428	78.8%	46.4%	2.60E-83	4.72E-79
5	PRSS2	Enterocytes	0.8647325	41.2%	22.1%	1.63E-33	2.96E-29
5	TFF3	Goblet cells	0.8302375	100.0%	98.9%	2.17E-84	3.95E-80
5	LYZ	Paneth cells	0.7995394	99.7%	96.3%	5.79E-59	1.05E-54
5	AHR	Microfold cells	0.7662859	59.7%	33.9%	1.54E-51	2.80E-47
5	GSTM3	Enterocytes	0.7417366	83.5%	61.3%	1.53E-54	2.78E-50
5	TULP4	Microfold cells	0.7068923	49.2%	18.2%	1.47E-99	2.68E-95
5	PLA2G10	Goblet cells	0.7059944	73.4%	47.5%	2.86E-56	5.21E-52
5	KLF6	Tuft cells	0.7034208	80.2%	57.0%	6.92E-42	1.26E-37
5	CXCL16	Microfold cells	0.6828763	79.8%	51.7%	5.18E-71	9.41E-67
5	CDH17	Enterocytes	0.6528447	73.4%	57.8%	2.51E-27	4.57E-23
5	PPP1R14D	Enterocytes	0.6252819	72.4%	58.6%	9.33E-27	1.70E-22
5	EHF	Enterocytes	0.6164408	48.1%	31.6%	2.57E-25	4.67E-21
5	CTSD	Microfold cells	0.6138042	96.7%	83.9%	1.76E-40	3.20E-36
5	CTSH	Microfold cells	0.5982448	60.8%	39.3%	1.11E-38	2.03E-34
5	VNN1	Enterocytes	0.5971123	43.3%	29.1%	2.44E-21	4.44E-17
5	ACSL5	Enterocytes	0.5777376	65.8%	41.0%	1.15E-51	2.09E-47
5	PROM1	Crypt cells	0.5736268	48.4%	29.0%	6.30E-33	1.15E-28
5	GULP1	Enterocytes	0.5715908	33.8%	16.3%	7.17E-37	1.30E-32
5	BACE2	Goblet cells	0.5713216	82.0%	62.4%	2.82E-40	5.12E-36
5	GPX2	Paneth cells	0.5567288	97.3%	91.6%	8.31E-46	1.51E-41
5	SLC40A1	Enterocytes	0.5515893	83.5%	68.5%	5.28E-23	9.60E-19
5	MFGE8	Microfold cells	0.5469483	36.8%	10.2%	7.97E-105	1.45E-100
5	CREB3L1	Goblet cells	0.5437355	69.3%	42.7%	1.57E-51	2.85E-47
5	PLIN3	Enterocytes	0.5197411	63.2%	41.4%	1.95E-40	3.54E-36
5	CFB	Paneth cells	0.4932686	66.3%	56.5%	1.47E-11	2.67E-07
5	TMPRSS2	Microfold cells	0.4854017	78.8%	57.6%	6.45E-34	1.17E-29
5	PHGR1	Goblet cells	0.4774958	95.9%	95.5%	1.68E-13	3.06E-09
5	GLS	Enterocytes	0.4730555	44.0%	22.6%	1.45E-41	2.63E-37
5	IL13RA1	Tuft cells	0.4547128	49.2%	29.6%	9.38E-32	1.70E-27
5	TRNP1	Enteroendocrine cells	0.4481098	79.6%	52.0%	3.24E-49	5.89E-45
5	EPHB2	Crypt cells	0.4382521	31.4%	9.0%	3.42E-83	6.22E-79
5	NFIC	Microfold cells	0.4106828	40.0%	16.8%	1.64E-56	2.97E-52

5	KLF5	Enterocytes	0.3918638	74.5%	58.7%	4.52E-17	8.23E-13
5	TMC4	Enterocytes	0.38373	66.8%	47.2%	7.01E-28	1.27E-23
5	ALCAM	Enteroendocrine cells	0.370132	35.6%	21.3%	4.36E-21	7.94E-17
5	ATG16L1	Paneth cells	0.3609958	33.8%	13.0%	1.25E-55	2.26E-51
5	BMI1	Crypt cells	0.3590221	27.2%	10.6%	3.72E-43	6.76E-39
5	LGR4	Paneth cells	0.3570892	42.7%	27.3%	1.51E-21	2.74E-17
5	RAC2	Microfold cells	0.3558436	48.5%	28.6%	8.47E-32	1.54E-27
5	MAOA	Enterocytes	0.3423954	38.3%	21.6%	3.68E-27	6.69E-23
5	HNF4A	Enterocytes	0.3140995	41.6%	26.2%	1.69E-21	3.08E-17
5	AGR2	Paneth cells	0.3085367	94.9%	90.5%	1.34E-04	1.00E+00
5	RIPK2	Paneth cells	0.3024056	32.2%	15.2%	2.18E-33	3.97E-29
5	CBR1	Enterocytes	0.2915066	61.7%	45.5%	4.15E-18	7.54E-14
5	FLOT2	Enterocytes	0.2876335	34.8%	15.0%	1.90E-44	3.45E-40
5	ALOX5	Tuft cells	0.2817476	39.5%	25.1%	2.54E-19	4.61E-15
5	MANF	Goblet cells	0.2768123	48.1%	35.6%	6.00E-13	1.09E-08
5	RAP1A	Paneth cells	0.2737808	40.2%	25.9%	2.22E-18	4.05E-14
5	CASP6	Enterocytes	0.2734154	33.9%	20.8%	3.55E-19	6.45E-15
5	ELF3	Enterocytes	0.270675	93.2%	82.6%	2.52E-11	4.58E-07
5	IRF6	Microfold cells	0.2623737	39.9%	26.2%	1.82E-16	3.32E-12
7	**MUC4**	**Goblet cells**	1.222235	68.7%	24.2%	5.79E-138	1.05E-133
7	ONECUT2	Microfold cells	1.0860054	71.6%	29.3%	4.86E-122	8.84E-118
7	KLF6	Tuft cells	1.0477885	90.4%	56.9%	6.71E-97	1.22E-92
7	TNFAIP2	Microfold cells	0.9916834	89.6%	65.6%	9.57E-69	1.74E-64
7	KLF5	Enterocytes	0.8291456	89.1%	58.3%	1.31E-80	2.38E-76
7	GLS	Enterocytes	0.7959174	62.3%	22.1%	5.69E-114	1.04E-109
7	FOSL2	Microfold cells	0.7602482	70.8%	31.7%	1.94E-88	3.52E-84
7	AHR	Microfold cells	0.7588435	70.1%	33.8%	1.94E-80	3.52E-76
7	VEGFA	Enteroendocrine cells	0.7584788	80.4%	49.9%	2.80E-66	5.09E-62
7	ELF3	Enterocytes	0.7556588	97.9%	82.5%	8.78E-84	1.60E-79
7	TMPRSS2	Microfold cells	0.7390017	83.4%	57.7%	1.65E-53	2.99E-49
7	KLF3	Tuft cells	0.7061643	81.5%	45.7%	1.13E-80	2.06E-76
7	CTNNB1	Paneth cells	0.6802778	79.5%	45.5%	1.75E-69	3.18E-65
7	CD55	Enterocytes	0.6333278	91.9%	68.9%	1.01E-60	1.84E-56
7	SOX9	Tuft cells	0.6139187	71.2%	47.3%	5.08E-37	9.23E-33

7	MUC13	Goblet cells	0.6087357	82.5%	56.9%	3.17E-42	5.76E-38
7	CFB	Paneth cells	0.6041751	80.0%	56.0%	3.11E-44	5.66E-40
7	SLC11A2	Enterocytes	0.5866516	62.3%	38.9%	6.10E-36	1.11E-31
7	ALCAM	Enteroendocrine cells	0.5731076	49.7%	20.8%	3.22E-59	5.85E-55
7	PROM1	Crypt cells	0.5640026	57.8%	28.9%	2.45E-50	4.45E-46
7	EHF	Enterocytes	0.5561717	61.6%	31.2%	2.77E-52	5.04E-48
7	IL4R	Paneth cells	0.510359	46.9%	19.1%	6.11E-59	1.11E-54
7	OLFM4	Crypt cells	0.4970924	87.6%	65.0%	3.27E-25	5.95E-21
7	CEACAM1	Enterocytes	0.4901348	68.9%	46.2%	6.59E-28	1.20E-23
7	SLC15A1	Enterocytes	0.4816837	38.2%	18.2%	2.43E-34	4.42E-30
7	LGR4	Paneth cells	0.4691746	53.9%	27.0%	4.64E-46	8.43E-42
7	VNN1	Enterocytes	0.459369	55.7%	28.8%	3.82E-45	6.95E-41
7	IL13RA1	Tuft cells	0.4233827	53.9%	29.7%	4.63E-35	8.41E-31
7	DMBT1	Enterocytes	0.4227728	58.8%	47.9%	4.49E-06	8.16E-02
7	TULP4	Microfold cells	0.4191586	43.9%	19.0%	4.86E-46	8.83E-42
7	PMEPA1	Enterocytes	0.4182813	77.0%	53.9%	1.20E-31	2.19E-27
7	NFIB	Microfold cells	0.4167072	49.5%	22.2%	1.11E-47	2.03E-43
7	SLC40A1	Enterocytes	0.3365306	84.7%	68.7%	3.80E-23	6.92E-19
7	NR5A2	Enterocytes	0.3332789	39.4%	18.6%	3.81E-34	6.92E-30
7	HNF4A	Enterocytes	0.3066401	46.3%	26.2%	7.56E-25	1.38E-20
7	SLC12A2	Crypt cells	0.3014612	50.8%	36.1%	5.98E-12	1.09E-07
7	TMEM45B	Enterocytes	0.2989397	87.0%	66.3%	1.86E-25	3.38E-21
7	CTSD	Microfold cells	0.2915682	95.3%	84.1%	1.92E-20	3.48E-16
7	TMC4	Enterocytes	0.2816079	68.9%	47.4%	1.58E-22	2.87E-18
7	CDH17	Enterocytes	0.2672011	72.3%	58.1%	3.12E-13	5.68E-09
7	PLA2G2A	Paneth cells	0.2633057	33.0%	14.3%	2.60E-30	4.72E-26
7	GALNT12	Goblet cells	0.2511636	38.2%	20.0%	9.97E-24	1.81E-19
8	PHGR1	Enterocytes	2.5436771	99.6%	95.4%	2.01E-164	3.65E-160
8	KRT20	Enterocytes	2.408736	84.2%	31.5%	3.15E-210	5.72E-206
8	FABP2	Enterocytes	2.3939266	84.8%	28.5%	1.73E-227	3.15E-223
8	REG4	Enterochromaffin cells	2.2839268	93.8%	76.7%	4.46E-109	8.11E-105
8	ALDOB	Enterocytes	2.2734611	90.5%	37.9%	1.34E-213	2.44E-209
8	RBP2	Enterocytes	2.2042808	55.6%	18.5%	5.98E-107	1.09E-102
8	FABP1	Enterocytes	1.7882132	99.8%	94.5%	2.73E-131	4.97E-127

8	PRAP1	Enterocytes	1.7305408	95.1%	68.8%	9.08E-123	1.65E-118
8	GSTA1	Enterocytes	1.6873732	59.9%	12.8%	2.08E-203	3.78E-199
8	ANPEP	Enterocytes	1.644009	38.3%	12.1%	1.76E-70	3.20E-66
8	**SI**	**Enterocytes**	1.6205421	60.5%	12.9%	3.78E-205	6.87E-201
8	AGR2	Goblet cells	1.5945104	98.4%	90.5%	2.28E-111	4.15E-107
8	MTTP	Enterocytes	1.5785546	68.7%	15.2%	4.46E-240	8.10E-236
8	TM4SF5	Enterocytes	1.5236956	81.7%	29.2%	5.53E-187	1.01E-182
8	CBR1	Enterocytes	1.4168701	90.9%	44.5%	5.55E-153	1.01E-148
8	OLFM4	Crypt cells	1.3659362	79.2%	65.5%	2.28E-25	4.15E-21
8	TM4SF20	Paneth cells	1.3612555	83.7%	55.3%	1.15E-57	2.08E-53
8	LYZ	Paneth cells	1.3397759	100.0%	96.3%	4.40E-94	8.01E-90
8	SLC12A2	Crypt cells	1.1517261	56.0%	35.9%	8.67E-32	1.58E-27
8	DGAT1	Enterocytes	1.1489495	83.1%	40.3%	1.01E-126	1.83E-122
8	DMBT1	Enterocytes	1.130379	49.8%	48.4%	1.31E-05	2.38E-01
8	MUC13	Enterocytes	1.1214093	91.2%	56.6%	3.25E-93	5.90E-89
8	SLC5A1	Enterocytes	1.1117776	76.1%	25.4%	1.44E-160	2.63E-156
8	PLA2G10	Goblet cells	1.1088908	84.2%	47.6%	5.12E-103	9.31E-99
8	CDH17	Enterocytes	1.1047202	91.4%	57.3%	2.40E-101	4.36E-97
8	**VIL1**	**Enterocytes**	1.0957501	85.6%	46.1%	3.64E-112	6.61E-108
8	FUOM	Enterocytes	1.0328843	92.8%	61.2%	9.54E-108	1.74E-103
8	CDC42EP5	Enterocytes	1.0314011	100.0%	86.1%	1.89E-109	3.44E-105
8	CYBA	Microfold cells	0.9629255	99.4%	91.2%	3.66E-86	6.65E-82
8	MEP1A	Enterocytes	0.9537389	49.8%	8.6%	1.82E-195	3.31E-191
8	TFF3	Goblet cells	0.9279267	99.4%	99.0%	3.38E-31	6.14E-27
8	CD24	Paneth cells	0.8916264	94.7%	90.5%	2.50E-45	4.54E-41
8	TM6SF2	Enterocytes	0.8700119	59.5%	12.8%	1.92E-192	3.50E-188
8	CLDN15	Enterocytes	0.8453627	55.1%	21.0%	2.05E-84	3.73E-80
8	PPP1R14D	Enterocytes	0.844764	93.0%	58.0%	4.63E-97	8.41E-93
8	SLC40A1	Enterocytes	0.840645	89.3%	68.5%	2.16E-49	3.92E-45
8	ACSL5	Enterocytes	0.8066853	76.7%	41.0%	2.11E-77	3.83E-73
8	SLC39A5	Enterocytes	0.8031998	61.7%	16.4%	2.75E-153	5.01E-149
8	PRR15L	Enterocytes	0.8002476	84.2%	50.8%	1.89E-76	3.44E-72
8	KCNE3	Crypt cells	0.767786	61.5%	35.3%	9.02E-43	1.64E-38
8	CTSD	Microfold cells	0.7491277	98.6%	84.0%	1.63E-71	2.96E-67

8	KHK	Enterocytes	0.7482688	30.7%	8.3%	4.60E-66	8.37E-62
8	CDX1	Enterocytes	0.7167067	64.0%	23.1%	1.74E-101	3.16E-97
8	CTSH	Microfold cells	0.7125573	73.9%	39.1%	2.65E-69	4.83E-65
8	SLC51B	Enterocytes	0.7059789	31.1%	3.0%	9.00E-201	1.64E-196
8	CDX2	Enterocytes	0.6904999	56.8%	25.5%	5.23E-64	9.51E-60
8	MYO1A	Enterocytes	0.6709348	43.0%	8.7%	3.78E-138	6.87E-134
8	ISX	Enterocytes	0.6373578	42.4%	7.8%	2.40E-151	4.37E-147
8	OTC	Enterocytes	0.6148372	29.6%	2.3%	1.33E-231	2.42E-227
8	TMEM45B	Enterocytes	0.6103095	90.9%	66.2%	1.84E-50	3.35E-46
8	EPHX2	Enterocytes	0.603544	42.2%	9.1%	1.23E-124	2.23E-120
8	MARCKSL1	Microfold cells	0.5911924	87.7%	70.7%	5.90E-36	1.07E-31
8	CYBRD1	Enterocytes	0.5892586	26.1%	6.1%	1.63E-67	2.96E-63
8	MOGAT2	Enterocytes	0.5802543	38.5%	9.8%	1.41E-92	2.57E-88
8	CTNNB1	Paneth cells	0.5772495	71.2%	46.0%	4.57E-31	8.31E-27
8	CREB3L1	Goblet cells	0.5753941	67.9%	43.3%	2.59E-38	4.72E-34
8	CPS1	Enterocytes	0.5744862	30.5%	3.2%	2.31E-185	4.21E-181
8	KLF6	Tuft cells	0.5713265	84.2%	57.3%	1.07E-41	1.94E-37
8	TRNP1	Enteroendocrine cells	0.5705205	72.2%	52.9%	4.82E-29	8.77E-25
8	RNF186	Enterocytes	0.5560172	48.8%	31.0%	9.13E-21	1.66E-16
8	VNN1	Enterocytes	0.5559764	50.2%	29.1%	1.97E-28	3.58E-24
8	CDHR2	Tuft cells	0.5555172	40.3%	12.0%	1.06E-75	1.94E-71
8	RAP1A	Paneth cells	0.5443644	58.2%	25.4%	5.37E-64	9.77E-60
8	GPX2	Paneth cells	0.5387191	90.5%	92.0%	3.99E-18	7.25E-14
8	GJB1	Enterochromaffin cells	0.5374033	51.4%	20.4%	6.54E-67	1.19E-62
8	GSTM3	Enterocytes	0.5280689	83.1%	61.8%	5.42E-34	9.85E-30
8	SLC11A2	Enterocytes	0.5264476	57.2%	39.2%	3.61E-19	6.56E-15
8	SLC51A	Enterocytes	0.5221982	29.4%	9.2%	4.08E-51	7.42E-47
8	CXCL16	Microfold cells	0.5175707	82.7%	52.1%	3.81E-48	6.92E-44
8	PDHA1	Enterocytes	0.5147105	57.2%	26.1%	4.17E-56	7.58E-52
8	NPC1L1	Enterocytes	0.487783	51.4%	22.9%	4.60E-52	8.37E-48
8	APOA1	Enterocytes	0.4819974	25.7%	17.7%	6.85E-07	1.25E-02
8	HNF4A	Enterocytes	0.4752325	50.4%	26.1%	4.86E-36	8.83E-32
8	ABCG2	Enterocytes	0.4446536	25.7%	3.2%	7.59E-131	1.38E-126
8	GLS	Enterocytes	0.4374502	44.7%	23.0%	8.20E-29	1.49E-24

8	KRTCAP3	Enterocytes	0.4352378	81.9%	55.7%	5.61E-35	1.02E-30
8	MANF	Goblet cells	0.4176367	57.4%	35.4%	5.76E-27	1.05E-22
8	DEGS2	Enterocytes	0.4067052	38.5%	16.2%	9.02E-40	1.64E-35
8	ABHD11	Enterocytes	0.4062454	42.8%	21.7%	1.53E-30	2.79E-26
8	ELF3	Enterocytes	0.4009566	95.3%	82.7%	3.03E-28	5.52E-24
8	KLF5	Enterocytes	0.396615	82.7%	58.7%	8.59E-27	1.56E-22
8	APOBEC1	Enterocytes	0.3955206	27.6%	8.1%	1.94E-51	3.53E-47
8	LGR4	Paneth cells	0.3852105	50.0%	27.3%	5.48E-29	9.96E-25
8	CCL20	Microfold cells	0.3823222	34.6%	29.5%	2.17E-03	1.00E+00
8	FAHD1	Enterocytes	0.3821849	37.7%	16.9%	7.90E-35	1.44E-30
8	CASP6	Enterocytes	0.3817683	43.0%	20.6%	6.53E-34	1.19E-29
8	SDSL	Enterocytes	0.3814496	29.8%	8.3%	2.29E-60	4.16E-56
8	HMOX1	Enterocytes	0.3680304	31.5%	14.0%	3.00E-28	5.46E-24
8	TMC4	Enterocytes	0.3678626	70.4%	47.5%	1.27E-26	2.30E-22
8	FABP5	Enteroendocrine cells	0.3547392	41.6%	26.1%	6.67E-16	1.21E-11
8	CD55	Enterocytes	0.3147977	84.0%	69.4%	8.18E-16	1.49E-11
8	NUCB2	Enteroendocrine cells	0.312254	36.8%	20.7%	1.10E-18	2.00E-14
8	PLIN3	Enterocytes	0.3040705	56.0%	42.2%	7.93E-14	1.44E-09
8	CFB	Paneth cells	0.301982	67.9%	56.6%	3.42E-09	6.22E-05
8	RAC2	Microfold cells	0.3015192	47.1%	29.1%	3.39E-18	6.17E-14
8	NFIB	Microfold cells	0.2995167	39.7%	22.8%	5.58E-18	1.01E-13
8	NR5A2	Enterocytes	0.2985843	37.4%	18.7%	1.74E-25	3.16E-21
8	AHR	Microfold cells	0.2923789	53.3%	34.7%	2.70E-17	4.91E-13
8	NR1H4	Enterocytes	0.2823862	25.5%	8.0%	4.18E-41	7.60E-37
8	ESPN	Tuft cells	0.272884	26.5%	10.5%	1.14E-28	2.07E-24
8	SOX9	Tuft cells	0.2687947	64.2%	47.7%	3.04E-12	5.53E-08
8	DPP4	Enterocytes	0.2580845	25.1%	10.7%	2.35E-23	4.27E-19
9	PLA2G2A	Paneth cells	0.2597873	29.5%	14.6%	2.71E-14	4.92E-10
10	MKI67	Proliferating cells	2.0368014	85.3%	3.2%	0.00E+00	0.00E+00
10	FABP5	Enteroendocrine cells	1.5901609	80.7%	25.1%	3.68E-162	6.68E-158
10	GPX2	Paneth cells	1.0215553	99.4%	91.7%	3.47E-63	6.30E-59
10	KRTCAP3	Enterocytes	0.9920984	91.5%	55.7%	2.34E-76	4.25E-72
10	AGR2	Goblet cells	0.926293	99.4%	90.5%	2.03E-55	3.69E-51

10	GSTM3	Enterocytes	0.7703505	92.6%	61.8%	3.09E-55	5.63E-51
10	REG4	Enterochromaffin cells	0.7478527	92.1%	76.9%	1.22E-37	2.22E-33
10	CYBA	Microfold cells	0.7277815	100.0%	91.3%	1.73E-57	3.15E-53
10	KCNE3	Crypt cells	0.5878441	70.8%	35.3%	1.77E-48	3.23E-44
10	PDHA1	Enterocytes	0.5765646	61.5%	26.3%	3.29E-53	5.99E-49
10	PRSS1	Paneth cells	0.5340767	36.0%	18.6%	1.02E-16	1.85E-12
10	LYZ	Paneth cells	0.524251	100.0%	96.4%	1.58E-28	2.87E-24
10	PRSS2	Enterocytes	0.5225928	35.7%	22.9%	2.35E-08	4.27E-04
10	ANXA5	Microfold cells	0.5213797	95.5%	71.3%	1.25E-45	2.28E-41
10	MANF	Goblet cells	0.5043534	65.7%	35.4%	1.91E-35	3.47E-31
10	CBR1	Enterocytes	0.499504	75.1%	45.6%	6.65E-33	1.21E-28
10	MARCKSL1	Microfold cells	0.4706219	94.3%	70.7%	5.25E-32	9.54E-28
10	VIL1	Enterocytes	0.4549234	74.5%	47.0%	1.04E-27	1.89E-23
10	CDX2	Goblet cells	0.4330837	50.7%	26.1%	3.18E-27	5.78E-23
10	FERMT1	Enterocytes	0.4306654	70.5%	43.5%	1.43E-26	2.59E-22
10	FUOM	Enterocytes	0.4080464	85.0%	61.9%	1.35E-21	2.45E-17
10	CASP6	Enterocytes	0.40656	44.2%	20.9%	2.24E-28	4.08E-24
10	CCK	Enteroendocrine cells	0.3939813	44.2%	26.9%	1.80E-13	3.28E-09
10	PRR15L	Enterocytes	0.3938123	71.7%	51.6%	1.59E-16	2.89E-12
10	CTSH	Microfold cells	0.3823203	62.6%	39.9%	9.94E-21	1.81E-16
10	CDC42EP5	Enterocytes	0.3561452	98.3%	86.4%	3.47E-15	6.31E-11
10	ABHD11	Enterocytes	0.3214443	41.6%	22.0%	2.49E-19	4.52E-15
10	KLK1	Goblet cells	0.307423	28.0%	10.5%	2.16E-25	3.93E-21
10	RAP1A	Paneth cells	0.2977624	46.5%	26.2%	6.15E-18	1.12E-13
10	SLC12A2	Crypt cells	0.2959854	60.6%	36.0%	3.06E-19	5.56E-15
10	FABP1	Enterocytes	0.2775483	96.3%	94.7%	2.88E-11	5.25E-07
10	ACSL5	Enterocytes	0.2716839	66.0%	41.7%	2.07E-18	3.76E-14
10	GALNT12	Goblet cells	0.2699027	36.5%	20.3%	1.62E-13	2.94E-09
10	GATA4	Enterocytes	0.2534376	31.4%	13.6%	1.44E-21	2.62E-17
11	APOC3	Enterocytes	5.7566624	77.8%	16.4%	5.01E-226	9.11E-222
11	APOA1	Enterocytes	5.6963616	81.4%	16.3%	3.81E-251	6.92E-247
11	APOA4	Enterocytes	5.2452214	71.2%	9.8%	2.35E-288	4.27E-284
11	RBP2	Enterocytes	4.7208414	89.5%	18.1%	3.16E-283	5.75E-279
11	ANPEP	Enterocytes	3.5682527	88.9%	11.1%	0.00E+00	0.00E+00

11	ALDOB	Enterocytes	3.1952812	98.4%	38.5%	6.06E-209	1.10E-204
11	FABP2	Enterocytes	2.8472746	95.1%	29.1%	7.56E-213	1.38E-208
11	APOB	Enterocytes	2.6686397	70.6%	4.4%	0.00E+00	0.00E+00
11	PRAP1	Enterocytes	2.5659005	99.0%	69.1%	2.42E-147	4.41E-143
11	**SI**	**Enterocytes**	2.524305	82.4%	13.1%	7.05E-288	1.28E-283
11	GSTA1	Enterocytes	2.2120824	62.4%	13.5%	8.76E-146	1.59E-141
11	CYP3A4	Enterocytes	2.185416	64.7%	5.4%	0.00E+00	0.00E+00
11	PHGR1	Enterocytes	2.1045386	100.0%	95.4%	7.09E-123	1.29E-118
11	TM4SF5	Enterocytes	2.011504	89.5%	29.8%	1.71E-178	3.11E-174
11	MTTP	Enterocytes	1.7889031	76.8%	15.8%	4.18E-193	7.60E-189
11	MEP1A	Enterocytes	1.6463815	81.7%	8.3%	0.00E+00	0.00E+00
11	KHK	Enterocytes	1.6096191	65.0%	7.7%	2.16E-280	3.94E-276
11	SLC5A1	Enterocytes	1.6028598	85.6%	26.0%	5.82E-158	1.06E-153
11	DGAT1	Enterocytes	1.2809376	88.9%	40.8%	3.66E-103	6.66E-99
11	CBR1	Enterocytes	1.2646486	79.1%	45.6%	2.49E-58	4.52E-54
11	KRT20	Enterocytes	1.2379269	87.6%	32.3%	7.83E-106	1.42E-101
11	SLC2A5	Enterocytes	1.175924	43.1%	1.3%	0.00E+00	0.00E+00
11	ZG16	Goblet cells	1.0747598	52.0%	1.9%	0.00E+00	0.00E+00
11	MUC13	Enterocytes	1.0669953	90.5%	57.2%	1.71E-58	3.11E-54
11	MYO1A	Enterocytes	1.0639568	62.7%	8.7%	1.86E-221	3.38E-217
11	ACSL5	Enterocytes	0.9656912	77.1%	41.5%	2.24E-53	4.07E-49
11	CDHR2	Tuft cells	0.9474572	59.8%	12.0%	2.39E-142	4.34E-138
11	CREB3L3	Enterocytes	0.9159887	44.4%	2.1%	0.00E+00	0.00E+00
11	SLC51B	Enterocytes	0.8957053	50.7%	3.0%	0.00E+00	0.00E+00
11	SLC39A5	Enterocytes	0.8591075	62.1%	17.2%	6.35E-101	1.15E-96
11	ALPI	Enterocytes	0.8311837	41.8%	1.5%	0.00E+00	0.00E+00
11	TM4SF20	Paneth cells	0.8196883	87.9%	55.7%	2.46E-35	4.47E-31
11	CYBRD1	Enterocytes	0.8112268	41.2%	6.0%	2.97E-128	5.40E-124
11	DPP4	Enterocytes	0.8067993	48.4%	10.3%	2.11E-103	3.83E-99
11	ABCG2	Enterocytes	0.788737	41.5%	3.2%	3.09E-243	5.63E-239
11	FABP1	Enterocytes	0.7830006	100.0%	94.6%	1.03E-40	1.88E-36
11	RNF186	Enterocytes	0.7808505	69.3%	30.7%	2.66E-51	4.83E-47
11	MOGAT2	Enterocytes	0.7700609	54.2%	9.8%	4.66E-140	8.48E-136
11	TM6SF2	Enterocytes	0.752264	57.5%	13.6%	1.17E-107	2.14E-103

11	SLC15A1	Enterocytes	0.7443247	57.8%	18.1%	5.17E-75	9.40E-71
11	**VIL1**	**Enterocytes**	0.7238992	78.1%	47.0%	5.53E-37	1.01E-32
11	APOBEC1	Enterocytes	0.7056592	45.1%	7.9%	3.11E-116	5.65E-112
11	AQP11	Enterocytes	0.6941777	39.2%	5.1%	1.46E-140	2.65E-136
11	SLC51A	Enterocytes	0.6177331	44.4%	9.1%	7.39E-94	1.34E-89
11	TREH	Enterocytes	0.6009434	35.6%	2.3%	1.39E-234	2.53E-230
11	EPHX2	Enterocytes	0.5904811	43.1%	9.7%	1.39E-82	2.52E-78
11	NPC1L1	Enterocytes	0.5716448	52.0%	23.4%	3.30E-35	6.01E-31
11	CLDN15	Enterocytes	0.5567555	51.6%	21.7%	9.87E-38	1.79E-33
11	SLC28A1	Enterocytes	0.5539743	32.0%	0.7%	0.00E+00	0.00E+00
11	CTSD	Microfold cells	0.522439	98.7%	84.3%	1.89E-29	3.44E-25
11	ABCC2	Enterocytes	0.5141934	28.4%	2.5%	9.94E-144	1.81E-139
11	PLIN2	Enterocytes	0.5070213	35.3%	10.2%	1.74E-46	3.16E-42
11	FUOM	Enterocytes	0.4927881	86.9%	61.9%	1.62E-23	2.95E-19
11	PDZK1	Enterocytes	0.4899581	33.3%	3.4%	2.20E-148	4.00E-144
11	ESPN	Tuft cells	0.453329	39.9%	10.4%	2.16E-59	3.92E-55
11	AQP7	Enterocytes	0.4405109	29.1%	1.7%	1.16E-204	2.12E-200
11	DMBT1	Enterocytes	0.4081332	33.3%	48.9%	7.53E-03	1.00E+00
11	HMOX1	Enterocytes	0.3617841	37.6%	14.1%	7.39E-31	1.34E-26
11	SLC11A2	Enterocytes	0.3440392	54.2%	39.6%	1.98E-08	3.60E-04
11	ISX	Enterocytes	0.3406397	30.1%	8.7%	2.43E-37	4.42E-33
11	MAOA	Enterocytes	0.2961257	36.6%	22.3%	6.20E-10	1.13E-05
12	APOC3	Enterocytes	2.6740208	77.6%	16.6%	2.10E-183	3.81E-179
12	APOA1	Enterocytes	2.6713295	77.2%	16.6%	4.45E-181	8.08E-177
12	RBP2	Enterocytes	2.239862	90.7%	18.3%	1.38E-230	2.51E-226
12	APOA4	Enterocytes	2.1560274	63.8%	10.2%	4.83E-182	8.79E-178
12	ANPEP	Enterocytes	1.642971	80.2%	11.6%	5.15E-248	9.36E-244
12	PRAP1	Enterocytes	1.0070679	92.5%	69.4%	5.09E-46	9.25E-42
12	ALDOB	Enterocytes	0.8902022	87.3%	39.0%	8.93E-76	1.62E-71
12	CYP3A4	Enterocytes	0.6723362	48.9%	6.0%	1.20E-160	2.18E-156
12	TM4SF5	Enterocytes	0.583481	68.7%	30.5%	7.95E-44	1.45E-39
12	GSTA1	Enterocytes	0.5817792	44.0%	14.1%	8.83E-44	1.61E-39
12	APOB	Enterocytes	0.5356857	44.8%	5.3%	3.59E-151	6.53E-147
12	FABP2	Enterocytes	0.5107734	73.1%	29.9%	1.53E-53	2.79E-49

12	PHGR1	Enterocytes	0.4890928	100.0%	95.4%	2.52E-40	4.59E-36
12	KHK	Enterocytes	0.4697529	42.5%	8.5%	4.16E-79	7.57E-75
12	**SI**	**Enterocytes**	0.4208177	44.4%	14.3%	1.06E-41	1.93E-37
12	CDHR2	Tuft cells	0.2933289	29.5%	12.9%	6.59E-16	1.20E-11
12	MYO1A	Enterocytes	0.2719944	28.0%	9.7%	1.72E-22	3.13E-18
12	MEP1A	Enterocytes	0.2556866	30.6%	9.8%	6.08E-27	1.11E-22
14	PMEPA1	Enterocytes	1.1535249	97.4%	54.1%	9.23E-66	1.68E-61
14	KRT7	Goblet cells	1.0773666	100.0%	90.4%	2.28E-57	4.15E-53
14	TNFAIP2	Microfold cells	0.5895659	86.4%	66.3%	5.70E-18	1.04E-13
14	MFGE8	Microfold cells	0.4682454	37.3%	11.3%	2.16E-34	3.92E-30
14	COL18A1	Enterocytes	0.4197152	39.0%	14.5%	6.55E-25	1.19E-20
14	KLF6	Tuft cells	0.3335969	77.2%	58.1%	3.47E-07	6.32E-03
14	CTSD	Microfold cells	0.3169752	96.1%	84.4%	1.90E-08	3.46E-04
14	MAOA	Enterocytes	0.302542	42.1%	22.2%	6.51E-13	1.18E-08
14	ALCAM	Enteroendocrine cells	0.2861744	31.1%	22.0%	2.91E-04	1.00E+00
14	PROM1	Crypt cells	0.2748418	42.1%	30.0%	2.12E-05	3.86E-01
14	FERMT1	Enterocytes	0.274835	65.8%	43.9%	1.05E-09	1.91E-05
14	CFB	Paneth cells	0.2581828	68.4%	56.9%	3.62E-04	1.00E+00
14	MUC4	Goblet cells	0.2514177	38.6%	26.0%	6.55E-05	1.00E+00
15	CCL20	Microfold cells	2.3042834	63.6%	29.2%	2.26E-31	4.12E-27
15	MUC13	Goblet cells	1.7396875	76.9%	57.8%	1.30E-18	2.37E-14
15	TM4SF20	Paneth cells	1.7173912	65.9%	56.4%	3.56E-10	6.48E-06
15	TMPRSS2	Microfold cells	1.3799495	89.6%	58.5%	2.67E-43	4.85E-39
15	CXCL16	Microfold cells	1.3496596	94.8%	52.8%	1.73E-61	3.15E-57
15	KRT20	Goblet cells	1.3120793	54.9%	33.5%	4.07E-14	7.41E-10
15	CD55	Enterocytes	1.3075549	87.9%	69.7%	1.63E-20	2.97E-16
15	MARCKSL1	Microfold cells	1.258107	98.3%	71.0%	5.22E-45	9.49E-41
15	MMP7	Paneth cells	1.2562385	90.8%	76.6%	5.26E-19	9.56E-15
15	TNFAIP2	Microfold cells	1.2180817	91.3%	66.4%	9.24E-42	1.68E-37
15	KLF6	Tuft cells	0.9506938	90.8%	58.0%	1.50E-32	2.73E-28
15	CDC42EP5	Enterocytes	0.9305525	99.4%	86.5%	2.12E-33	3.86E-29
15	CFB	Paneth cells	0.90961	86.7%	56.7%	2.29E-24	4.17E-20
15	SLC5A1	Enterocytes	0.8722516	54.9%	27.2%	1.18E-20	2.15E-16

15	ELF3	Enterocytes	0.7545213	97.7%	83.0%	2.81E-19	5.11E-15
15	GULP1	Enterocytes	0.7456127	43.9%	17.0%	1.47E-23	2.68E-19
15	ABHD11	Enterocytes	0.7278028	60.1%	22.0%	3.69E-38	6.71E-34
15	RELB	Microfold cells	0.6983259	62.4%	21.7%	1.63E-44	2.96E-40
15	CYBA	Microfold cells	0.6900823	99.4%	91.4%	3.99E-19	7.25E-15
15	VNN1	Enterocytes	0.6686265	54.3%	29.6%	6.20E-16	1.13E-11
15	IRF6	Microfold cells	0.6629595	61.3%	26.6%	1.06E-26	1.92E-22
15	SLC12A2	Crypt cells	0.6118334	58.4%	36.4%	3.01E-12	5.47E-08
15	CLDN15	Enterocytes	0.5962495	37.6%	22.3%	1.28E-07	2.33E-03
15	ALDOB	Enterocytes	0.5530126	60.7%	39.8%	1.58E-10	2.88E-06
15	KRT7	Goblet cells	0.5529729	97.7%	90.5%	1.77E-12	3.23E-08
15	KLF5	Enterocytes	0.525437	81.5%	59.4%	1.45E-13	2.64E-09
15	RIPK2	Paneth cells	0.5131565	43.9%	15.8%	4.52E-24	8.21E-20
15	KLF3	Tuft cells	0.5096692	72.8%	47.0%	5.75E-15	1.05E-10
15	FERMT1	Enterocytes	0.5092051	63.6%	44.0%	4.39E-09	7.98E-05
15	TM4SF5	Enterocytes	0.4702122	48.6%	31.2%	1.97E-07	3.59E-03
15	SOX9	Tuft cells	0.4654268	68.8%	48.1%	1.17E-09	2.12E-05
15	GLS	Enterocytes	0.4597126	48.6%	23.6%	5.59E-15	1.02E-10
15	CTNNB1	Paneth cells	0.4514236	76.9%	46.7%	4.74E-18	8.63E-14
15	MUC4	Goblet cells	0.4481927	46.2%	26.0%	1.23E-09	2.23E-05
15	CDX1	Enterocytes	0.4406712	47.4%	24.5%	8.15E-13	1.48E-08
15	CD24	Paneth cells	0.4129769	97.7%	90.6%	2.31E-15	4.20E-11
15	BACE2	Goblet cells	0.3933818	86.1%	63.3%	4.16E-11	7.57E-07
15	ALCAM	Enteroendocrine cells	0.384968	40.5%	21.9%	2.29E-09	4.17E-05
15	PRAP1	Enterocytes	0.3445322	79.2%	69.8%	2.24E-04	1.00E+00
15	MIER3	Microfold cells	0.3434085	25.4%	12.0%	2.04E-08	3.70E-04
15	TMEM45B	Enterocytes	0.3372559	77.5%	67.1%	1.04E-05	1.89E-01
15	PPP1R14D	Enterocytes	0.3355069	74.6%	59.2%	1.06E-05	1.93E-01
15	KCNE3	Crypt cells	0.3216387	45.7%	36.3%	7.44E-04	1.00E+00
15	LYZ	Paneth cells	0.3143866	99.4%	96.4%	1.45E-08	2.63E-04
15	GJB2	Microfold cells	0.2785151	28.3%	12.6%	3.79E-10	6.89E-06
15	ACSL5	Enterocytes	0.2730936	63.6%	42.2%	2.55E-08	4.63E-04
15	FUOM	Enterocytes	0.2500377	79.8%	62.3%	1.39E-06	2.54E-02
16	SPINK4	Paneth cells	1.1023833	36.6%	15.4%	5.44E-08	9.89E-04

16	SLC12A2	Crypt cells	1.018017	62.2%	36.6%	6.81E-09	1.24E-04
16	KRT7	Goblet cells	0.91287	100.0%	90.6%	1.93E-14	3.51E-10
16	G0S2	Enterocytes	0.779919	35.4%	15.2%	1.91E-07	3.47E-03
16	KLF3	Tuft cells	0.7397934	86.6%	47.1%	5.38E-18	9.78E-14
16	ELF3	Enterocytes	0.7356122	100.0%	83.1%	5.94E-18	1.08E-13
16	MMP7	Paneth cells	0.7179741	100.0%	76.6%	3.62E-19	6.58E-15
16	CFD	Paneth cells	0.7138688	50.0%	25.1%	4.33E-09	7.87E-05
16	CTNNB1	Paneth cells	0.6922666	79.3%	46.9%	1.51E-12	2.74E-08
16	SOX9	Tuft cells	0.6891953	87.8%	48.1%	4.09E-16	7.44E-12
16	PMEPA1	Enterocytes	0.6824712	89.0%	54.8%	4.47E-15	8.13E-11
16	CTSD	Microfold cells	0.6804099	98.8%	84.6%	5.87E-12	1.07E-07
16	KCNE3	Crypt cells	0.6510025	62.2%	36.2%	1.88E-08	3.43E-04
16	AHR	Microfold cells	0.6146585	84.1%	35.2%	2.21E-20	4.02E-16
16	COL18A1	Enterocytes	0.5819668	28.0%	14.9%	7.93E-05	1.00E+00
16	IRF2	Microfold cells	0.5532893	65.9%	28.7%	2.22E-14	4.03E-10
16	PRSS1	Paneth cells	0.5437518	37.8%	19.0%	7.48E-06	1.36E-01
16	TRNP1	Enteroendocrine cells	0.5418598	80.5%	53.5%	3.67E-09	6.67E-05
16	MUC4	Goblet cells	0.5353656	64.6%	26.0%	1.51E-14	2.74E-10
16	KLF5	Enterocytes	0.5313557	98.8%	59.4%	9.82E-18	1.79E-13
16	VNN1	Enterocytes	0.5204808	58.5%	29.8%	2.26E-09	4.11E-05
16	PPARG	Enterocytes	0.5045623	76.8%	50.3%	6.94E-10	1.26E-05
16	KCNN4	Paneth cells	0.501225	58.5%	27.0%	1.44E-11	2.62E-07
16	NFIB	Microfold cells	0.4880876	56.1%	23.3%	1.55E-12	2.81E-08
16	EHF	Enterocytes	0.4623502	70.7%	32.3%	1.53E-13	2.78E-09
16	ANXA5	Microfold cells	0.4540507	98.8%	71.9%	9.35E-12	1.70E-07
16	NFIC	Microfold cells	0.4328474	50.0%	18.0%	1.10E-13	2.00E-09
16	ALCAM	Enteroendocrine cells	0.4188159	58.5%	21.9%	4.75E-14	8.64E-10
16	IL13RA1	Tuft cells	0.4108764	61.0%	30.6%	2.67E-09	4.85E-05
16	LYZ	Paneth cells	0.3942749	100.0%	96.5%	3.53E-11	6.42E-07
16	PLA2G2A	Paneth cells	0.3924946	47.6%	14.9%	1.96E-16	3.57E-12
16	AGR2	Paneth cells	0.3304038	100.0%	90.7%	8.28E-05	1.00E+00
16	CDH17	Enterocytes	0.3127759	76.8%	58.7%	6.61E-05	1.00E+00
16	FERMT1	Enterocytes	0.3113866	68.3%	44.2%	8.70E-06	1.58E-01
16	TMC4	Enterocytes	0.3093676	70.7%	48.3%	2.92E-05	5.30E-01

16	PDHA1	Enterocytes	0.3070068	53.7%	27.2%	2.43E-07	4.41E-03
16	CCK	Enteroendocrine cells	0.3061564	50.0%	27.3%	8.15E-06	1.48E-01
16	CD24	Paneth cells	0.301182	100.0%	90.6%	4.98E-05	9.05E-01
16	EPHB2	Crypt cells	0.2886267	30.5%	10.2%	2.00E-09	3.64E-05
16	ALOX5	Tuft cells	0.2852735	47.6%	25.9%	6.54E-06	1.19E-01
16	GALNT12	Goblet cells	0.2807799	46.3%	20.6%	5.49E-08	9.98E-04
16	CXCL16	Microfold cells	0.2742985	76.8%	53.2%	7.74E-06	1.41E-01
16	KLF6	Tuft cells	0.2659476	89.0%	58.3%	1.92E-08	3.49E-04
16	TULP4	Microfold cells	0.2658237	37.8%	20.0%	9.03E-05	1.00E+00
16	DPP4	Enterocytes	0.2623695	28.0%	11.2%	1.52E-06	2.77E-02
16	CTSH	Microfold cells	0.261104	58.5%	40.5%	4.64E-04	1.00E+00
16	ATG16L1	Paneth cells	0.254944	31.7%	14.2%	4.28E-06	7.78E-02
16	LGR4	Paneth cells	0.2531563	56.1%	28.1%	2.36E-07	4.29E-03
16	HNF4A	Enterocytes	0.2512606	46.3%	27.0%	5.13E-05	9.33E-01

Supplementary material S8. Number and percentage of epithelial cell types occupying in the Ctrl and CD enteroids treated with and without IFN-γ.

			Ctrl enteroids					CD enteroids				
			IFN-γ 0 pg/mL		IFN-γ 100 pg/mL			IFN-γ 0 pg/mL		IFN-γ 100 pg/mL		
	Number	% of total analyzed cells	Number	% of cells in IFN-γ-free Ctrl enteroids	Number	% of cells in IFN-γ-treated Ctrl enteroids	*p*-value	Number	% of cells in IFN-γ-free CD enteroids	Number	% of cells in IFN-γ-treated CD enteroids	*p*-value
Intestinal stem cells	1,434	13%	487	11%	318	20%	< 0.0001	322	11%	307	13%	0.0147
Proliferating cells	353	3%	227	5%	14	1%	< 0.0001	98	3%	14	1%	< 0.0001
Enterocytes	1,060	9%	302	7%	66	4%	0.0004	602	21%	90	4%	< 0.0001
Goblet cells	1,581	14%	461	10%	452	29%	< 0.0001	287	10%	381	17%	< 0.0001
Paneth cells	707	6%	296	6%	74	5%	0.0116	220	8%	117	5%	0.0003
Unclassified	6,181	55%	2,781	61%	635	41%	< 0.0001	1,371	47%	1,394	61%	< 0.0001
Total	11,316	100%	4,554	100%	1,559	100%		2,900	100%	2,303	100%	

Supplementary material S9. Determination of therapeutic concentration of upadacitinib. Bright field microscopic image of human enteroids treated with IFN-γ 100 pg/mL and different concentrations of upadacitinib.

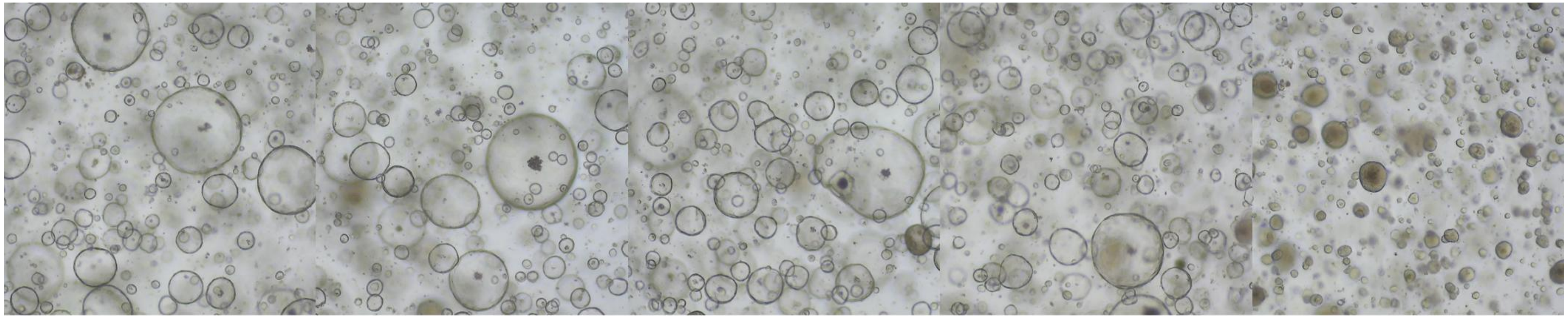

1. Control entroids

2. IFN-γ 100 pg/ml

3. IFN-γ 100 pg/ml + upadacitinib 0.35 μg/ml ≈ 1 μM

4. IFN-γ 100 pg/ml + upadacitinib 3.5 μg/ ml ≈ 10 μM

5. IFN-γ 100 pg/ml + upadacitinib 35 μg/ ml ≈ 100 μM

Supplementary material S10. mTOR and autophagy-related gene expression in Ctrl and CD treated with and without IFN-γ.

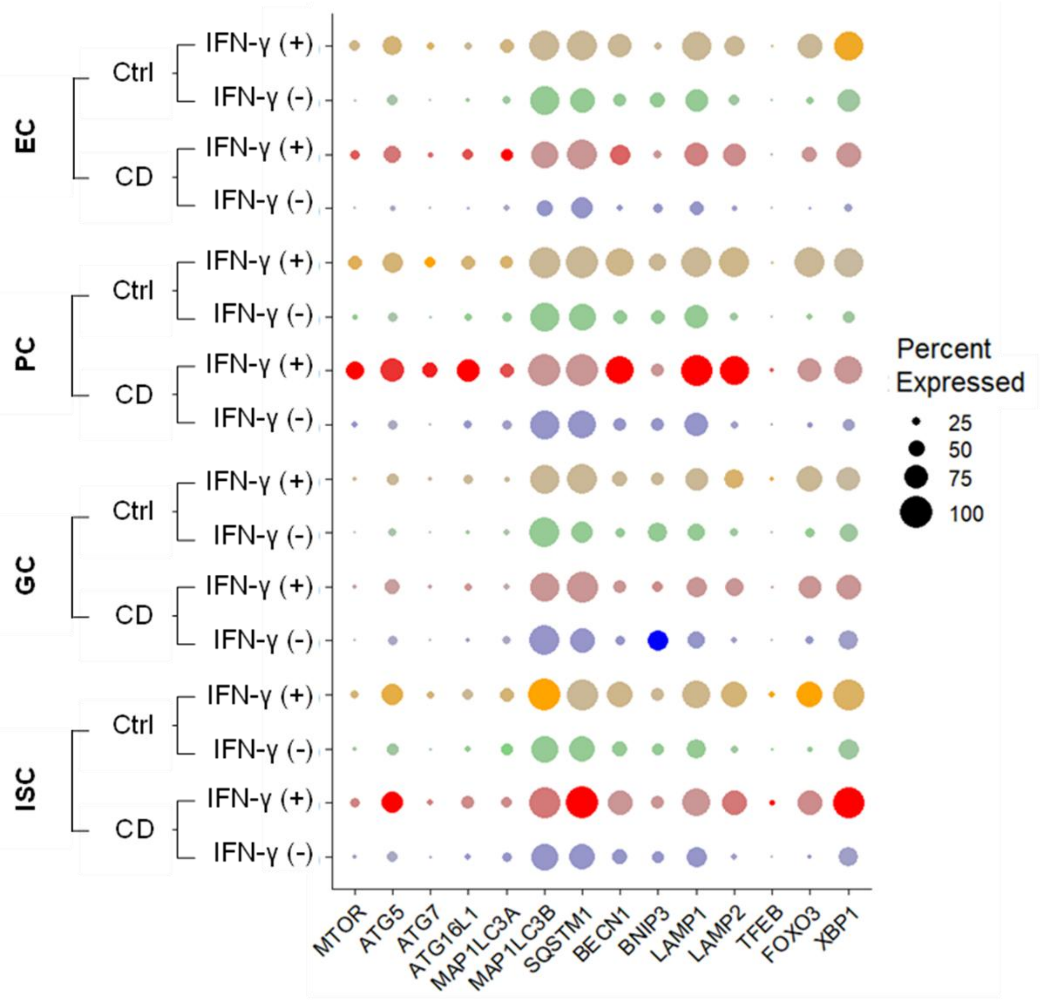

Supplement: Supplementary file 1 [file DataSheet1.pdf]
